# Mapping information-rich genotype-phenotype landscapes with genome-scale Perturb-seq

**DOI:** 10.1016/j.cell.2022.05.013

**Published:** 2022-06-09

**Authors:** Joseph M. Replogle, Reuben A. Saunders, Angela N. Pogson, Jeffrey A. Hussmann, Alexander Lenail, Alina Guna, Lauren Mascibroda, Eric J. Wagner, Karen Adelman, Gila Lithwick-Yanai, Nika Iremadze, Florian Oberstrass, Doron Lipson, Jessica L. Bonnar, Marco Jost, Thomas M. Norman, Jonathan S. Weissman

**Affiliations:** 1 Medical Scientist Training Program, University of California, San Francisco, San Francisco, CA 94158, USA; 2 Tetrad Graduate Program, University of California, San Francisco, San Francisco, CA 94158, USA; 3 Department of Cellular and Molecular Pharmacology, University of California, San Francisco, San Francisco, CA 94158, USA; 4 Howard Hughes Medical Institute, Massachusetts Institute of Technology, Cambridge, MA 02142, USA; 5 Whitehead Institute for Biomedical Research, Massachusetts Institute of Technology, Cambridge, MA 02142, USA; 6 Department of Biochemistry & Molecular Biology, The University of Texas Medical Branch at Galveston, Galveston, TX 77555, USA; 7 Department of Biochemistry & Biophysics, University of Rochester School of Medicine and Dentistry, Rochester, NY 14642, USA; 8 Department of Biological Chemistry and Molecular Pharmacology, Blavatnik Institute, Harvard Medical School, Boston, MA 02115, USA; 9 Ultima Genomics, Newark, CA 94560, USA; 10 Department of Microbiology, Harvard Medical School, Boston, MA 02115, USA; 11 Program for Computational and Systems Biology, Sloan Kettering Institute, Memorial Sloan Kettering Cancer Center, New York, NY 10065, USA; 12 David H. Koch Institute for Integrative Cancer Research, Massachusetts Institute of Technology, Cambridge, MA 02142, USA; 13 Department of Biology, Massachusetts Institute of Technology, Cambridge, MA 02142, USA; 14 These authors contributed equally; 15 Lead contact

## Abstract

A central goal of genetics is to define the relationships between genotypes and phenotypes. High-content phenotypic screens such as Perturb-seq (CRISPR-based screens with single-cell RNA-sequencing readouts) enable massively parallel functional genomic mapping but, to date, have been used at limited scales. Here, we perform genome-scale Perturb-seq targeting all expressed genes with CRISPR interference (CRISPRi) across >2.5 million human cells. We use transcriptional phenotypes to predict the function of poorly characterized genes, uncovering new regulators of ribosome biogenesis (including *CCDC86*, *ZNF236*, and *SPATA5L1*), transcription (*C7orf26*), and mitochondrial respiration (*TMEM242*). In addition to assigning gene function, single-cell transcriptional phenotypes allow for in-depth dissection of complex cellular phenomena—from RNA processing to differentiation. We leverage this ability to systematically identify genetic drivers and consequences of aneuploidy and to discover an unanticipated layer of stress-specific regulation of the mitochondrial genome. Our information-rich genotype-phenotype map reveals a multidimensional portrait of gene and cellular function.

## In brief

Unbiased, genome-scaling profiling of genetic perturbations via single-cell RNA sequencing enables systematic assignment of function to genes and indepth study of complex cellular phenotypes such as aneuploidy and stress-specific regulation of the mitochondrial genome.

## Graphical abstract

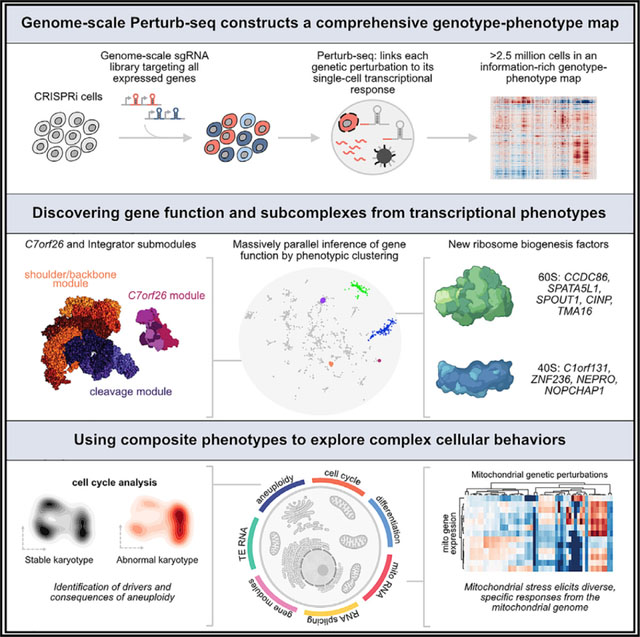

## INTRODUCTION

Mapping the relationship between genetic changes and their phenotypic consequence is critical to understanding gene and cellular function. This mapping is traditionally carried out in one of two ways: a phenotype-centric, ‘‘forward genetic’’ approach that reveals the genetic changes that drive a phenotype of interest or a gene-centric, ‘‘reverse genetic’’ approach that catalogs the diverse phenotypes caused by a defined genetic change.

Recent technological developments have advanced both forward and reverse genetic efforts (Camp et al., 2019). CRISPR tools now enable the deletion, mutation, repression, or activation of genes with ease (Doench, 2018). In forward genetic screens, CRISPR-Cas systems can be used to generate pools of cells with diverse genetic perturbations, which can then be subjected to selection followed by sequencing to assign phenotypes to genetic perturbations. Forward genetic screens are powerful tools for the identification of cancer dependencies, essential cellular machinery, differentiation factors, and suppressors of genetic diseases (Kramer et al., 2018; Tsherniak et al., 2017; Wang et al., 2021, 2015). In parallel, dramatic improvements in molecular phenotyping now allow for single-cell readouts of epigenetic, transcriptomic, proteomic, and imaging information (Stuart and Satija, 2019). Applied to reverse genetics, single-cell profiling can refine the understanding of how select genetic perturbations affect cell types and cell states.

However, both phenotype-centric and gene-centric approaches suffer conceptual and technical limitations. Pooled forward genetic screens typically use low-dimensional phenotypes such as growth or marker expression for selection. The use of simple phenotypes can conflate genes acting via different mechanisms, requiring extensive follow-up studies to disentangle genetic pathways (Przybyla and Gilbert, 2021). Additionally, in forward genetics, serendipitous discovery is constrained by the prerequisite of selecting phenotypes prior to screening. On the other hand, reverse genetic approaches enable the study of multidimensional and complex phenotypes but have typically been restricted in scale to rationally chosen targets, limiting systematic comparisons.

As a solution to these problems, single-cell CRISPR screens simultaneously read out the genetic perturbation and high-dimensional phenotype of individual cells in a pooled format, thus combining the throughput of forward genetics with the rich phenotypes of reverse genetics. Although these approaches initially focused on transcriptomic phenotypes (e.g., Perturb-seq, CROP-seq) (Adamson et al., 2016; Datlinger et al., 2017; Dixit et al., 2016; Jaitin et al., 2016; Replogle et al., 2020), technical advances have enabled their application to epigenetic (Rubin et al., 2019), imaging (Feldman et al., 2019), or multimodal phenotypes (Mimitou et al., 2019). From these rich data, it is possible to identify genetic perturbations that cause a specific behavior as well as to catalog the spectrum of phenotypes associated with each genetic perturbation. Despite the promise of single-cell CRISPR screens, their use has generally been limited to studying at most a few hundred genetic perturbations chosen to address predefined biological questions.

We reasoned that there would be unique value to genome-scale single-cell CRISPR screens. For example, although the number of perturbations scales linearly with experimental cost, the number of pairwise comparisons in a screen—and thus its utility for unsupervised classification of gene function—scales quadratically. Similarly, in large-scale screens, the diversity of perturbations allows exploration of the range of cell states that can be revealed by rich phenotypes. Additionally, as many human genes are well characterized, these genes serve as natural controls to anchor interpretation of comprehensive datasets. Finally, genome-scale experiments could help address fundamental questions, such as what fraction of genetic changes elicit transcriptional phenotypes and how transcriptional responses differ between cell types, with implications for understanding organizing principles of cells.

Here, we report results from the first genome-scale Perturb-seq screens. We use a compact, multiplexed CRISPR interference (CRISPRi) library to assay thousands of loss-of-function genetic perturbations with single-cell RNA sequencing (scRNA-seq) in chronic myeloid leukemia (CML) (K562) and retinal pigment epithelial (RPE1) cell lines. Leveraging the scale and diversity of these perturbations, we show that Perturb-seq can be used to study numerous complex cellular phenotypes—from RNA splicing to differentiation to chromosomal instability (CIN)—and discover gene functions. We then invert our analysis to focus on regulatory networks and uncover unanticipated stress-specific regulation of the mitochondrial genome. In sum, we use Perturb-seq to reveal a multidimensional portrait of cellular behavior, gene function, and regulatory networks that advances the goal of creating comprehensive genotype-phenotype maps.

## RESULTS

### A multiplexed CRISPRi strategy for genome-scale Perturb-seq

**Figure 1.**
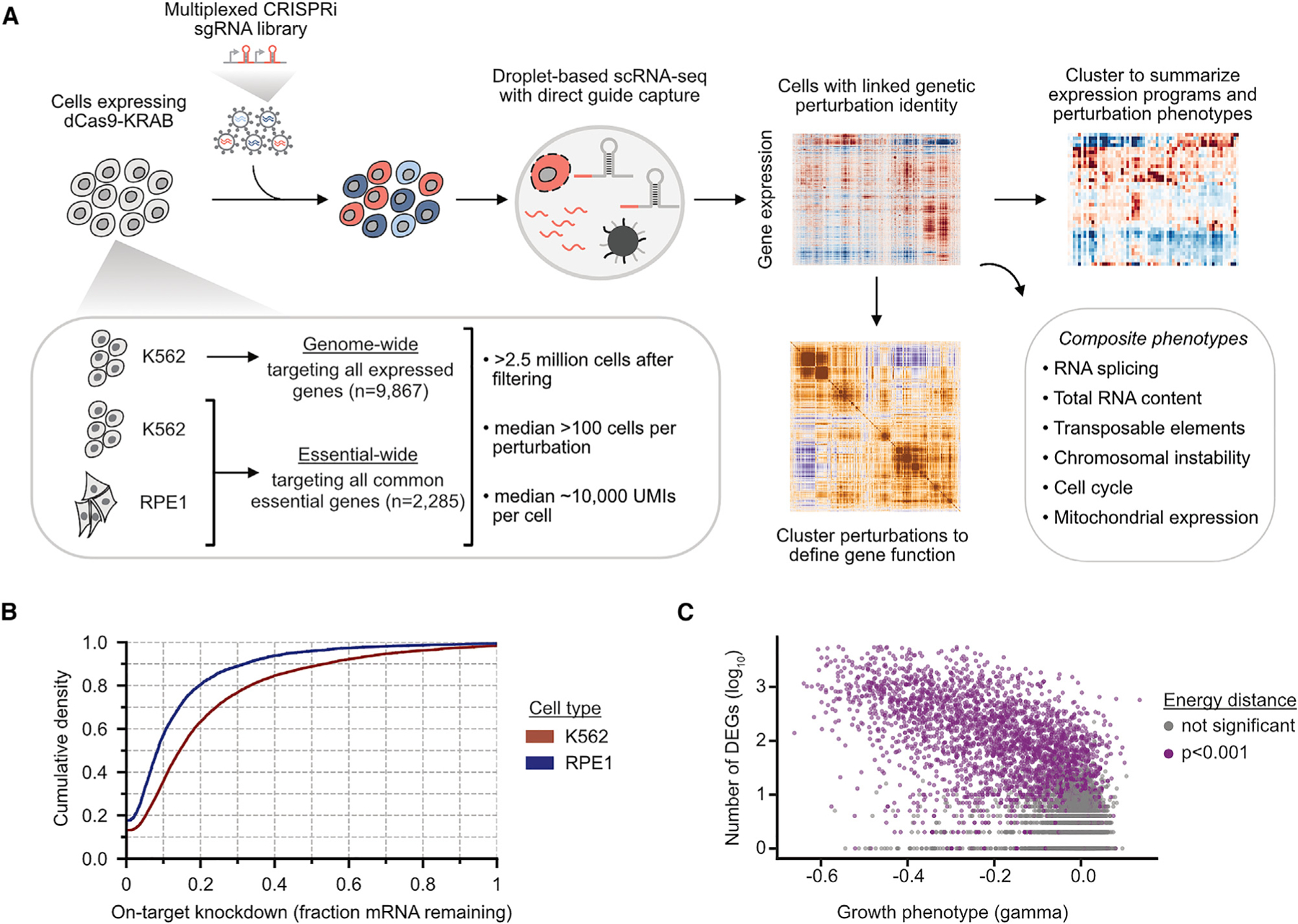
Genome-scale Perturb-seq via multiplexed CRISPRi (A) Experimental strategy. (B) On-target knockdown statistics in K562 cells (red) and RPE1 cells (blue). (C) Comparing growth phenotype versus the number of differentially expressed genes (DEGs) in K562 cells. Growth phenotypes are reported as the log_2_ guide enrichment per cell doubling (gamma). See also Figures S1, S2, and S3.

Perturb-seq uses scRNA-seq to concurrently read out the CRISPR single-guide RNAs (sgRNAs) (i.e., genetic perturbation) and transcriptome (i.e., high-dimensional phenotype) of single cells in a pooled format (Figure 1A). We sought to exploit and understand the rich information content of transcriptomic phenotypes by studying a comprehensive set of genetic perturbations in a given cell type. To enable genome-scale Perturb-seq, we considered key parameters that would increase scalability and data quality, such as the genetic perturbation modality and sgRNA library.

Perturb-seq is compatible with a range of CRISPR-based perturbations. We elected to use CRISPRi for several reasons: (1) Compared with gain-of-function perturbations, a higher proportion of loss-of-function perturbations yield phenotypes in growth and chemical-genetic screens, especially for members of protein complexes (Gilbert et al., 2014; Horlbeck et al., 2016). (2) CRISPRi allows direct measurement of the efficacy of genetic perturbation—knockdown—by scRNA-seq. Exploiting this feature allowed us to target each gene in our library with a single element and empirically exclude unperturbed genes from downstream analysis. (3) CRISPRi tends to yield more homogeneous perturbation than CRISPR knockout, which can generate active in-frame indels (Smits et al., 2019). The relative homogeneity of CRISPRi reduces selection for unperturbed cells, especially when studying essential genes. (4) Unlike CRISPR knockout, CRISPRi does not lead to activation of the DNA damage response which can alter transcriptional signatures (Haapaniemi et al., 2018).

We first optimized our CRISPRi sgRNA libraries for scalability. To maximize CRISPRi efficacy, we used multiplexed CRISPRi libraries in which each element contains two distinct sgRNAs targeting the same gene (Table S1; Replogle et al., 2020). To avoid low representation of sgRNAs targeting essential genes, we performed growth screens and, during oligonucleotide library synthesis, overrepresented constructs that caused strong growth defects (Figures S1A–S1D).

Next, we devised a three-pronged Perturb-seq screening approach encompassing multiple time points and cell types (Figure 1A). As a principal cell line, we studied CML K562 cells engineered to express the CRISPRi effector dCas9-KRAB (Gilbert et al., 2014). In this cell line, we performed two Perturb-seq screens: one targeting all expressed genes sampled 8 days after lentiviral transduction (n = 9,866 genes) and another targeting common essential genes sampled 6 days after transduction (n = 2,057 genes). As a secondary cell line, we used RPE1 cells engineered to express dCas9 fused to a *ZIM3*-derived KRAB domain, which was recently shown to improve CRISPRi transcriptional repression (Alerasool et al., 2020), sampled 7 days after transduction. In contrast to K562 cells, RPE1 cells are a non-cancerous, hTERT-immortalized, near-euploid, adherent, and p53-positive cell line.

We conducted all screens with 10x Genomics droplet-based 3′ scRNA-seq and direct sgRNA capture (Replogle et al., 2020). After excluding cells bearing sgRNAs targeting different genes, which are an expected byproduct of lentiviral recombination between sgRNA cassette or doublet encapsulation during scRNA-seq, we retained >2.5 million high-quality cells with a median coverage of >100 cells per perturbation (Figures S1E–S1G; Table S2). We observed a median target knockdown of 85.5% in K562 cells and 91.6% in RPE1 cells (Figure 1B), confirming the efficacy of our CRISPRi libraries and the fidelity of sgRNA assignment.

### A robust computational framework to detect transcriptional phenotypes

The scale of our experiment provided a unique opportunity to ask what fraction of genetic perturbations cause a transcriptional phenotype. Significant transcriptional phenotypes can take many forms, ranging from altered occupancy of cell states to focused changes in the expression of a small number of target genes. To contend with this diversity, we created a robust framework capable of detecting transcriptional changes. Our experimental design included many control cells bearing diverse non-targeting sgRNAs. These allowed for internal *z*-normalization of expression measurements to correct for batch effects resulting that resulted from parallelized scRNA-seq and sequencing (Data S1 [Figure i]). As Perturb-seq captures single-cell genetic perturbation identities in a pooled format, we can use statistical approaches that treat cells as independent samples. In general, we chose to use conservative, nonparametric statistical tests to detect transcriptional changes rather than making specific assumptions about the underlying distribution of gene expression levels.

First, we examined global transcriptional changes using a permuted energy distance test (see STAR Methods). We compared cells bearing each genetic perturbation with control cells at the level of principal components (approximating global features like cell state and gene expression programs) to test whether cells carrying a given genetic perturbation could have been drawn from the control population. By this metric, 2,987 of 9,608 genetic perturbations targeting a primary transcript (31.1%) compared with 11 of 585 controls (1.9%) caused a significant transcriptional phenotype in K562 cells.

Although sensitive, the energy distance test assays global shifts in expression without providing insight into which specific transcripts are altered. To detect individual differentially expressed genes (DEGs), we applied the Anderson-Darling (AD) test that is sensitive to transcriptional changes in a subset of cells, enabling us to find differences even with incomplete penetrance. By the AD test, 2,935 of 9,608 genetic perturbations targeting a primary transcript (30.5%) compared with 12 of 585 controls (2.1%) caused >10 DEGs in K562 cells. These results were well-correlated between time points and cell types (Figures S2A and S2B; Tables S2) and concordant with the energy distance test (78.7% concordance by Jaccard index).

We then explored features of genetic perturbations that predict the likelihood of causing a transcriptional phenotype. The strength of transcriptional response was correlated with the growth phenotype (Spearman’s ρ = −0.51) with 86.6% of essential genetic perturbations leading to a significant transcriptional response (Figure 1C; Figures S2C and S2D). Nonetheless, a substantial number of genetic perturbations that cause a transcriptional phenotype have a negligible growth phenotype (n = 771; Figure S2E), indicating that many genetic perturbations influence cell state but not growth or survival. Genes whose knockdown caused strong transcriptional phenotypes were more likely to be highly expressed, to have an annotated subcellular localization, and to be components of core protein complexes (Figures S2F–S2L).

As some of our genetic perturbations did not yield strong on-target knockdown, our estimate of the fraction of genetic perturbations that cause a transcriptional phenotype is likely a lower bound. Although some phenotypes may result from off-target effects, Perturb-seq allows direct detection of off-target activities such as neighboring gene knockdown. Consistent with earlier studies (Rosenbluh et al., 2017), ~7.5% of perturbations caused knockdown of a neighboring gene, but neighbor gene knockdown was not enriched in perturbations with a negligible growth defect that produced a transcriptional phenotype (Figure S3).

### Annotating gene function from transcriptional phenotypes

Previous Perturb-seq screens focused on targeted sets of perturbations, such as genes identified in forward genetic screens. Our screen targeting all expressed genes in K562 cells presented an opportunity to assess how well transcriptional phenotypes can resolve gene function when used in an unbiased manner.

**Figure 2.**
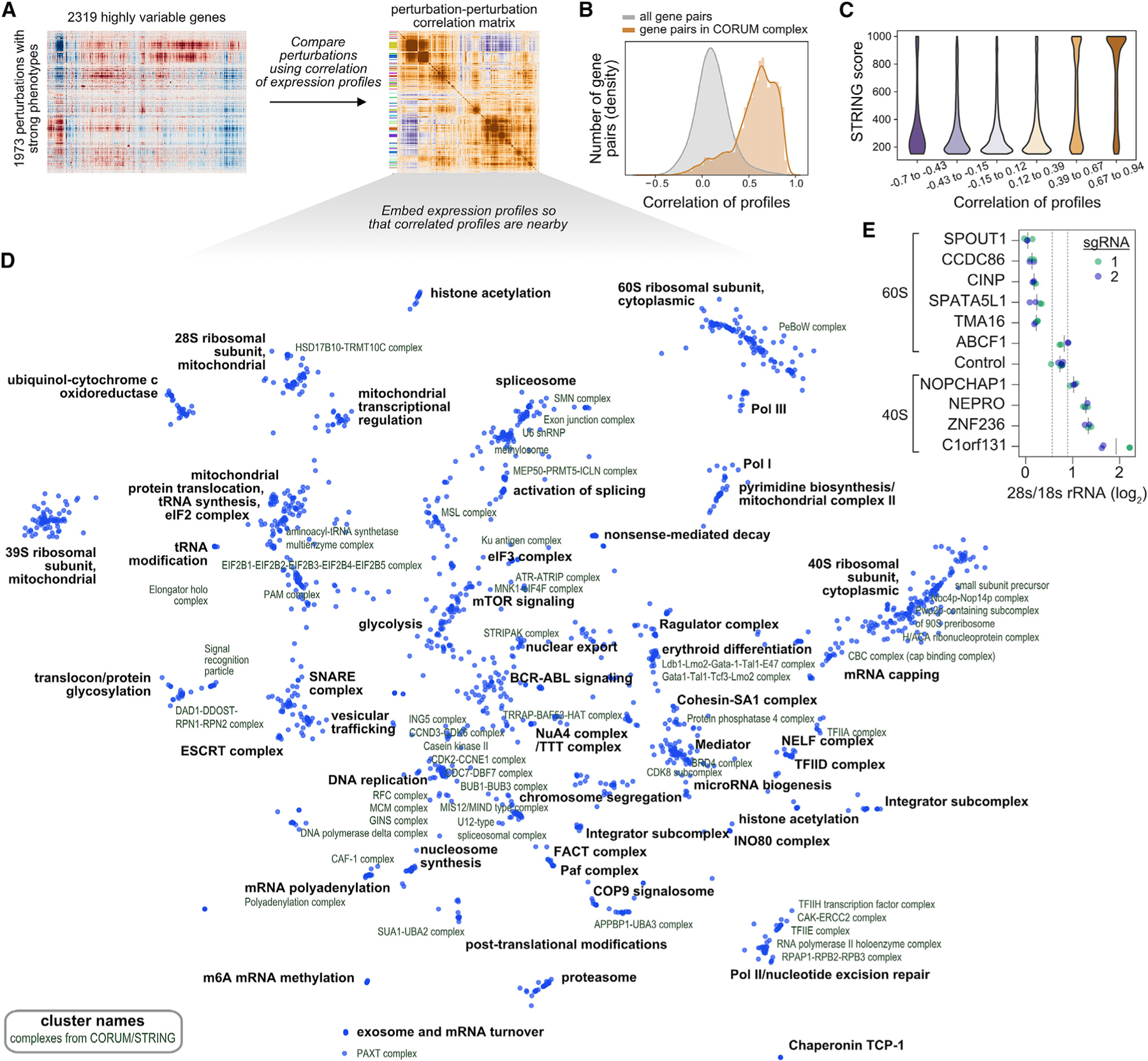
Data-driven inference of gene function from transcriptional phenotypes (A) Analysis schematic. Genetic perturbations that elicited strong responses were clustered by correlation of expression of highly variable genes. (B) Distributions of pairwise expression profile correlations among all possible gene-gene pair versus among genes in 327 CORUM3.0 protein complexes that have at least two thirds of complex subunits within the dataset. (C) Kernel density estimates (KDEs) of STRING scores divided into bins based on expression profile correlation. (D) Minimum distortion embedding where each dot represents a genetic perturbation. Manual annotations (black labels) of cluster function are placed near the median location of genes within the cluster. CORUM complexes or STRING clusters (green labels) are annotated. (E) Quantification of 28S to 18S rRNA ratio after knockdown of indicated genes by CRISPRi. rRNA was measured by Bioanalyzer in biological duplicate with two distinct sgRNAs per gene (green and blue; solid gray lines represent mean). Dotted gray lines represent two standard deviations above and below the mean of non-targeting controls. See also Figure S4.

We focused on a subset of 1,973 perturbations that had strong transcriptional phenotypes (Figure 2A). Because related perturbations could have different magnitudes of effect, we used the correlation between mean expression profiles as a scale-invariant metric of similarity. To assess the extent to which correlated expression profiles between genetic perturbations indicated common function, we compared our results with two curated sources of biological relationships. First, among the 1,973 targeted genes, there were 327 protein complexes from CORUM3.0 with at least two thirds of the complex members present, representing 14,165 confirmed protein-protein interactions (Giurgiu et al., 2019). The corresponding expression profile correlations were stronger (median r = 0.61) than the background distribution of all possible gene pairs (median r = 0.10) (Figure 2B). Second, high correlation between expression profiles was strongly associated with high STRING protein-protein interaction confidence scores (Figure 2C; Szklarczyk et al., 2019).

We next performed an unbiased clustering of similar perturbations within the dataset. We identified 64 discrete clusters and annotated their function using CORUM, STRING, and manual searches. To visualize the dataset, we constructed a minimum distortion embedding that places genes with correlated expression profiles nearby (Figure 2D). The clusters and embedding showed clear organization by biological function spanning an array of processes including: chromatin modification; transcription; mRNA splicing, capping, polyadenylation, and turnover; nonsense-mediated decay; translation; posttranslational modification, trafficking, and degradation of proteins; central metabolism; mitochondrial transcription and translation; DNA replication; cell division; microRNA biogenesis; and major signaling pathways (Table S3).

Next, we compared the similarity of transcriptional phenotypes between all three Perturb-seq datasets. For K562 cells sampled at day 8 versus day 6, both the relationships between perturbations (cophenetic correlation = 0.82) and phenotypes (median r = 0.50) were highly similar (Figures S4A and S4B). By contrast, the K562 and RPE1 datasets had more divergent relationships (cophenetic correlation = 0.37) and phenotypes (median r = 0.23) (Figures S4B–S4E).

In our dataset, perturbation of many poorly annotated genes led to similar transcriptional responses to genes of known function, naturally predicting a role for these uncharacterized genes. To test these predictions, we selected ten poorly annotated genes whose perturbation response correlated with subunits and biogenesis factors of either the large or small subunit of the cytosolic ribosome (Figure S4F). This included genes that had no previous association with ribosome biogenesis (*CCDC86*, *CINP*, *SPATA5L1*, *ZNF236*, and *C1orf131*) as well as genes that had not been associated with functional defects in a particular subunit (*SPOUT1*, *TMA16*, *NOPCHAP1*, *ABCF1*, and *NEPRO*). CRISPRi-mediated depletion of nine of the ten candidate factors led to substantial defects in ribosome biogenesis, with the exception of *ABCF1*, as assessed by the ratio of 28S to 18S rRNA (Figure 2E). In every case, the affected ribosomal subunit corresponded to the Perturb-seq clustering across two independent sgRNAs. Although this study was in progress, another group identified C1orf131 as a structural component of the pre-A1 small subunit processome by cryo-EM, complementing our functional work (Singh et al., 2021). This validation suggests that many poorly characterized genes can be assigned functional roles through Perturb-seq, although a subset of these relationships may be explained by off-target effects (Figure S4G and S4H).

### Delineating functional modules of the Integrator complex

**Figure 3.**
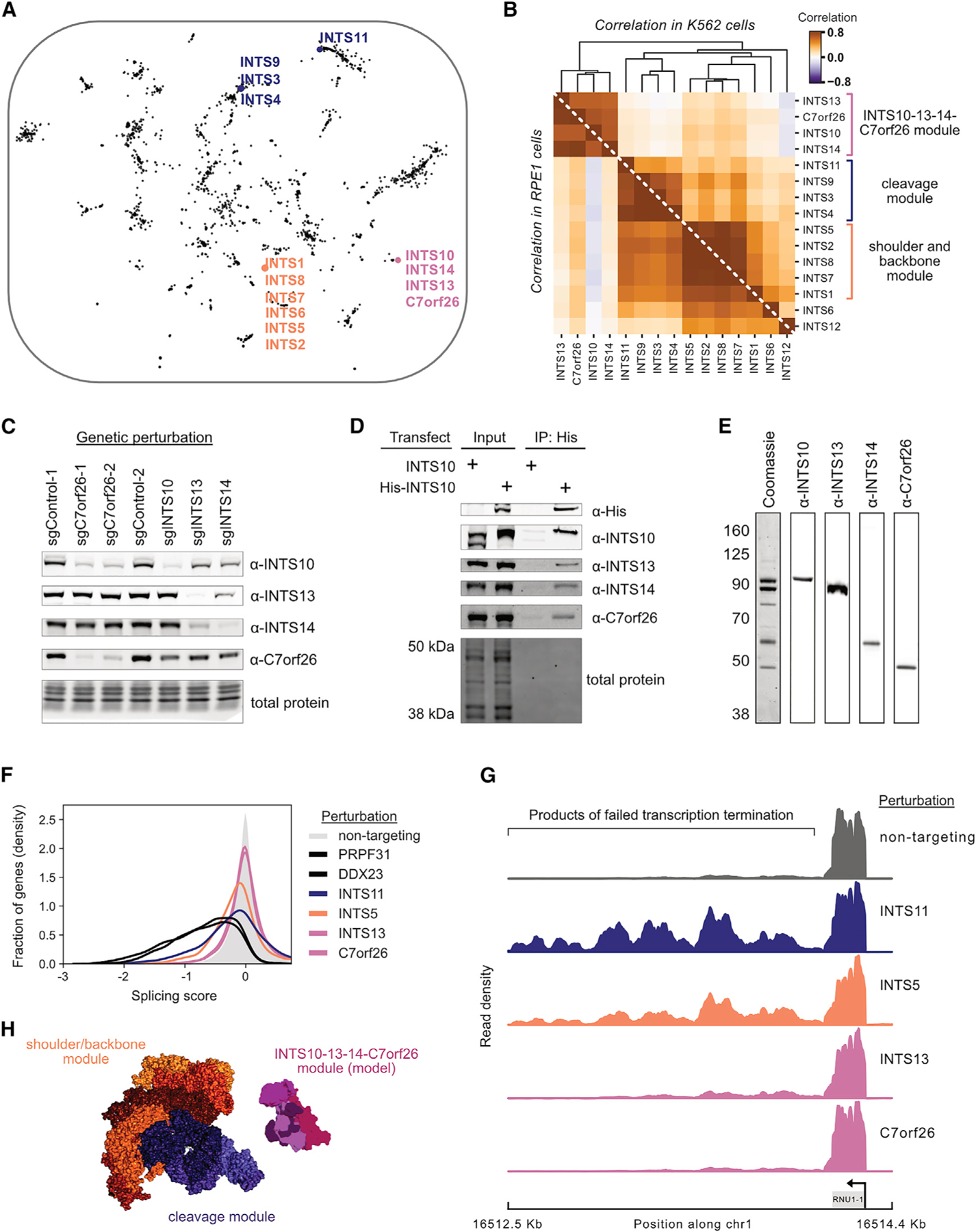
Discovery of a novel gene member and functional submodules of the Integrator complex (A) Location of Integrator complex members in the minimum distortion embedding. (B) Relationship between Integrator complex members and *C7orf26* in K562 cells and RPE1 cells. The heatmap shows the Pearson correlation between gene expression profiles of Integrator complex members. (C) Co-depletion of Integrator complex members. Integrator complex members were depleted by CRISPRi in K562 cells. Lysates were probed by western blot. (D) Co-immunoprecipitation of endogenous C7orf26 with His-INTS10. HEK293T were transfected with His-INTS10 or INTS10. Lysates were affinity purified and probed by western blot. (E) Purification of a INTS10-13-14-C7orf26 complex. His-INTS10, INTS13, INTS14, and C7orf26 were overexpressed, affinity purified, separated via SEC, and probed by western blot. (F) Effects of Integrator modules on gene-level splicing scores from Perturb-seq data. (G) Density of PRO-seq reads at the snRNA *RNU1-1* locus mapping actively engaged RNA polymerase II. (H) Structure of the Integrator complex colored by Perturb-seq functional modules. The endonuclease (blue) and shoulder/backbone (orange) modules were obtained from the cryo-EM structure (Zheng et al., 2020). The model of the newly discovered INTS10-13-14-C7orf26 module was built by docking the crystal structure of INTS13-INTS14 (Sabath et al., 2020) with an AlphaFold multimeric model of INTS10 and C7orf26.

In general, perturbations to members of known protein complexes produced similar transcriptional phenotypes in our data. Therefore, we were surprised that knockdown of the 14 core subunits of Integrator, a metazoan-specific essential nuclear complex with roles in small nuclear RNA (snRNA) biogenesis and transcription termination, led to variable responses (Figure 3A; Kirstein et al., 2021). *INTS1*, *INTS2*, *INTS5*, *INTS7*, and *INTS8* formed a tight cluster that weakly correlated with *INTS6* and *INTS12* (Figure 3B; Data S2 [Figure i]). Separately, *INTS3*, *INTS4*, *INTS9*, and *INTS11* clustered together alongside splicing regulators. Finally, *INTS10*, *INTS13*, and *INTS14* formed a discrete cluster together with *C7orf26*, an uncharacterized gene.

These functional modules mirror the architecture observed in recent structures (Fianu et al., 2021; Zheng et al., 2020). The INTS1-2-5-7-8 module contained the subunits identified as the structural shoulder and backbone. The INTS3-4-9-11 module contained the structural cleavage subunits. Although INTS10, INTS13, and INTS14 were not resolved in recent cryo-EM Integrator structures, these subunits have been identified as a stable biochemical subcomplex (Pfleiderer and Galej, 2021; Sabath et al., 2020).

Integrator is well studied; hence, we were intrigued by the clustering of the uncharacterized gene *C7orf26* with Integrator subunits 10, 13, and 14. To explore this, we tested whether loss of C7orf26 impacted Integrator subunit abundance. CRISPRi-mediated depletion of C7orf26 destabilized INTS10 (Figure 3C). Pulldown of His-INTS10 from cell lysates recovered endogenous C7orf26 alongside INTS13 and INTS14 (Figure 3D), indicative of a biochemical interaction consistent with previous reports (Boeing et al., 2016; Malovannaya et al., 2010). Overexpression of C7orf26 with INTS10, INTS13, and INTS14 enabled the purification of a stable INTS10-13-14-C7orf26 complex by size-exclusion chromatography (Figure 3E; Data S2 [Figures i and ii]). We also detected an interaction between the Drosophila C7orf26 ortholog and fly Integrator and observed co-essentiality between C7orf26 and INTS10, INTS13, and INTS14 in the Cancer Dependency Map (Data S2 [Figure iii]) (Pan et al., 2022; Wainberg et al., 2021). These results suggest that C7orf26 is a core subunit of an INTS10-13-14-C7orf26 Integrator module.

We sought to better understand the distinct transcriptional phenotypes induced by loss of Integrator modules. As comparison of DEGs between modules did not reveal function (Data S2 [Figure i]), we next explored the canonical role of Integrator using changes in splicing as a proxy for snRNA biogenesis defects. We examined splicing by comparing the ratio of unspliced with spliced reads for each gene in our Perturb-seq data. Validating our approach, depletion of known splicing factors as well as subunits of the cleavage and shoulder/backbone modules led to gross splicing defects (Figure 3F). By contrast, depletion of subunits of the INTS10-13-14-C7orf26 module did not cause a substantial splicing defect. To directly test the effect of the INTS10-13-14-C7orf26 module on snRNA biogenesis, we used precision run-on sequencing (PRO-seq) to probe active RNA-polymerase positioning and confirmed that extended knockdown of the cleavage and backbone/shoulder modules, but not *INTS10*, *INTS13*, or *C7orf26*, caused a dramatic increase in transcriptional readthrough past the 3′ cleavage site of snRNAs (Figure 3G; Data S2 [Figure i]).

Thus, INTS10-13-14-C7orf26 represents a functionally and biochemically distinct module of Integrator, consistent with concurrent studies (Figure 3H; Funk et al., 2021; Pan et al., 2022; Wainberg et al., 2021). Although Integrator has been subjected to extensive structural analyses, it has been difficult to resolve the INTS10-13-14 components in relation to the rest of the complex, and inclusion of C7orf26 may facilitate future structural efforts. We propose that *C7orf26* be renamed *INTS15*.

### Data-driven definition of transcriptional programs

**Figure 4.**
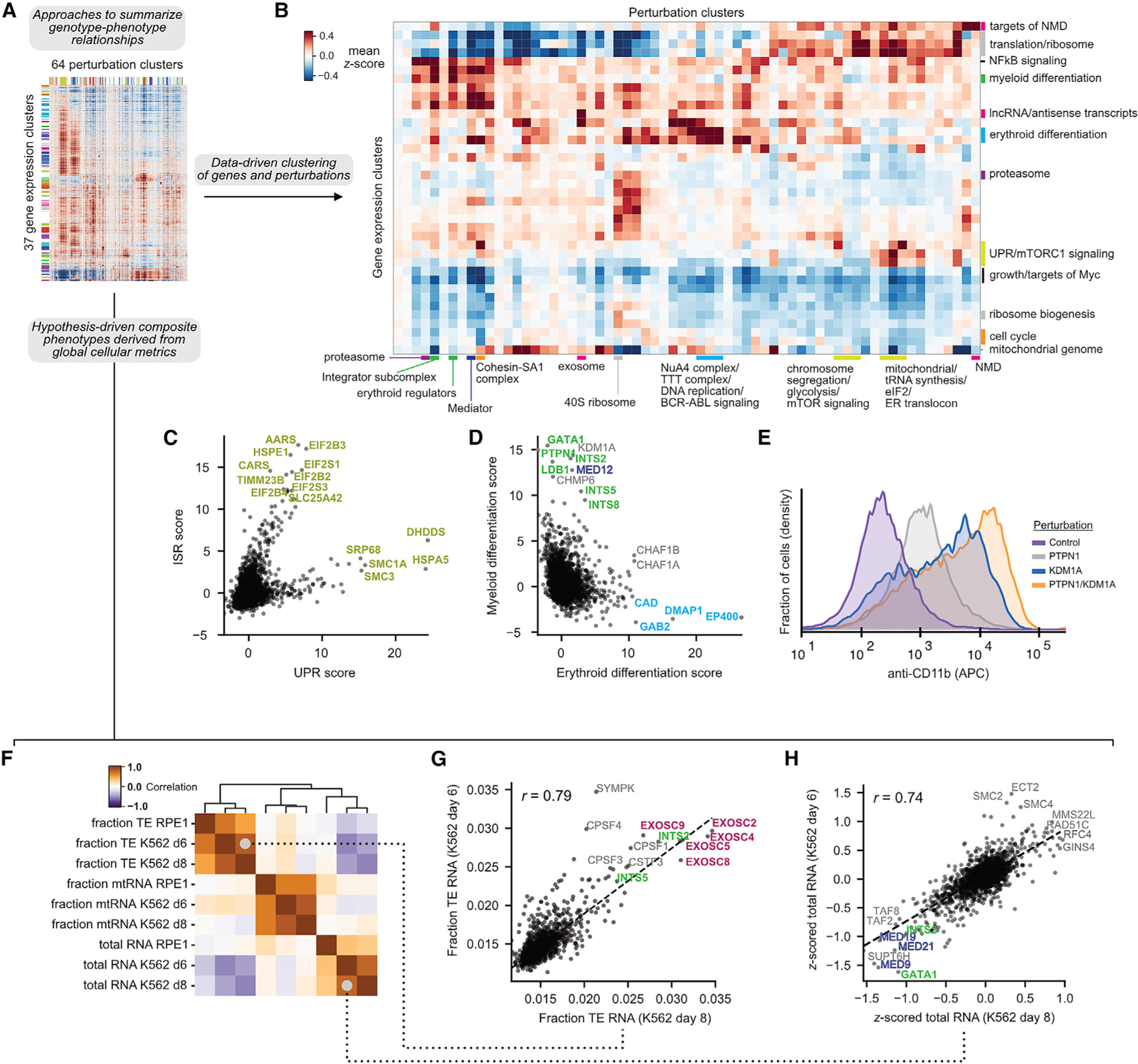
Summarizing genotype-phenotype relationships with Perturb-seq (A) Analysis schematic. (B) Heatmap of the genotype-phenotype map. The heatmap represents the mean *Z* scored expression for gene expression and perturbation clusters labeled with manual annotations. (C) Comparison of ISR and UPR scores for perturbations. (D) Comparison of erythroid and myeloid differentiation scores for genetic perturbations. Genetic perturbations are colored to reflect cluster identity. (E) CD11b surface expression (measured by flow cytometry) upon knockdown of *PTPN1* or *KDM1A* in K562 cells. (F) Correlation of composite phenotypes across time points and cell types. Fraction TE represents the number of non-intronic reads mapped to TEs over total, averaged over all cells bearing each perturbation. Fraction mtRNA represents the mean number of reads mapped to mitochondrial genome protein-coding genes over total. Total RNA represents the mean total RNA content. (G) Comparison of TE expression across time points. (H) Comparison of total RNA content across time points. See also Figure S5.

Although clustering can organize genetic perturbations into pathways or complexes, it does not reveal the functional consequences of perturbations. To summarize the genotype-phenotype relationships in our data, we (1) clustered genes into expression programs based on their co-regulation, (2) clustered perturbations based on their transcriptional profiles, and (3) computed the average activity of each gene expression program within each perturbation cluster (Figures 4A and 4B; Figure S5A; Table S3; see STAR Methods). This map uncovered many known expression programs associated with perturbations, including upregulation of proteasomal subunits due to proteasome dysfunction (Radhakrishnan et al., 2010), activation of NF-κB signaling upon loss of ESCRT proteins (Mamińska et al., 2016), and upregulation of cholesterol biosynthesis in response to defects in vesicular trafficking (Luo et al., 2020). Our analysis also delineated the canonical branches of the cellular stress response into the unfolded protein response (UPR), activated by the loss of ER-resident chaperones and translocation machinery, and integrated stress response (ISR), activated by loss of mitochondrial proteins, aminoacyl-tRNA synthetases, and translation initiation factors (Figure 4C; Adamson et al., 2016).

Interestingly, our clustering uncovered many perturbations that drove the expression of markers of erythroid or myeloid differentiation, consistent with the multilineage potential of K562 cells (Figure 4D; Leary etal., 1987). As expected, loss of central regulators of erythropoiesis (*GATA1*, *LDB1*, *LMO2*, and *KDM1A*) caused myeloid differentiation, whereas knockdown of *BCR-ABL* and its adaptor *GAB2* induced erythroid differentiation (Orkin and Zon, 2008). Next, we investigated the differentiation effect of selectively essential genes, which could be promising targets for differentiation therapy, analogous to KDM1A (Maes et al., 2018; Yu et al., 2021). We observed that loss of *PTPN1*, a tyrosine phosphatase selectively essential in K562 cells, drove myeloid differentiation. In targeted experiments, we found that combined knockdown of *PTPN1* and *KDM1A* caused a substantial increase in differentiation and growth defect compared with knockdown of either gene individually, suggesting that these targets act via different cellular mechanisms (Figure 4E; Figure S5B). These results highlight the utility of rich phenotypes for understanding differentiation as well as nominating therapeutic targets.

### Hypothesis-driven study of composite phenotypes

Our scRNA-seq readout also allows us to study ‘‘composite phenotypes’’ that integrate data from across the transcriptome, such as total cellular RNA content and the fraction of RNA derived from transposable elements (TEs). We found numerous composite phenotypes under strong genetic control, with reproducible effects across replicates and cell types (Figure 4F).

In the case of TE regulation, two classes of perturbations increased the fraction of TE RNA by affecting broad classes of elements including Alu, L1, and MIR (Figure 4G; Figure S5C). First, loss of exosome subunits increased the fraction of TE RNA, suggesting that transcripts deriving from TEs might be preferentially degraded. Second, loss of the CPSF and Integrator complexes produced a similar phenotype, suggesting that TE RNAs may derive from failure of normal transcription termination.

Turning to total RNA content, we found that loss of regulators of S-phase and mitosis increased the RNA content of cells (Figure 4H). This is consistent with the observation that cells tend to increase their size and thus their RNA content, as they progress through the cell cycle (Figure S5D). By contrast, loss of transcriptional machinery, including general transcription factors, Mediator, and transcription elongation factors, decreased total RNA content. In sum, these analyses show that Perturb-seq enables hypothesis-driven exploration of complex cellular features.

### Exploring genetic drivers and consequences of aneuploidy in single cells

**Figure 5.**
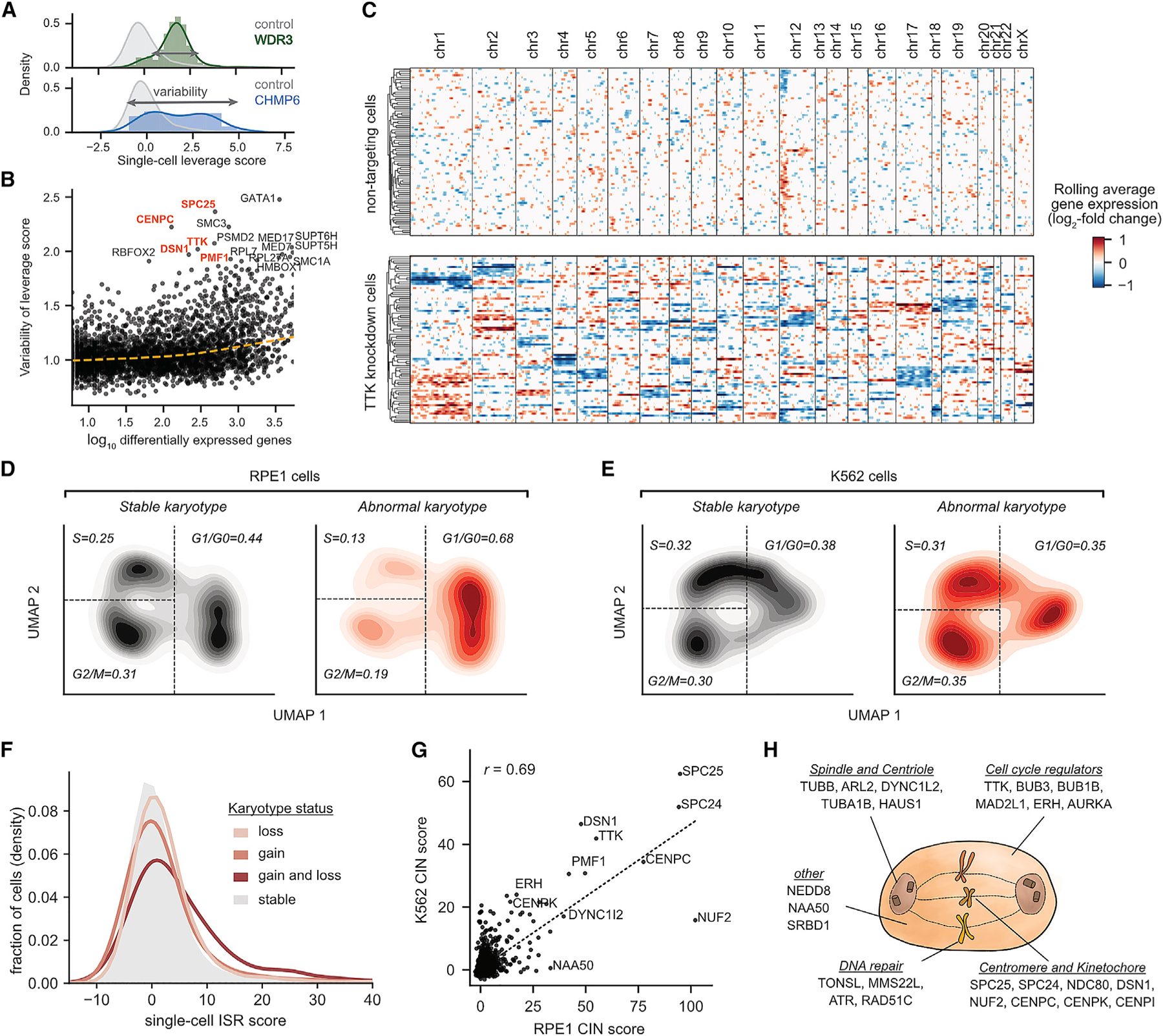
Exploring acute consequences and genetic drivers of aneuploidy in single cells (A) Schematic of heterogeneity statistic. Single-cell leverage scores quantify how outlying each cell is relative to control cells with single-cell heterogeneity quantified as the standard deviation of leverage scores. (B) Identifying heterogeneous perturbations by comparison of single-cell heterogeneity to number of differentially expressed genes. (C) Heatmap of chromosome copy-number inference from Perturb-seq data. For expressed genes, the log-fold change in expression is calculated with respect to the average of control cells, and genes are ordered along the genome. A weighted moving average is used infer copy-number changes (columns) in single cells (rows). Cells are ordered by hierarchical clustering based on correlation of chromosome copy-number profiles. (D and E) Comparison of cell-cycle occupancy upon acute karyotypic changes. Abnormal karyotypic cells were defined as having ≥1 chromosome with evidence of changes in chromosome copy number for >80% of the chromosome length. Cell-cycle occupancy is shown as a 2D KDE of a random subset of 1,000 cells per karyotypic status. (F) Comparison of CIN status on ISR score in RPE1 cells. (G) Comparison of the effect of genetic perturbations on the CIN score across cell types. The perturbation CIN score is calculated as the mean single-cell sum of squared CIN values, *z*-normalized relative to control perturbations. (H) Schematic of a subset of genetic perturbations that drive CIN. See also Figures S6 and S7.

We next reasoned that exploring sources of single-cell heterogeneity could reveal insights that are missed in bulk or averaged measurements. To assess the penetrance of perturbation-induced phenotypes, we applied SVD-based leverage scores as a metric of single-cell phenotypic magnitude (see STAR Methods). Leverage scores quantify how outlying each perturbed cell’s transcriptome is relative to control cells without assuming that perturbations drive a single axis of variation. Supporting this approach, mean leverage scores for each genetic perturbation were correlated with the number of DEGs (Figure S6A, Spearman’s ρ = 0.71) and reproducible across experiments (Figure S6B, Spearman’s ρ = 0.79). We then scored perturbations by the variation in single-cell leverage scores (Figure 5A). Comparing leverage scores across complex subunits revealed evidence for both biological (e.g., subcomplex function or dosage imbalance) and technical (e.g., selection to escape toxic perturbations) sources of phenotypic heterogeneity (Figures S6C–S6F).

Intriguingly, many genes implicated in chromosome segregation were among the top drivers of heterogeneity, including *TTK*, *SPC25*, and *DSN1* (Figure 5B; Musacchio and Salmon, 2007). We hypothesized that the extreme transcriptional variability caused by these genetic perturbations might result from acute changes in the copy number of chromosomes due to mitotic mis-segregation. To explore this, we used inferCNV (Patel et al., 2014) to estimate single-cell DNA copy number along the genome. Consistent with our hypothesis, knockdown of *TTK*, a core component of the spindle assembly checkpoint (Jelluma et al., 2008), led to dramatic changes in chromosome copy number in both intrinsically aneuploid K562 and near-euploid RPE1 cells (Figure 5C; Figure S7A). In RPE1 cells, we found that 61/80 (76%) of *TTK* knockdown cells had karyotypic changes compared with 274/13,140 (2%) of unperturbed cells. Notably, *TTK* knockdown cells bore highly variable karyotypes due to the stochastic gain or loss of chromosomes, accounting for their phenotypic heterogeneity (Figure 5C).

Perturb-seq further allows us to dissect relationships between cellular phenotypes. We were curious how CIN would affect cell-cycle progression in p53-positive RPE1 cells versus p53-deficient K562 cells. Considering all cells in our experiment independent of genetic perturbation, RPE1 cells with abnormal karyotypes tended to arrest in G1 or G0 of the cell cycle (G1 or G0 fraction 0.68 for abnormal karyotype versus 0.44 for stable karyotype), whereas K562 cells with altered karyotypes had less significant shifts in cell-cycle occupancy (Figures 5D and 5E). Within the population of RPE1 cells bearing a chromosome loss, the likelihood of cell cycle arrest directly depended on the magnitude of karyotypic abnormality (Figure S7B). Additionally, cells with the most severe karyotypic changes—those bearing both chromosome gains and losses—had marked upregulation of the ISR (Figures 5F and S7C). These results are consistent with models in which cell-cycle checkpoints are activated by the secondary consequences of aneuploidy (e.g., DNA damage or proteostatic stress) rather than changes in chromosome number per se (Santaguida and Amon, 2015; Santaguida et al., 2017).

Finally, to systematically identify drivers of CIN, we assigned a score to each perturbation based on the average magnitude of induced karyotypic abnormalities. CIN scores were strongly correlated across K562 and RPE1 cell lines (r = 0.69) and identified many known regulators of chromosome segregation (Figure 5G). Remarkably, we uncovered CIN regulators with diverse cellular roles, from cytoskeletal components to DNA repair machinery (Figure 5H; Table S2). This analysis also shows the potential of single-cell CRISPR screens to dissect phenotypes that were not predefined endpoints of the experiment.

### Discovery of stress-specific regulation of the mitochondrial genome

Mitochondria arose from the engulfment and endosymbiotic evolution of an ancestral alphaproteobacterium (Friedman and Nunnari, 2014). Although the majority (~99%) of mitochondrially localized proteins are encoded in the nuclear genome, mitochondria contain a small (~16.6 kb) remnant of their ancestral genome encoding 2 rRNAs, 22 tRNAs, and 13 protein-coding genes in humans. An open question is how expression of the nuclear and mitochondrial genomes is coordinated to cope with mitochondrial stress (Quirós et al., 2016). The scale of our experiment provided a unique opportunity to investigate this question.

**Figure 6.**
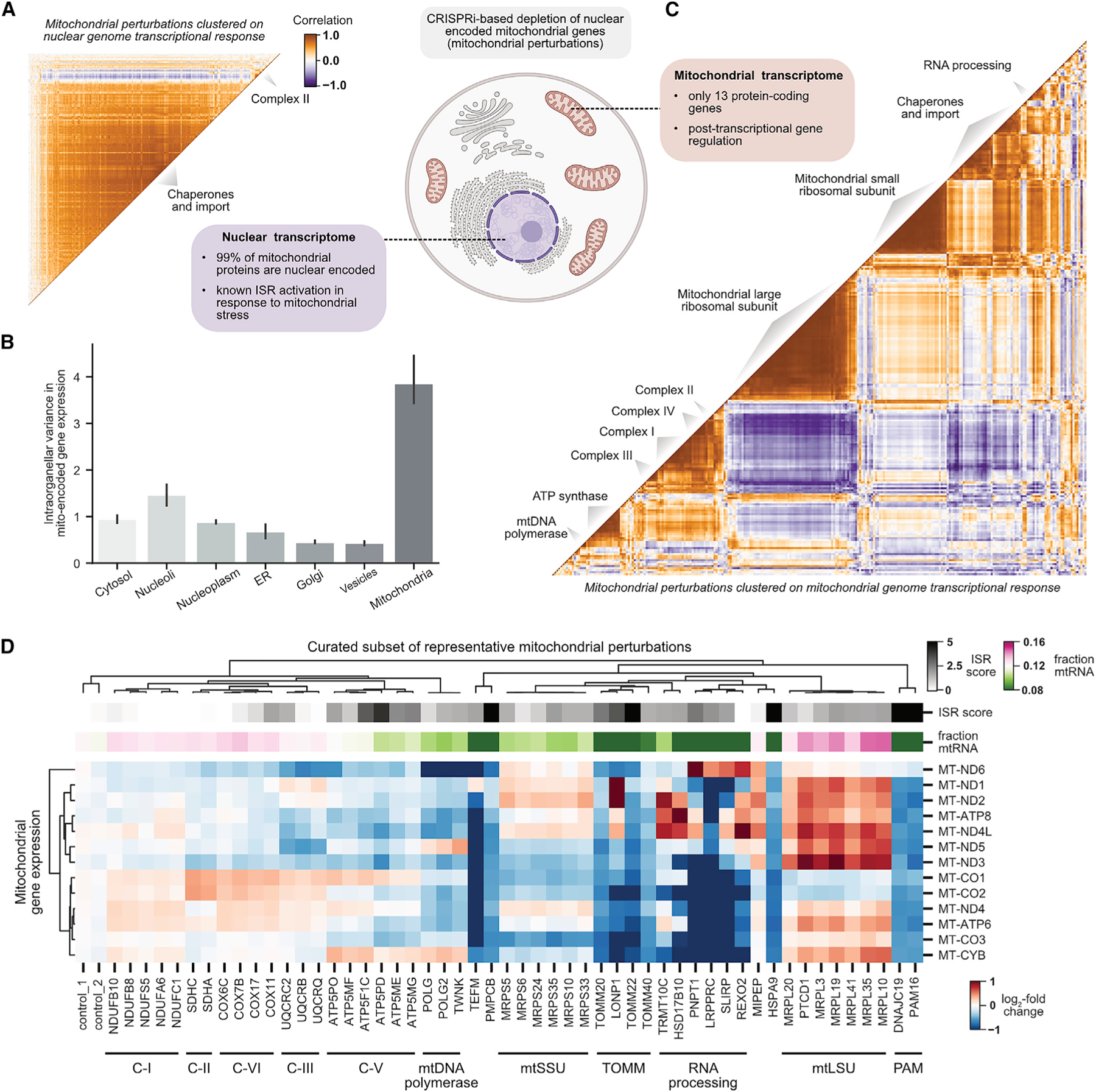
Global organization of the transcriptional response to mitochondrial stress (A) Clustering perturbations of nuclear-encoded genes whose protein products are targeted to mitochondria (mitochondrial perturbations) by nuclear transcriptional response. Mitochondrial perturbations were annotated by MitoCarta3.0 and subset to those with a strong transcriptional phenotype (n = 268). The heatmap displays the Pearson correlation between mean normalized gene expression profiles of mitochondrial perturbations in K562 cells clustered by HDBSCAN. (B) Variability in the mitochondrial transcriptome by perturbation localization. For each of the 13 mitochondrially encoded genes, the variance in mean normalized expression profiles was calculated between all perturbations with the same localization (in the Human Protein Atlas). Barplots represent the average across genes with 95% confidence interval obtained by bootstrapping. (C) Clustering mitochondrial perturbations by mitochondrial transcriptional response. Mitochondrial perturbations were defined as in (A). Gene expression profiles were restricted to the 13 mitochondrial-encoded genes. Heatmap is displayed and clustered as in (A). Clusters were manually annotated. (D) Heatmap visualizing the mitochondrial genome transcriptional response to diverse mitochondrial stressors. The expression of the 13 mitochondrially encoded genes (relative to controls) is shown for a subset of representative mitochondrial perturbations. See also Figure S8.

We began by comparing the nuclear transcriptional responses with CRISPRi-based depletion of nuclear-encoded mitochondrial genes (i.e., mitochondrial perturbations). Mitochondrial perturbations elicited relatively homogeneous nuclear transcriptional responses (Figures 6A and S8A). Although there was some variation in magnitude (e.g., proteostatic injury drove especially strong ISR activation), nuclear transcriptional responses generally did not discriminate perturbations by function, consistent with recent literature that has highlighted the role of the ISR in mitochondrial stress (Fessler et al., 2020; Guo et al., 2020; Mick et al., 2020; Münch and Harper, 2016; Quirós et al., 2017).

In contrast to the nuclear transcriptional response, the expression of mitochondrially encoded genes was highly variable between different mitochondrial perturbations (Figure 6B; Figures S8B–S8D). When we clustered mitochondrial perturbations based solely on expression of the 13 mitochondrially encoded genes, a pattern emerged: the clustering separated perturbations to complex I, complex IV, complex III, complex V, the mitochondrial large ribosomal subunit, the mitochondrial small ribosomal subunit, chaperones/import machinery, and RNA processing factors (Figure 6C; Figure S8E). In quantitative support of this observation, the mitochondrial transcriptome was far more predictive than the nuclear transcriptome in a random forest classifier trained to distinguish perturbations to different mitochondrial complexes (mitochondrial accuracy 0.64; nuclear accuracy: 0.25) (Figure S8F). We then visualized the expression signatures of a subset of representative perturbations (Figure 6D). The co-regulation of mitochondrial genes tended to reflect function, with the exception of the bicistronic mRNAs *ND4L/ND4* and *ATP8/ATP6* (Mercer et al., 2011). Although previous studies have described distinct regulation of the mitochondrial genome in response to specific perturbations (Richter-Dennerlein et al., 2016; Salvatori et al., 2020), our data generalize this phenomenon to a comprehensive set of stressors.

**Figure 7.**
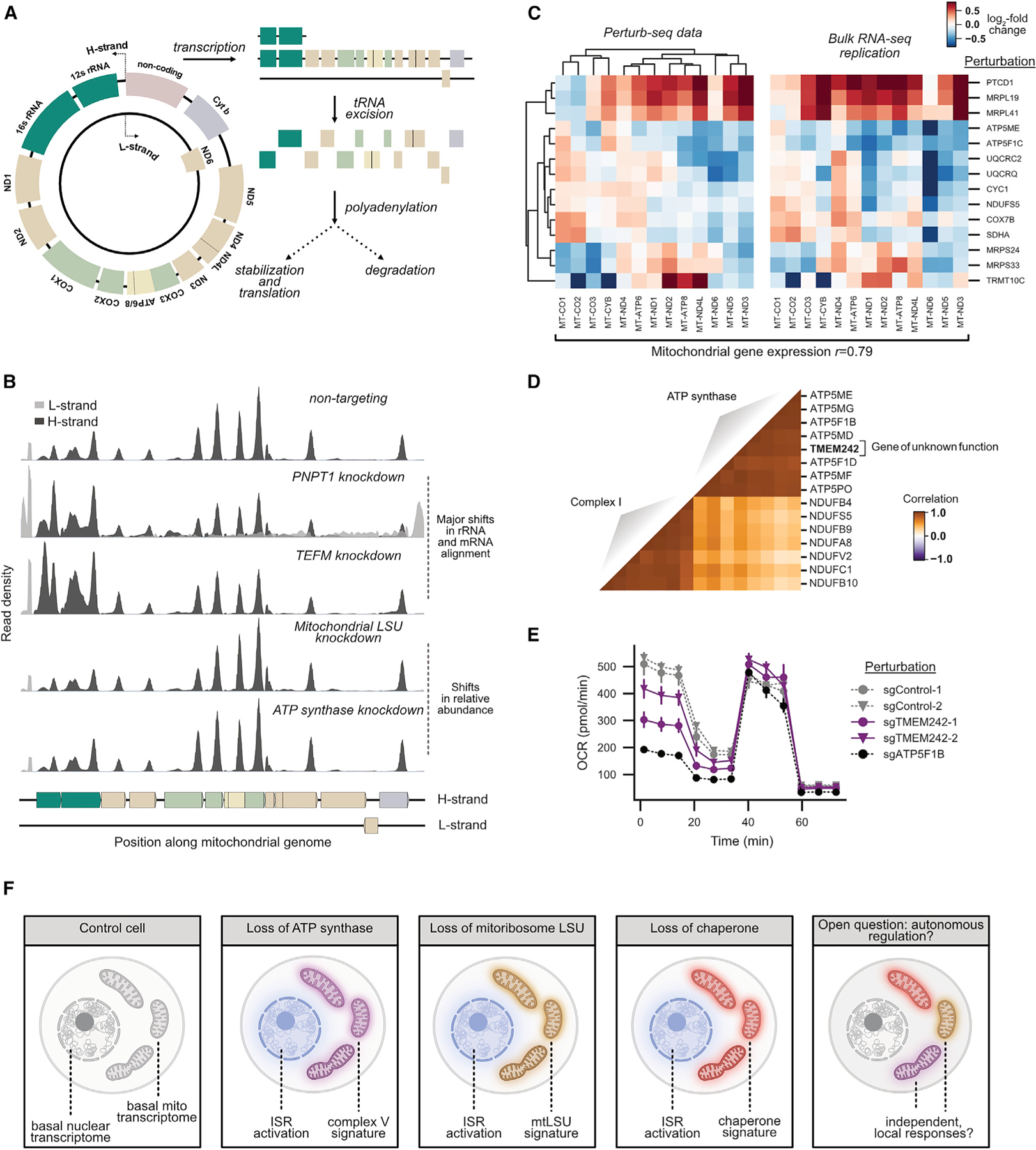
Investigating regulation of the mitochondrial genome in stress (A) Mitochondrial transcriptome schematic. (B) Density of Perturb-seq reads along the mitochondrial genome for select genetic perturbations. Reads are aligned to both the H-strand (dark gray) and L-strand (light gray). (C) Comparison of mitochondrial gene expression profiles between Perturb-seq and bulk RNA-seq. Heatmap displays changes in expression of the 13 mitochondria-encoded genes (columns) for perturbations (rows) in Perturb-seq and bulk total RNA-seq data collected from K562 cells. (D) Clustering of *TMEM242* genetic perturbation based on the mitochondrial transcriptome. Genetic perturbations to members of ATP synthase and complex I of the respiratory chain were compared with knockdown of *TMEM242*, a mitochondrial gene of unknown function. Gene expression profiles were restricted to the 13 mitochondrially encoded genes. The heatmap displays the Pearson correlation between pseudobulk *z*-normalized gene expression profiles of mitochondrial perturbations in K562 cells. (E) Effect of *TMEM242* knockdown on mitochondrial respiration. A Seahorse analyzer was used to monitor oxygen consumption rate (OCR) through a Mito stress test. Data are presented as average ± SEM, n = 6. (F) Schematic diagram of mitochondrial stress response. See also Figure S8.

Next, we wanted to shed light on the mechanistic basis for the complexity of mitochondrial genome responses. The mitochondrial genome is expressed by unique processes (Figure 7A; Kummer and Ban, 2021): mitochondrially encoded genes are transcribed as part of three polycistronic transcripts punctuated by tRNAs. These transcripts are then processed into rRNAs and mRNAs by tRNA excision, and individual mRNAs can be polyadenylated, translated, or degraded. This system limits the potential for transcriptional control but presents multiple opportunities for post-transcriptional regulation. To identify modes of perturbation-elicited differential expression, we examined the distribution of scRNA-seq reads along the mitochondrial genome (Figure 7B). To validate the utility of this position-based analysis, we confirmed that knockdown of known regulators of mitochondrial transcription (*TEFM*) and RNA degradation (*PNPT1*) led to major shifts in the position of reads along the mitochondrial genome. By contrast, many of the perturbations in our study appeared to cause shifts in the relative abundance of mRNAs rather than gross shifts in positional alignments. To determine whether the observed mitochondrial genome responses reflected regulation of the total level of mitochondrial mRNAs or specific regulation of mRNA polyadenylation, we performed bulk RNA sequencing without poly-A selection. We observed perturbation-specific changes in the level of total RNA similar to those measured by scRNA-seq (cophenetic correlation = 0.79; Figure 7C). Given the complexity of the observed responses, we propose that there are likely to be multiple mechanisms that impact the levels of the various mitochondrially encoded transcripts in response to different stressors.

Finally, we asked whether we could use the clustering produced by the mitochondrial genome to predict gene function. Knockdown of an unannotated gene, *TMEM242*, produced a signature resembling loss of ATP synthase (Figure 7D; Figure S8G). Supporting this relationship, the top five co-essential genes with *TMEM242* were components of ATP synthase in the Cancer Dependency Map, and in a Seahorse assay, basal respiration was decreased upon *TMEM242* knockdown (Figure 7E). Although this work was in progress, another group used a biochemical approach to show that *TMEM242* regulates ATP synthase complex assembly (Carroll et al., 2021). Together, these experiments highlight a novel factor required for ATP synthase activity.

## DISCUSSION

Single-cell CRISPR screens represent an emerging tool to generate rich genotype-phenotype maps. However, to date, their use has been limited to the study of preselected genes focused on predefined biological questions. Here, we perform genome-scale single-cell CRISPR screens and demonstrate how these screens enable data-driven dissection of a breadth of biological phenomena. Reflecting on this study, we highlight key insights and derive principles to guide future discoveries from rich genotype-phenotype maps.

A primary aim of large-scale functional screens is to organize genes into pathways or complexes. To this end, our Perturb-seq data recapitulated thousands of known relationships while also assigning new roles to genes involved in ribosome biogenesis, transcription, and respiration. However, other large-scale experimental techniques, such as protein-protein interaction mapping, genetic interaction mapping, and co-essentiality analysis, similarly group genes or proteins by function. How then are single-cell CRISPR screens distinct?

We argue that these screens are particularly powerful because of the intrinsic interpretability of comprehensive genotype-phenotype maps, enabling in-depth dissection of the functional consequences of genetic perturbations that impinge on many distinct aspects of cell biology. Of particular note is the ability to use the information-rich readouts to study complex, composite phenotypes, which are difficult to measure by other modalities. These composite phenotypes can be created in a data-driven (e.g., deriving transcriptional programs) or hypothesis-driven manner (e.g., measuring intron/exon ratios to study splicing), resulting in an enormous breadth of measured phenotypes. In the case of scRNA-seq, we show that it measures not only differential gene expression and the activity of critical transcriptional programs but also RNA splicing and processing, expression of TEs, differentiation, transcriptional heterogeneity, cell-cycle progression, and CIN. Once a phenotype is defined, the genotype-phenotype map can be used to explore its genetic underpinnings, in a manner analogous to a forward genetic screen, as well as its relationship to other cellular phenotypes.

An illustrative example of this process is our study of CIN. In a hypothesis-driven manner, we used our rich phenotypic data to discover a large collection of perturbations—which were only loosely connected by clustering on average transcriptional phenotypes—that promote CIN. Importantly, the single-cell nature of our data also allowed us to explore the relationship between karyotypic changes and other phenotypes, including cell-cycle progression and stress induction. Although aneuploidy is an important hallmark of cancer, it has been challenging to study with traditional genetic screens as it requires a single-cell, multivariate readout. In future work, this platform could be used to investigate interactions between genetic perturbations and specific karyotypes, karyotype-dependent stress responses, or the temporal evolution of karyotypes (Ben-David and Amon, 2020).

Because composite phenotypes can be generated and explored computationally without being preregistered at the time of data collection, rich genotype-phenotype maps provide a powerful resource for the discovery of new cellular behaviors. Using this ability, we discovered remarkable stress-specific changes in the expression of mitochondrially encoded transcripts. This discovery suggests a framework to explain how cells cope with diverse mitochondrial insults: a general nuclear response is layered over perturbation-specific changes in mitochondrial genome regulation (Figure 7F). Understanding how and in what contexts this regulation is adaptive may have important implications for diseases associated with mitochondrial stress. An intriguing additional question is whether individual mitochondria are able to regulate their expression autonomously. Combined with the nuanced responses observed here, this would support and extend the ‘‘co-location for redox regulation’’ (CoRR) hypothesis which holds that the mitochondrial genome has been retained through evolution to enable localized gene expression regulation (Allen, 2017).

A final theme emerging from our work is that single-cell CRISPR screens require only a fraction of the number of cells used by other approaches and thus are well suited to the study of iPSC-derived cells and *in vivo* samples. As technologies for single-cell, multimodal phenotyping advance, single-cell screens will continue to become more powerful. At present, the major limitation of single-cell CRISPR screens is cost. Careful experimental designs, such as multiplexed libraries, together with advances in single-cell phenotyping and DNA sequencing promise to greatly increase the scale of these experiments. To this point, we concluded our work by sequencing our genome-scale K562 libraries on a lower-cost, ultra-high throughput sequencing platform developed by Ultima Genomics, generating results equivalent to those sequenced on Illumina instruments (Data S1 [Figure ii]).

In sum, our study presents a blueprint for the construction and analysis of rich genotype-phenotype maps to serve as a driving force for the systematic exploration of genetic and cellular function. Our data are available in raw, processed, and interactive formats at http://gwps.wi.mit.edu.

### Limitations of the study

Technical aspects of our experimental design limit some conclusions of our study. (1) Perturb-seq is constrained by the cost of generating and sequencing scRNA-seq libraries. To minimize reagent use, we targeted most genes with a single library element, preventing comparison between independent sgRNAs. (2) We sampled a limited number of cells per perturbation. Greater cell numbers or sequencing depth would increase power. (3) Although 3′ scRNA-seq is an information-rich phenotype, other modalities including 5′ or full-length RNA-seq, protein-level readouts, or imaging have advantages for understanding certain processes. (4) We sampled at a limited number of time points and cell types. Sampling cells at a wider range of time points or cell types will undoubtedly uncover additional effects.

## STAR★METHODS

### RESOURCE AVAILABILITY

#### Lead contact

Further information and requests for resources and reagents should be directed to and will be fulfilled by the lead contact, Jonathan Weissman (weissman@wi.mit.edu).

#### Materials availability

Plasmids and CRISPRi sgRNA libraries generated in this study have been deposited to Addgene.

#### Data and code availability

- Raw sequencing data are deposited on SRA under BioProject PRJNA831566. key resources table An interactive data browser including processed, downloadable single-cell and pseudobulk populations is available at http://gwps.wi.mit.edu.
- Our codebase for Perturb-seq analysis is available at https://github.com/thomasmaxwellnorman/Perturbseq_GI and https://github.com/josephreplogle/guide_calling.
- Any additional information required to reanalyze the data reported in this paper is available from the lead contact upon request.

### EXPERIMENTAL MODEL AND SUBJECT DETAILS

#### Cell culture and lentiviral production

K562 cells were grown in RPMI-1640 with 25 mM HEPES, 2.0 g/l NaHCO3, and 0.3 g/l L-glutamine supplemented with 10% FBS, 2 mM glutamine, 100 units/ml penicillin, and 100 μg/ml streptomycin. hTERT-immortalized RPE1 cells (ATCC, CRL-4000) were grown in DMEM:F12 supplemented with 10% FBS, 0.01 mg/ml hygromycin B, 100 units/ml penicillin, and 100 μg/ml streptomycin. HEK293T cells were used for generation of lentivirus, and grown in DMEM supplemented with 10% FBS, 100 units/ml penicillin and 100 μg/ml streptomycin. Lentivirus was produced by co-transfecting HEK293T cells with transfer plasmids and standard packaging vectors using TransIT-LTI Transfection Reagent (Mirus, MIR 2306).

#### Cell line generation

CRISPRi K562 cells expressing dCas9-BFP-KRAB (KOX1-derived) were obtained from Gilbert et al. (2014). CRISPRi RPE1 cells expressing dCas9-BFP-KRAB (KOX1-derived) were obtained from Jost et al. (2017) and only used for growth screens. CRISPRi RPE1 cells were generated by stably transducing RPE1 cells (ATCC, CRL-4000) with lentivirus expressing ZIM3 KRAB-dCas9-P2A-BFP from a UCOE-SFFV promoter (pJB108) and sorting for BFP+ cells stably expressing the construct using fluorescence activated cell sorting. Cell lines were verified by monitoring BFP fluorescence over several generations to confirm stable integration and confirming knockdown of select surface markers by flow cytometry.

### METHOD DETAILS

#### Library design and cloning

A distinct set of genes was targeted for each of the three large-scale Perturb-seq experiments. For the K562 day 8 genome-scale experiment, we targeted (i) genes expressed in K562 cells (ii) transcription factors as detailed in Lambert et al. (2018) (iii) Cancer Dependency Map common essential genes as defined in 20Q1 (iv) non-targeting control sgRNAs accounting for 5% of the total library. To define expressed genes in K562 cells, we used a combination of bulk RNA-seq data from ENCODE (https://www.encodeproject.org/files/ENCFF717EVE/) and 10x Genomics 3’ single-cell RNA-seq data (https://www.ncbi.nlm.nih.gov/geo/query/acc.cgi?acc=GSE146194), selecting a set of genes accounting for ~99% of aligned reads in both datasets. For the K562 day 6 essential-scale experiment, we targeted (i) Cancer Dependency Map common essential genes as defined in 20Q1 (ii) non-targeting control sgRNAs accounting for 5% of the total library. For the RPE1 day 7 essential-scale experiment, we targeted (i) 20Q1 Cancer Dependency Map common essential genes (https://depmap.org/portal/download/) (ii) a number of hand-selected genes with interesting phenotypes in the K562 genome-wide Perturb-seq dataset (iii) non-targeting control sgRNAs accounting for 5% of the total library. To define control perturbations, we randomly sampled non-targeting control perturbations from Horlbeck et al. (2016). A small number of genes were lost in this pipeline due to changes in gene annotation between datasets.

To minimize library size while maximizing knockdown, multiplexed CRISPRi libraries were constructed which targeted each gene with two unique sgRNAs expressed from tandem U6 expression cassettes in a single lentiviral vector, as previously described in Replogle et al. (2020). The Horlbeck et al. (2016) CRISPRi sgRNA libraries were used as a source of sgRNAs targeting each gene, with the optimal sgRNA pair targeting each gene selected based on a balance of empirical data with computational predictions. For strong essential genes (defined by a p-value<0.001 and gamma<-0.2 in the Horlbeck et al. [2016] CRISPRi growth screen), sgRNAs were ranked by growth. Then, for genes that produced a significant phenotype in previous CRISPRi screens, sgRNAs were ranked by a discriminant score multiplying the negative log_10_ p-value by the effect size. Finally, for genes without any empirical evidence, sgRNAs were ranked according to the Horlbeck et al. (2016) hCRISPRi v2.1 algorithm. The full sgRNA content of the K562 day 8 genome-scale library, K562 day 6 essential-wide library, and RPE1 day 7 essential-wide library can be found in Table S1.

We adapted the protocol previously described in Replogle et al. (2020) to clone libraries with capture sequences for 3’ direct capture Perturb-seq. Briefly, an sgRNA lentiviral expression vector (pRS275/pJR101) was derived from the parental pJR85 (Addgene #140095), modified to incorporate a GFP fluorescent marker to avoid spectral overlap with BFP+ CRISPRi constructs and a UCOE element upstream of the EF1alpha promoter to prevent silencing (Table S4). A two-step restriction enzyme digestion and ligation cloning of oligos into pRS275/pJR101 was performed to maintain coupling of sgRNAs targeting the same gene. Oligos encoding the targeting regions of dual-sgRNA pairs were synthesized as an oligonucleotide pool (Twist Bioscences) with the structure: 5’- PCR adapter - CCACCTTGTTG – targeting region A - gtttcagagcgagacgtgcctgcaggatacgtctcagaaacatg – targeting region B - GTTTAAGAGCTAAGCTG - PCR adapter-3’. When ordering oligos, the representation of essential genes was increased to compensate for growth phenotypes (see below). Oligo pools were amplified, digested with BstXI/BlpI, and ligated into pRS275/pJR101. To add an sgRNA constant region and U6 promoter to the vector, pJR89 (Addgene #140096) was BsmBI-digested and ligated into the intermediate library.

#### K562 and RPE1 growth screens

Pooled sgRNA growth screens in K562 cells were used to quantify growth phenotypes of sgRNA pairs targeting expressed genes. CRISPRi K562 cells expressing dCas9-BFP-KRAB were transduced with lentiviral particles encoding the dual-sgRNA library by spinfection (1000g) with polybrene (8 ug/ml) to obtain an infection rate of~25%-35%. Screens were performed in biological replicate with the aim of maintaining 1000 cells per library element for the duration of the screen. Between day 2 and day 6 post-transduction, cells were selected for lentiviral infection using 1 ug/mL puromycin, replenished every 24 hours. On day 7 post-transduction, an aliquot of cells was harvested as an initial time point) The rest of the cell population was passaged for 10 more days and collected at final time point.

Pooled sgRNA growth screens in RPE1 cells were used to quantify growth phenotypes of sgRNA pairs targeting common essential genes. The CRISPRi RPE1 cell line expressing dCas9-BFP-KRAB was used for growth screens which took place before the publication of the next-generation ZIM3 KRAB domain. Cells were transduced in biological replicate with lentiviral particles encoding the dual-sgRNA library by replating cells into virus-laden media with polybrene (8 ug/ml) to obtain an infection rate of ~45%. Because RPE1 cells are puromycin resistant, we performed the screen without selection for sgRNA-infected cells, nonetheless maintaining an infection rate-corrected 1000 cells per library element for the duration of the screen. On day 6 post-transduction, an aliquot of cells was harvested as an final time point for direct comparison to the abundances in the plasmid library.

For both K562 and RPE1 growth screens, DNA libraries of the initial and final samples were prepared for deep sequencing by genomic DNA isolation and PCR amplification of dual-sgRNA amplicons as described previously (Nuñez et al., 2021; Replogle et al., 2020). First, a NucleoSpin Blood XL kit (Macherey–Nagel) was used to extract genomic DNA (gDNA) from cells. Then, isolated gDNA or plasmid DNA was amplified by 22 cycles (gDNA) or 13 cycles (plasmid DNA) of PCR using NEBNext Ultra II Q5 PCR MasterMix (NEB), appending Illumina adaptors and sample indices (oJR234 forward primer: 5’-AATGATACGGCGACCACCGAGATCTACACCGCGGTCTGTA TCCCTTGGAGAACCACCT-3’; index primers 5’-CAAGCAGAAGACGGCATACGAGATnnnnnGCGGCCGGCTGTTTCCA GCTTAGCTCTTAAA-3’). Amplicons were isolated by a 0.5–0.65X SPRI bead selection (SPRIselect Beckman Coulter #B23318). Sequencing was performed on a NovaSeq 6000 (Illumina) using a 19 bp read 1, 19 bp read 2, and 5 bp index read 1 with custom sequencing primers oJR326 (custom read 1, 5’- CGCGGTCTGTATCCCTTGGAGAACCACCTTGTTGG-3’), oJR328 (custom read 2, 5’- GCGGCCGGC TGTTTCCAGCTTAGCTCTTAAAC-3’), and oJR327 (custom index read 1, 5’- GTTTAAGAGCTAAGCTGGAAACAGCCGGCCGC-3’).

#### Perturb-seq experiments

The selection of time points for our experiments is based on a combination of previously published CRISPR screens, Perturb-seq experiments, and the goals of our experiment. The constraints on the design of CRISPR growth screens differ significantly from Perturb-seq experiments. In growth screens, an amplification of signal occurs over time as cells drop out of the population, so experiments often compare representation between an early time point (~3–5 days post-transduction) and a much later final timepoint (~14–28 days post-transduction). In contrast, in Perturb-seq screens, the phenotype is measured directly from the perturbed cells that are sampled on the day of scRNA-seq. Relatively earlier timepoints may then be advantageous because:

- libraries remain more balanced, especially when studying perturbations targeting essential genes that are prone to dropping out over time at the representation levels typically used in scRNA-seq experiments; and
- more ‘‘direct’’ phenotypic consequences of the genetic perturbation are observed (*i.e.*, the transcriptomes reflect the cellular response to perturbation rather than later indirect consequences like cell death).

In contrast, the possible advantages of sampling cells at later timepoints are:

- time is required to allow for CRISPR machinery to be expressed, the genetic perturbation to occur (in this case CRISPRi), and finally protein depletion to occur; and
- for some perturbations, longer time points might be required in order to observe a phenotype (*e.g.*, perturbations that result in buildup of cellular metabolites).

In designing our experiment, we wanted to ensure that we would sample the phenotypic consequences of perturbing essential genes which would quickly deplete from our library. We thus chose to sample at two time points in K562 cells as the phenotypic effects of different genetic perturbations can manifest at variable time points based on technical and biological factors. While each gene depletes and causes a cellular phenotype based on unique characteristics, the majority of genes are widely accepted to have phenotypes in between approximately day 6 to day 8 of the screen.

To perform our K562 day 8 genome-scale Perturb-seq experiment, library lentivirus was packaged into lentivirus in 293T cells and empirically measured in K562 cells to obtain viral titers. CRISPRi K562 cells were transduced via spinfection (1000g) with polybrene (8 ug/ml) with the target of obtaining an infection rate of ~10%. Cells were maintained at a viability of >90%, a coverage of 1000 cells per library element, and a density of 250,000 to 1,000,000 cells/ml for the course of the experiment. Three days post-transduction, an infection rate of 14% was measured, and cells were sorted to near purity by FACS (FACSAria2, BD Biosciences), using GFP as a marker for sgRNA vector transduction. Eight days post infection, the cells were measured to be 97% GFP+ (LSR2, BD Biosciences), >90% viable, and at a concentration of ~800,000 cells/ml (Countess II, ThermoFisher). Cells were prepared for single-cell RNA-sequencing by resuspension in 1X PBS with 0.04% BSA as detailed in the 10x Genomics Single Cell Protocols Cell Preparation Guide (10x Genomics, CG00053 Rev C). Cells were then separated into droplet emulsions using the Chromium Controller (10x Genomics) with Chromium Single-Cell 3° Gel Beads v3 (10x Genomics, PN-1000075 and PN-1000153) across 273 ‘‘lanes’’/’’GEM groups’’ following the 10x Genomics Chromium Single Cell 3ʹ Reagent Kits v3 User Guide with Feature Barcode technology for CRISPR Screening (CG000184 Rev C) with the goal of recovering ~15,000 cells per GEM group before filtering. Because the formation of droplet emulsions occurred in batches of 8 GEM groups over several hours, fresh populations of cells were obtained every hour to prevent alterations in single-cell transcriptomes.

To perform our K562 day 6 essential-scale Perturb-seq experiment, library lentivirus was packaged into lentivirus in 293T cells and empirically measured in K562 cells to obtain viral titers. CRISPRi K562 cells were transduced via spinfection (1000g) with polybrene (8 ug/ml) with the target of obtaining an infection rate of ~10% with maintenance of cells as described above. Three days post-transduction, an infection rate of 15% was measured, and cells were sorted to near purity by FACS (FACSAria2, BD Biosciences), using GFP as a marker for sgRNA vector transduction. Six days post infection, the cells were measured to be 93% GFP+ (LSR2, BD Biosciences), >90% viable, and at a concentration of ~600,000 cells/ml (Countess II, ThermoFisher). Cells were prepared for single-cell RNA-sequencing by resuspension in 1X PBS with 0.04% BSA as detailed in the 10x Genomics Single Cell Protocols Cell Preparation Guide (10x Genomics, CG00053 Rev C). Cells were then separated into droplet emulsions using the Chromium Controller (10x Genomics) with Chromium Single-Cell 3′ Gel Beads v3 (10x Genomics, PN-1000075 and PN-1000153) across 48 ‘‘lanes’’/’’GEM groups’’ following the 10x Genomics Chromium Single Cell 3ʹ Reagent Kits v3 User Guide with Feature Barcode technology for CRISPR Screening (CG000184 Rev C) with the goal of recovering ~15,000 cells per GEM group before filtering.

To perform our RPE1 day 7 essential-scale Perturb-seq experiment, library lentivirus was packaged into lentivirus in 293T cells and empirically measured in RPE1 cells to obtain viral titers. CRISPRi RPE1 cells expressing ZIM3 KRAB-dCas9-P2A-BFP were transduced via replating into virus-laden media with polybrene (8 ug/ml) with the target of obtaining an infection rate of ~10%. Three days post-transduction, an infection rate of 7% was measured, and cells were sorted to near purity by FACS (FACSAria2, BD Biosciences), using GFP as a marker for sgRNA vector transduction. Seven days post infection, the cells were measured to be 86% GFP+ (LSR2, BD Biosciences) and >95% viable (Countess II, ThermoFisher). After trypsin dissociation, cells were prepared for single-cell RNA-sequencing by resuspension in 1X PBS with 0.04% BSA as detailed in the 10x Genomics Single Cell Protocols Cell Preparation Guide (10x Genomics, CG00053 Rev C). Cells were then separated into droplet emulsions using the Chromium Controller (10x Genomics) with Chromium Single-Cell 3′ Gel Beads v3 (10x Genomics, PN-1000075 and PN-1000153) across 56 ‘‘lanes’’/’’GEM groups’’ following the 10x Genomics Chromium Single Cell 3ʹ Reagent Kits v3 User Guide with Feature Barcode technology for CRISPR Screening (CG000184 Rev C) with the goal of recovering ~15,000 cells per GEM group before filtering.

#### Perturb-seq library preparation and sequencing

For preparation of gene expression and sgRNA libraries, samples were processed according to 10x Genomics Chromium Single Cell 3ʹ Reagent Kits v3 User Guide with Feature Barcode technology for CRISPR Screening (CG000184 Rev C). To allow for parallel library preparation, samples were arranged in 96-well plates with magnetic selections conducted on an Alpaqua Catalyst 96 plate (#A000550). For sequencing, mRNA and sgRNA libraries were pooled to avoid index collisions at a 10:1 ratio. Libraries were sequenced on both (i) a NovaSeq 6000 (Illumina) according to the 10x Genomics User Guide and (ii) the Ultima Genomics ultra-high throughput sequencing platform.

For sequencing on the Ultima Genomics (UG) platform, final 10x libraries were converted using conversion primers that anneal to the R1 and R2 regions of the 10X library and contain a UG-specific adapter sequence overhang and sample index. 8 PCR cycles were used for conversion. Converted libraries were bead purified and quantified. After pooling libraries, pools were seeded and clonally amplified on UG sequencing beads and sequenced on a UG prototype Sequencer. Single reads were generated from the 10x 3’ libraries, reading the 10X cell barcode, unique molecular identifier (UMI), and 3’ end of the cDNA transcript. A specific sequencing protocol including a high volume of dT nucleotides was used to accommodate the high nucleotide consumption in the poly (dT) stretch of the cDNA. Following sequencing, the single reads were quality-trimmed and split into two sub-sequences corresponding to Read1 (10X cell barcode and UMI) and Read2 (cDNA), which were used as input to Cell Ranger for alignment.

#### rRNA analyses

K562s expressing Zim3-dCas9-2A-BFP were spinfected in biological duplicate (targeting sgRNAs) or quadruplicate (non-targeting sgRNAs) with lentivirus expressing GFP and an sgRNA. Two days after spinfection, the cells were sorted for GFP+ on a BD ARIA II. Sort purity was generally >95%. After the sort, cells were maintained in media supplemented with 4 ug/ml puromycin for four days and then recovered for two days. Cells were counted, collected by centrifugation, and harvested by vigorous vortexing in Tri Reagent (ThermoFisher AM9738).

RNA was extracted with chloroform according to the manufacturer’s instructions, quantified by nanodrop, and snap frozen. Small samples were diluted to 200 ng/ul and run on Bioanalyzer RNA nano chips (Agilent 5067-1511) according to the manufacturer’s instructions. Runs were aligned to the 18s peak and signal intensity was normalized to total RNA area.

#### Integrator co-depletion

K562s expressing Zim3-dCas9-2A-BFP were spinfected with lentivirus expressing GFP and an sgRNA. Two days after spinfection, the cells were sorted for GFP+ on a BD ARIA II. Sort purity was generally >95%. After the sort, cells were maintained in media supplemented with 4 ug/ml puromycin for four days and then recovered for two days. Cells were counted, washed twice with DPBS, and collected as pellets. The pellets were resuspended in SDS lysis buffer (100 mM Tris pH 8.0, 1% SDS), thermomixed at 95°/1500 RPM for thirty minutes, aliquoted, and snap-frozen.

##### Quantification for western blots

An equal amount of material was loaded, as assessed by lysate A280.

#### Integrator co-immunoprecipitation

Human expression plasmids encoding codon-optimized INTS10 or His8-INTS10 were synthesized (Twist Bioscience) and transfected into HEK 293T/17 cells (ATCC CRL-11268) with FuGene HD (Promega E2311) according to the manufacturer’s protocol. Two days later, the cells were washed twice with DPBS and harvested with IP lysis buffer (25 mM Tris-HCl pH 7.4, 150 mM NaCl, 1 mM EDTA, 1% NP-40, 5% glycerol; ThermoFisher 87787) supplemented with protease inhibitors (ThermoFisher A32965). Lysates were nutated at 4° for 30 mins, clarified by centrifugation at 12,000xg for 10 minutes, and snap-frozen. Concentrations were measured with the BCA assay (ThermoFisher 23225).

Lysates were thawed on ice, supplemented with imidazole to 10 mM, and nutated at 4° for 30 minutes with cobalt magnetic beads (ThermoFisher 10103D) pre-equilibrated in IP lysis buffer + 10 mM imidazole. The beads were separated on a magnet, washed twice with lysis buffer + 10 mM imidazole, and eluted with lysis buffer + 300 mM imidazole.

##### Quantification for western blots

For input samples, an equal amount of material was loaded, as assessed by BCA. For IP samples, an equal volume of eluate was loaded.

#### Integrator purification

Human expression plasmids encoding codon-optimized HIS-INTS10, INTS13, INTS14, and C7orf26 were synthesized (Twist Bioscience) and co-transfected with ExpiFectamine 293 (ThermoFisher A14524) into Expi293 cells (ThermoFisher A14527) maintained in Expi293 medium (ThermoFisher A1435101) according to the manufacturer’s instructions. The cells were harvested after four days and snap frozen.

The pellets were resuspended in CHAPS Lysis Buffer (50 mM HEPES pH 8.0, 300 mM NaCl, 0.2% CHAPS, 10% glycerol, 1 mM TCEP, 1 mM EDTA, 0.5 mM PMSF, 1x protease inhibitors, 0.002% benzonase) and stirred at 4° for 30 minutes. The lysates were clarified at 120,000xg for 30 minutes, supplemented with 15 mM imidazole, and nutated for an hour with Ni-NTA agarose beads (ThermoFisher 25215) pre-equilibrated in CHAPS lysis buffer + 15 mM imidazole. The beads were loaded into a gravity column, washed with >10 volumes of wash buffer (50 mM HEPES pH 8.0, 300 mM NaCl, 10% glycerol, 1 mM TCEP, 1 mM EDTA, 15 mM imidazole), and eluted with wash buffer supplemented with 250 mM imidazole. The eluate was concentrated and buffer exchanged into SEC buffer (50 mM HEPES pH 8.0, 150 mM KCl, 10% glycerol, 1 mM EDTA) by ultrafiltration, and snap frozen.

The eluate was thawed on ice, passed through a 0.2 μM PES filter, and loaded onto an Superdex 200 Increase 10/300 GL column pre-equilibrated with SEC buffer. Fractions were collected and flash frozen.

##### Quantification for gels and western blots

An equal volume of sample from each SEC fraction was loaded. Less Ni-NTA eluate was loaded to account for the dilution over SEC.

#### Drosophila Integrator biochemistry

##### Stable cell lines and nuclear extract preparation

Relevant *Drosophila* cDNAs were cloned into a pMT-3xFLAG-puro plasmid (Elrod et al., 2019; Huang et al., 2020) following the metallothionein promotor and 3x-FLAG tag. 2x10^6^ Drosophila DL1 cells were plated in Schneider’s media supplemented with 10% FBS in a 6-well plate overnight and 2 μg of plasmid was transfected using Fugene HD (Promega, Madison WI, #E2311). Plasmid DNA was mixed with 8 μL Fugene and 100 μL media and incubated at room temperature for 15 minutes before being added to cells. After 24 hours, 2.5 μg/mL puromycin was added to the media to select and maintain the cell population. Cells were transitioned to SFX media without serum for large scale growth. Protein expression for nuclear extract was induced by adding 500 mM copper sulfate for 48 hours to 1 liter of each cell line grown to approximately 1x10^7^ cells/mL.

Cells were collected and washed in cold PBS and then pelleted by centrifugation. Cells were then resuspended in five times the cell pellet volume of Buffer A (10mM Tris pH8, 1.5 mM MgCl_2_, 10 mM KCl, 0.5mM DTT, and 0.2mM PMSF). Resuspended cells were allowed to swell during a 15-minute rotation at 4°C. After pelleting down at 1,000g for 10 minutes, two volumes of the original cell pellet of Buffer A were added and cells were homogenized with a dounce pestle B for 20 strokes on ice. Nuclear and cytosolic fractions were then separated by centrifugation at 2,000g for 10 minutes. To attain a nuclear fraction, the pellet was washed once with Buffer A before resuspending in an equal amount of the original cell pellet volume of Buffer C (20 mM Tris pH8, 420mM NaCl, 1.5 mM MgCl_2_, 25% glycerol, 0.2 mM EDTA, 0.5 mM PMSF, and 0.5 mM DTT). The sample was then homogenized with a dounce pestle B for 20 strokes on ice and rotated for 30 minutes at 4°C before centrifuging at 15,000g for 30 minutes at 4°C. Finally, supernatants were collected and subjected to dialysis in Buffer D (20 mM HEPES, 100 mM KCl, 0.2 mM EDTA, 0.5 mM DTT, and 20% glycerol) overnight at 4°C. Prior to any downstream applications, nuclear extracts were centrifuged again at 15,000g for 3 minutes at 4°C to remove any precipitate.

##### Anti-FLAG affinity purification and western blotting

To purify FLAG-tagged Integrator complexes for mass spectrometry, generally between 8 and 10 mg of DL1 nuclear extract (approximately 1.9 mL of extract depending on the concentration) was mixed with 100 μL anti-Flag M2 affinity agarose slurry (Sigma-Aldrich, #A2220) washed with 0.1 M glycine then equilibrated in binding buffer (20 mM HEPES pH7.4, 150 mM KCl, 10% Glycerol, 0.1% NP-40). This mixture was rotated for four hours at 4°C. Following the four-hour incubation/rotation, five sequential washes were carried out in binding buffer with a 10-minute rotation at 4°C followed by a 1,000g centrifugation at 4°C. After a final wash with 20 mM HEPES buffer, the supernatant was removed using a pipette and the beads were kept cold and submitted to the mass spectrometry core where the protein complexes were eluted by digestion (described below). For immunoprecipitation samples intended for western blot, a similar protocol was used. 25 μL of bead slurry and 200 μL of extract sample were rotated for two hours at 4°C. After the fifth wash with binding buffer, protein complexes were eluted from the anti-FLAG resin by adding 50 μL of 2X SDS loading buffer and boiled at 95°C for five minutes. For western blots, input samples were generated by adding equal volume of 2X SDS loading buffer to nuclear extract and 1/10 of the immunoprecipitation was loaded as estimated by protein mass. Total protein was resolved on SDS polyacrylamide gels (Bio-Rad) with DTT, followed by transfer onto polyvinylidene difluoride (PVDF) membranes (ThermoFisher). Blots were probed as previously described using *Drosophila*-specific antibodies raised against recombinant GST fusion proteins expressed in E. coli (Huang et al., 2020).

##### Mass spectrometry sample digestion

The samples were prepared in a similar manner as described previously (Anderson et al., 2020). Briefly, the agarose bead-bound proteins were washed several times with 50 mM Triethylammonium bicarbonate (TEAB) pH 7.1, before being solubilized with 40 μL of 5% SDS, 50 mM TEAB, pH 7.55 followed by a room temperature incubation for 30 minutes. The supernatant containing the proteins of interest was then transferred to a new tube, reduced by making the solution 10 mM Tris(2-carboxyethyl)phosphine (TCEP) (Thermo, #77720), and further incubated at 65°C for 10 minutes. The sample was then cooled to room temperature and 1 μL of 1M iodoacetamide acid was added and allowed to react for 20 minutes in the dark. Then, 5 μL of 12% phosphoric acid was added to the 50 μL protein solution followed by 350 μL of binding buffer (90% Methanol, 100 mM TEAB final; pH 7.1). The resulting solution was administered to an S-Trap spin column (Protifi, Farmingdale NY) and passed through the column using a bench top centrifuge (30 second spin at 4,000g). The spin column was then washed three times with 400 μL of binding buffer and centrifuged (1200 rpm, 1 min). Trypsin (Promega, #V5280) was then added to the protein mixture in a ratio of 1:25 in 50 mM TEAB, pH=8, and incubated at 37°C for 4 hours. Peptides were eluted with 80 μL of 50 mM TEAB, followed by 80 μL of 0.2% formic acid, and finally 80 μL of 50% acetonitrile, 0.2% formic acid. The combined peptide solution was then dried in a speed vacuum (room temperature, 1.5 hours) and resuspended in 2% acetonitrile, 0.1% formic acid, 97.9% water and aliquoted into an autosampler vial.

##### NanoLC MS/MS Analysis

Peptide mixtures were analyzed by nanoflow liquid chromatography-tandem mass spectrometry (nanoLC-MS/MS) using a nano-LC chromatography system (UltiMate 3000 RSLCnano, Dionex, Thermo Fisher Scientific, San Jose, CA). The nano-LC-MS/MS system was coupled on-line to a Thermo Orbitrap Fusion mass spectrometer (Thermo Fisher Scientific, San Jose, CA) through a nanospray ion source (Thermo Scientific). A trap and elute method was used to desalt and concentrate the sample, while preserving the analytical column. The trap column (Thermo Scientific) was a C18 PepMap100 (300 μm X 5 mm, 5 μm particle size) while the analytical column was an Acclaim PepMap 100 (75 mm X 25 cm) (Thermo Scientific). After equilibrating the column in 98% solvent A (0.1% formic acid in water) and 2% solvent B (0.1% formic acid in acetonitrile (ACN)), the samples (2 μL in solvent A) were injected onto the trap column and subsequently eluted (400 nL/min) by gradient elution onto the C18 column as follows: isocratic at 2% B, 0–5 min; 2% to 32% B, 5–39 min; 32% to 70% B, 39–49 min; 70% to 90% B, 49–50 min; isocratic at 90% B, 50–54 min; 90% to 2%, 54–55 min; and isocratic at 2% B, until the 65 minute mark.

All LC-MS/MS data were acquired using XCalibur, version 2.1.0 (Thermo Fisher Scientific) in positive ion mode using a top speed data-dependent acquisition (DDA) method with a 3 second cycle time. The survey scans (m/z 350-1500) were acquired in the Orbitrap at 120,000 resolution (at m/z = 400) in profile mode, with a maximum injection time of 100 m s and an AGC target of 400,000 ions. The S-lens RF level was set to 60. Isolation was performed in the quadrupole with a 1.6 Da isolation window, and CID MS/MS acquisition was performed in profile mode using rapid scan rate with detection in the ion-trap using the following settings: parent threshold = 5,000; collision energy = 32%; maximum injection time 56 msec; AGC target 500,000 ions. Monoisotopic precursor selection (MIPS) and charge state filtering were on, with charge states 2–6 included. Dynamic exclusion was used to remove selected precursor ions, with a +/− 10 ppm mass tolerance, for 15 seconds after acquisition of one MS/MS spectrum.

##### Database Searching

Tandem mass spectra were extracted and charge state deconvoluted using Proteome Discoverer (Thermo Fisher, version 2.2.0388). Deisotoping was not performed. All MS/MS spectra were searched against the Uniprot *Drosophila* database (version 04-04-2018), using Sequest. Searches were performed with a parent ion tolerance of 5 ppm and a fragment ion tolerance of 0.60 Da. Trypsin was specified as the enzyme, allowing for two missed cleavages. Fixed modification of carbamidomethyl (C) and variable modifications of oxidation (M) and deamidation were specified in Sequest. Heatmaps in Figure S9A were made using Morpheus from the Broad Institute, https://software.broadinstitute.org/morpheus. Volcano plots in Figure S9B were generated using average number of peptide counts quantified using mass spectrometry for three independent measurements of purifications, which also was the basis for adjusted p-values. These values were all calculated and plotted using GraphPad Prism software.

#### Integrator PRO-seq

Pro-seq was conducted largely according to published protocols with slight modifications (Reimer et al., 2021). K562s expressing dCas9-BFP-KRAB were spinfected with lentivirus expressing GFP and an sgRNA. Two days after spinfection, the cells were sorted for GFP+ on a BD ARIA II. Sort purity was generally >95%. After the sort, cells were maintained in media supplemented with 4 ug/ml puromycin for three days and then recovered for two days.

Cells were counted, harvested by centrifugation, and washed with cold DPBS. All subsequent steps took place at 4° or on ice. All solutions were made with RNase-free reagents and were 0.2 μm filtered and chilled before use. 12 million cells were pelleted by centrifugation, resuspended in 250ul of buffer W (10 mM Tris pH 8.0, 10 mM KCl, 250 mM sucrose, 5 mM MgCl2, 1 mM EGTA, 0.5 mM DTT, 10% glycerol, 1x protease inhibitor [ThermoFisher A32965], and 0.02% v/v SUPERase-In RNase inhibitor [AM2694], strained, and transferred to conical tubes that had been coated with 1% BSA in PBS overnight. The cells were permeabilized by dilution in 10 ml of buffer P (buffer W + 0.1% v/v Igepal CA-630 + 0.05% v/v Tween-20) and incubated for 5 minutes. The permeabilized cells were harvested by centrifugation at 400xg for 5 minutes, resuspended in 10 mL of buffer W, harvested by centrifugation at 400xg for 5 minutes, and resuspended in 250 ul buffer F (50 mM Tris pH 8.0, 40% v/v glycerol, 5 mM MgCl2, 1.1 mM EDTA, 0.5 mM DTT, 0.02% v/v SUPERase-In RNase inhibitor). ≥97% permeabilization efficiency was confirmed on a NucleoCounter NC-202 and permeabilized cells were snap frozen.

PRO-seq libraries were generated and sequenced by the Nascent Transcriptomics Core at Harvard Medical School according to their standard protocol. PRO-seq data were aligned and quantified using STAR (version 2.7.9a) with parameters alignEndsType=Local, outFilterMultimapNmax=20, outFilterScoreMinOverLread=0.3, and outFilterMatchNminOverLread=0.3. For comparison of gene-level expression profiles, gene counts were normalized for sequencing depth (reads per million), log-transformed, and subset to well-expressed genes (n=758 genes with >3000 rpm). Then, Spearman’s correlation was used to compare the similarity of expression profiles.

#### SDS-PAGE and western blotting

Samples were mixed with sample loading buffer (Licor 928-40004) supplemented with DTT and incubated at 95° for 5 minutes. SDS-PAGE was performed with pre-cast 4–12% gradient gels (ThermoFisher NW04127BOX) in MOPS (ThermoFisher B000102) according to the manufacturer’s instructions.

For Coomassie staining, gels were washed thoroughly in water, incubated with ReadyBlue Protein Gel Stain (Sigma RSB-1L) overnight, and destained in water. For western blots, proteins were transferred to nitrocellulose membranes by semi-dry transfer (Biorad 1704158) according to the manufacturer’s instructions. Membranes were rinsed in water and stained with Revert 700 Total Protein Stain according to the manufacturer’s instructions. The membranes were then rinsed in TBS, rocked with Everyblot Blocking Buffer (Biorad 12010020) at room temperature for > 30 minutes, and rocked with primary antibody overnight at 4°. The membranes were washed with TBST, and rocked with IR800CW-labeled secondary antibodies for 30–60 minutes, washed with TBST, and imaged on a Licor Odyssey CLx.

#### CD11b cell surface staining

K562s expressing dCas9-BFP-KRAB were co-spinfected with lentiviruses expressing GFP-sgKDM1A and mCherry-sgPTPN1. Eight days after spinfection, the cells were counted and harvested by centrifugation. Cells were washed with PBE buffer (DPBS + 0.5% BSA + 2 mM EDTA) and resuspended with α-CD11b-AF647 antibody diluted 1:50 in PBE. Cells were incubated at 4° in the dark for 30 minutes, washed twice with PBE, and analyzed on a BD LSRFortessa. The populations were gated from a single sample as sgKDM1A (GFP+,mCherry-), sgPTPN1 (GFP-, mCherry+), and sgKDM1A/sgPTPN1 (GFP+, mCherry+). Unstained K562s expressing either GFP or mCherry were used as single color compensation controls. AF647 was compensated with UltraComp eBeads Plus (Thermo 01-3333-42) labeled with α-CD11b-AF647.

#### Internally controlled growth assays

K562s expressing Zim3-dCas9-2A-BFP were co-spinfected in triplicate with lentiviruses expressing GFP-sgKDM1A and mCherry-sgPTPN1, or with lentivirus expressing GFP and a non-targeting sgRNA. Every two days, cells were analyzed for BFP, GFP, and mCherry on an Attune flow cytometer. Enrichment was calculated as sgKDM1A (BFP+, GFP+, mCherry-), sgPTPN1 (BFP+, GFP-, mCherry+), and sgKDM1A/sgPTPN1 (BFP+, GFP+, mCherry+) vs uninfected (BFP+, GFP-, mCherry-).

#### Bulk RNA-seq

K562s expressing dCas9-BFP-KRAB were spinfected in biological duplicate with lentivirus expressing GFP and an sgRNA. Two days after spinfection, the cells were sorted for GFP+ on a BD ARIA II. Sort purity was generally >95%. After the sort, cells were maintained in media supplemented with 4 ug/ml puromycin for four days and then recovered for two days. Cells were counted, collected by centrifugation, and harvested by vigorous vortexing in Qiazol (Qiagen 79306).

Total RNA was extracted with miRNeasy Mini columns (Qiagen 217004) according to the manufacturer’s instructions and sequencing libraries were prepared with TruSeq Stranded Total RNA Library Prep Human/Mouse/Rat kits (Illumina 20020596) according to the manufacturer’s instructions. Libraries were sequenced 2x150 on a NovaSeq (Illumina).

Bulk RNA-seq data were aligned and quantified using STAR (version 2.7.9a) with parameters alignEndsType=Local and outFilter-MultimapNmax=20. For comparison of gene-level expression profiles, gene counts were corrected for sequencing depth (reads per million), and the log_2_ fold-change for each gene was calculated relative to within-replicate non-targeting control expression. The two replicates for each genetic perturbation were averaged in order to produce the final data.

#### Seahorse experiment

K562s expressing dCas9-BFP-KRAB were spinfected with lentivirus expressing GFP and an sgRNA. Two days after spinfection, the cells were sorted for GFP+ on a BD ARIA II. Sort purity was generally >95%. After the sort, cells were maintained in media supplemented with 4 ug/ml puromycin for four days and then recovered for three days. On the 9th day post spinfection, seahorse assay were plates were treated with Cell-Tak (Corning 354240) according to the manufacturer’s instructions. Cells were counted, collected by centrifugation, and resuspended in supplemented Seahorse XF RPMI (Agilent 103576-100). 150,000 cells were added to the Seahorse assay plate and attached via centrifugation at 200xg for 1 minute with no brake. After 30 minutes of recovery at 37°, the cells were subjected to a Mito Stress Test on a Seahorse XFe96 analyzer according to the manufacturer’s instructions.

### QUANTIFICATION AND STATISTICAL ANALYSIS

#### Alignment, cell calling, and guide assignment

Cell Ranger 4.0.0 software (10x Genomics) was used for alignment of scRNA-seq reads to the transcriptome, alignment of sgRNA reads to the library, collapsing reads to UMI counts, and cell calling. The 10x Genomics GRCh38 version 2020-A genome build was used as a reference transcriptome. For specific applications discussed below, STARsolo (STAR version 2.7.9a) was used extract transcript features, including intronic and exonic alignments and alignment of reads to transposable elements.

Reads from the sgRNA libraries were mapped with Cell Ranger. To account for differences in sequencing depths across GEM groups from the same experiment, reads were downsampled to produce a more even distribution of the number of reads per cell across gemgroups, with a threshold of 1000 reads per cell in the K562 day 8 experiment, 800 reads per cell in the K562 day 6 experiment, and 3000 reads per cell in the RPE1 experiment. Guide calling was performed with a Poisson-Gaussian mixture model as previously described. For each guide, the mixture model was fit 100 times, selecting the maximum likelihood model from among the fits. After guide calling, each cell was categorized according to its guide identities as representing a single genetic perturbation or a multiplet (which may arise from lentiviral recombination or multiple cell encapsulation during droplet generation). Only cells bearing two guides targeting the same gene or a single guide were used for downstream analysis.

Downstream analyses were performed in Python, using a combination of numpy, scipy, Pandas, scikit-learn, pomegranate, infercnvpy, pygenometracks, scanpy and seaborn libraries.

#### Filtering and internal normalization of gene expression measurements

Our internal normalization approach is similar to the one described in Adamson et al. (2016). First, we identified ‘‘core’’ control sgRNAs. That is, within each experiment there are tens to hundreds of possible negative control sgRNAs that were synthesized to have similar base compositions to targeting sgRNAs (Horlbeck et al., 2016). Some of these by chance induce detectable phenotypes. We constructed a minimal set of control sgRNAs that are largely indistinguishable from each other using the following procedure: (i) We take all cells bearing all possible non-targeting sgRNAs and represent them by the vector of genes with mean >1 UMI count per cell. (ii) We *z*-normalize the expression of these genes: i.e. we subtract the mean and divide by the standard deviation. (iii) For each gene, we test for equality of distribution using the Anderson-Darling test (scipy.stats.anderson_ksamp) between all possible pairs of non-targeting control sgRNAs. (E.g. In the genome-scale dataset, there are 585 possible control sgRNAs and therefore $\left(\begin{array}{l} 585\\ 2 \end{array}\right)=170280$ pairwise comparisons.) (iv) We adjust the resulting *p*-values for multiple hypothesis testing using the Benjamini-Hochberg procedure. (v) For each potential control sgRNA, we compute the average number of differentially expressed genes relative to all other potential control sgRNAs. (vi) We set a dataset-dependent threshold on the number of differentially expressed genes (8 in the genome-scale dataset and 30 in the ‘‘K562 essentials’’ and ‘‘RPE1 essentials’’ datasets, which were more deeply sequenced and so had more genes passing the expression threshold) and kept all potential control sgRNAs that fell below the threshold. For example, in the genome-scale dataset this resulted in 514 control sgRNAs.

Next, we filtered cells based on quality metrics. We first computed scale factors to adjust for variable sequencing depths across gemgroups: we examined all core control cells (which make up ~4% of all cells), computed factors that equalized the mean UMI counts within these cells across gemgroups, and then applied these factors to all cells in the gemgroup to produce adjusted UMI counts. We then applied two quality filters, ensuring that cells passed a minimum adjusted UMI content filter (genome-scale dataset: 2000 UMIs, K562 essentials/RPE1 essentials: 3000 UMIs) and a maximum mitochondrial RNA filter (genome-scale dataset: <25%, K562 essentials: <20%, RPE1 essentials: <11%). (Mitochondrial RNA content is the fraction of total UMIs derived from mitochondrially-encoded genes.) These filter parameters were chosen by plotting adjusted UMI content vs. mitochondrial RNA content and manually setting thresholds that removed the low-quality cells.

Finally, we computed a normalized gene expression matrix for cells passing the quality filters via two normalization steps: (i) *UMI count normalization*: We scale expression within all cells so that their total UMI counts equal the median UMI count of core control cells within the experiment. (ii) *Relative z-normalization*: Within each gemgroup, for each gene, we compute the mean and standard deviation of expression within control cells and use these to *z*-normalize expression. In other words, if *x* is the expression of a given gene, it is represented by the score $z=\left(x-\mu_{control}\right)/\sigma_{control}$, where the mean and standard deviation are separately computed within each gemgroup.) The resulting scores should therefore be interpreted as ‘‘fraction of transcriptional effort’’ due to the UMI count normalization, with a scale set relative to control cells. Put simply, an expression score of +2 thus represents a gene expressed at a level 2 standard deviations above the mean in control cells.

#### Examining effects of normalization on batch effects

As described in the main text, we observed batch effects in the data (Data S2 [Figure i]). This variation appeared to track mostly with sets of 8 samples that went through the 10x Chromium instrument and library prep together, though the precise origin is unclear. To construct this figure, we normalized the data in two ways. *Raw data normalization*: (i) To adjust for variable sequencing depth, scale cellular UMI counts by factors chosen so that so that core control cells have the same total UMI counts across all gemgroups. (ii) Construct a gemgroup mean expression profile of all genes with mean >2 UMI counts per cell by averaging counts following normalization in previous step across all cells in the gemgroup. (iii) Scale the gemgroup mean expression profiles by dividing by their mean across all gemgroups. An expression value of 1 is then the mean across all cells across all gemgroups. *Internal z-normalization.* Normalize expression as described in previous section.

Data S2 (Figure i) compares the two normalization schemes. In both cases the ranges of the plot are chosen using seaborn’s robust option (which sets the min and max to the 2^nd^ and 98^th^ percentile of the data). Genes are clustered based on the raw data normalization and are in the same order in both panels. The gemgroups are presented in order based on how samples were multiplexed while performing the experiment as indicated by the color groupings at the top.

#### Energy distance test for identifying perturbations that induce altered transcriptional states

To compare distributions of expression states, we used tests derived from energy statistics, which allow for testing of equality of distributions when data are high-dimensional. In short, each cell is represented by a vector composed of its top 20 principal component scores, and we compare whether the distribution of these 20-dimensional vectors is equal or not between unperturbed control cells and cells bearing each perturbation. When these distributions differ, we can infer that the perturbation is causing some change either in the structure or distribution of transcriptional states within the perturbed cells.

To construct the distributions to compare, we first applied a series of filtering steps: (i) we removed cells that did not pass the UMI or mitochondrial RNA filters described in *Internal normalization of gene expression measurements*; (ii) as features, we took the *z*-normalized expression of all genes with mean expression >0.5 UMIs per cell; (iii) to dampen the effects of a handful of strongly induced outlier genes, we clipped any measure with a *z*-score greater than 10 to 10 (this only affects a handful of genes); (iv) finally, we applied principal components analysis (using sklearn’s PCA implementation, which will use randomized algorithms for datasets of this scale) and kept only the top 20 principal components. The test should therefore be interpreted as assessing gross changes in cellular transcriptional state.

To construct a null distribution, we randomly subsampled 5,000 control cells bearing non-targeting sgRNAs. (Subsampling was necessary for performance reasons.) For each perturbation, we then compute an estimator of the energy distance:

$$\varepsilon \left(x,y\right)=\frac{2}{n_{1}n_{2}}\sum_{i=1}^{n_{1}}\sum_{j=1}^{n_{2}}\left\|x_{\text{i}}-y_{\text{i}}\right\|-\frac{1}{n_{1}^{2}}\sum_{i=1}^{n_{1}}\sum_{j=1}^{n_{1}}\left\|x_{\text{i}}-x_{\text{j}}\right\|-\frac{1}{n_{2}^{2}}\sum_{i=1}^{n_{2}}\sum_{j=1}^{n_{2}}\left\|y_{\text{i}}-y_{\text{j}}\right\|$$

where each ***x***_***i***_ is one of the control cells and each ***y***_***j***_ is one of the perturbed cells.

In the limit of infinite data, the energy distance will be 0 between identical distributions and positive between non-identical distributions. We assess statistical significance in practice using a permutation test by permuting the labels of control and perturbed cells 10,000 times and estimating how frequently a larger energy distance would be observed by chance. The specific implementation is based on the python package torch-two-sample, modified to use numba for improved performance.

#### Gene-level differential expression testing using the Anderson-Darling and Mann-Whitney tests

Because of (i) biological differences in expression characteristics across different genes, (ii) the batch effects described above, (iii) incomplete penetrance of some perturbations, and (iv) heterogeneity of some gene expression programs, we opted to use non-parametric statistical tests rather than tests based on specific distributional assumptions about gene expression. Specifically, we *z*-normalize gene expression relative to control cells as described (*Internal normalization of gene expression measurements*) and for each gene test whether the distribution of normalized expression is identical between control cells bearing non-targeting sgRNAs and cells bearing each perturbation. We used two tests implemented in scipy: the Anderson-Darling test (scipy.stats.anderson_ ksamp), which is broadly sensitive to changes in distribution, and the Mann-Whitney U test (scipy.stats.mannwhitneyu), which tests whether one distribution is stochastically greater than another. For the Anderson-Darling test we extended the range of *p* values beyond those available in scipy’s implementation by computing the *p*-value for many values of the test statistic using R’s kSamples package and interpolating any intermediate values using scipy.interpolate.interp1d. For the Mann-Whitney test we used the asymptotic *p* values and excluded any perturbation with fewer than 10 cells. *p*-values in both cases were adjusted for multiple hypothesis testing using the Benjamini-Hochberg procedure to produce the final results.

#### Functional analyses of strong perturbations

We conducted functional analyses to gain insight into the types of gene perturbations that induced strong transcriptional phenotypes. ‘‘Strong’’ perturbations were defined by three criteria: (i) at least 50 differentially expressed genes at a significance of *p* < 0.05 by Anderson-Darling test following Benjamini-Hochberg correction; (ii) at least 25 cells that passed our quality filters; and (3) an on-target knockdown, if measured, of at least 30% (i.e. the target of perturbation was either knocked down by at least 30% or was not detected, a broad attempt to remove non-functional perturbations). ‘‘Weak’’ perturbations met the same criteria but had fewer than 5 differentially expressed genes. Strong and weak perturbations were largely similar in terms of representation and knockdown efficacy, as described in Figure S2. To look for classes of functional behaviors among strong and weak perturbations, we used the gseapy implementation of the Enrichr algorithm to compute gene set enrichment *p*-values within the KEGG2021 pathway gene set (with the set of all targeted genes in the experiment as the background list). To determine whether strong and weak perturbations fell in different subcellular locations, we used location annotations from Itzhak et al. (2016).

#### Global analysis and clustering of strong perturbations

The analysis presented covers 1973 perturbations that met three criteria: (i) at least 50 differentially expressed genes at a significance of *p* < 0.05 by Anderson-Darling test following Benjamini-Hochberg correction; (ii) at least 25 cells that passed our quality filters; and (3) an on-target knockdown, if measured, of at least 30% (i.e. the target of perturbation was either knocked down by at least 30% or was not detected, a broad attempt to remove non-functional perturbations). As features, we used a union of two sets of genes: (i) the top 10 differentially expressed genes for all perturbations (ordered by the value of the Anderson-Darling test statistic) and (ii) all genes of mean >0.25 UMIs per cell with variance in the top 30% of the dataset. We represented perturbations by their mean normalized expression profile across these 2319 highly variable genes. To prevent the direct targets of knockdown influencing results, the target gene value was replaced by 0 for the corresponding perturbation. For example, RPS5 gene normalized expression was set to 0 in the expression profile of the RPS5 perturbation, which is equal to the mean in control cells by construction.

Because clearly related perturbations sometimes showed variable absolute phenotypic strengths, we used correlation as a metric to compare profiles, since it is scale-invariant. We conducted two global assessments of the ability of these expression profile correlations to recall known biological relationships. First, curated complexes were obtained from the 03.09.2018 CORUM3.0 database (Giurgiu et al., 2019). We identified all complexes that had at least 66% of genes represented within the 1973 perturbations (based on matching gene symbols between the datasets), leading to 327 complexes. Each represented complex was then split into a series of links (e.g. if a complex contained genes A, B, and C, then it would be split into links A-B, B-C, and A-C). The figure plots the distribution of expression profile correlations of these annotated links versus the distribution of all possible links among the 1973 targeted genes. A similar analysis was then conducted using predicted protein links from the v11.5 of STRING (Szklarczyk et al., 2019) (9606.protein.links.v11.5.txt.gz) after mapping STRING protein IDs to gene names (using the ‘‘preferred_name’’ field in 9606.protein.info.v11.5.txt.gz). Among the 1973 genes in the figure there are 1,945,378 possible pairwise links between genes, 243,558 of which have scores within STRING. We binned these represented links into 6 equally spaced bins based on observed expression profile correlation. The figure shows kernel density estimates of the STRING score distribution within each bin made using seaborn’s violinplot (with cut set to 0 so that density estimates do not extend past observed data).

To identify clusters of related perturbations, we manually computed correlation distances between all pairs of expression profiles, and used HDBSCAN (metric=’precomputed’, min_cluster_size=4, min_samples=1, cluster_selection_method=’eom’) to identify 63 clusters. This procedure is intrinsically conservative due to the choice of metric and clustering algorithm, so many perturbations are not assigned to any cluster—our emphasis was on identifying the strongest signals rather than the most comprehensive. We then annotated the possible function of these clusters using a combination of manual lookup of related genes and automated annotation using CORUM complexes and STRING clusters. (STRING clusters are derived from 9606.clusters.info.v11.5.txt.gz and are labeled according to the ‘‘best_described_by’’ field.) We only assigned automated annotations when a cluster contained 75% or more of the members of a CORUM complex or STRING cluster. Aggregated information about clusters is provided in Table S3 which has the following fields:

**Table:** 

members	The genes assigned to the cluster by HDBSCAN
**nearby_genes**	Perturbations that are close to the cluster center in the high-dimensional embedding (see below). These are candidate members of the cluster that may be too weak to be called by HDBSCAN, which is quite conservative.
**emb_variable_x**	Location of cluster center in Figure 2D
**emb_variable_y**	Location of cluster center in Figure 2D
**manual_annotation**	Manual annotation of cluster function.
**contained_string_cluster_ids**	IDs of any STRING clusters contained within this cluster (at least 75% of members must be represented).
**contained_string_clusters**	Descriptions of any STRING clusters contained within this cluster (at least 75% of members must be represented).
**contained_corum_complexes**	Names of any CORUM complexes contained within this cluster (at least 75% of members must be represented).
**nearby_corum_complexes**	Names of any CORUM complexes that are close to this cluster in the high-dimensional embedding (at least 75% of members must be represented).
**nearby_string_clusters**	Names of any STRING clusters that are close to this cluster in the high-dimensional embedding (at least 75% of members must be represented).
**strong_positive_gene_expr_clusters**	Gene expression programs (see Figure 4) for which average expression in this cluster is at least 2 standard deviations above normal expression level.
**strong_negative_gene_expr_clusters**	Gene expression programs (see Figure 4) for which average expression in this cluster is at least 2 standard deviations below normal expression level.
**best_description**	Either the manual annotation or identity as called by CORUM/STRING. Most of the labels in Figure 2D come from this field.
**example_genes**	Manually curated genes indicative of cluster function.
**Notes**	Notes, including any known off-targets.

#### Minimum distortion embedding of strong perturbations

The visualization in Figure 2D is a minimum distortion embedding (MDE) of the 1973 strong perturbations created using pymde v0.1.13. pyMDE solves MDE problems based on minimizing Euclidean distances. To adapt it to correlation distances, we first *z*-normalized each expression profile. (I.e. If a perturbation is represented by the vector ***x*** of 2319 highly variable genes, we computed $\hat{x}=\left(x-x\right)/\sigma_{x}$.) Because of the polarization identity, minimizing the Euclidean distance between these normalized profiles is equivalent to minimizing the (square root of) the correlation distance between the unnormalized profiles:

$${\left\|\hat{x}-\hat{y}\right\|}^{2}={\left\|\hat{x}\right\|}^{2}+{\left\|\hat{y}\right\|}^{2}-2\left(\hat{x}\cdot \hat{y}\right)=2\left(1-corr\left(x,y\right)\right)$$

where the second equality follows from the *z*-normalization and the scale-invariance of correlation.

We created two embeddings. First, we used pymde to embed the dataset into 20 dimensions. This ‘‘high-dimensional embedding’’ serves as an imputation step, as it distorts the geometry of the dataset so that clusters of related genes that may be driven by weaker overall correlations are allowed to form. Proximity within this embedding was used to identify genes, CORUM complexes, and STRING clusters that were near to the HDBSCAN clusters called on the raw data (which were well-preserved in the embedding) as described in Table S3. To construct the embedding, we initialized pymde using the spectral embedding of the dataset (using sklearn’s SpectralEmbedding with n_components=20, affinity=’nearest_neighbors’, n_neighbors=7, eigen_solver=’arpack’)), and then ran pymde’s ‘‘preserve_neighbors’’ function with embedding_dim=20, n_neighbors=7, and repulsive_fraction=5. pymde was run until convergence with a final average distortion of 0.0979 and final residual norm of 9.4e-06.

To produce the embedding in Figure 2D, we ran pymde with the same parameters but with the embedding dimension set to 2 (final average distortion 0.105, final residual norm 3.2e-06). The bold cluster labels in the figure correspond to the manual annotations mentioned in the previous section. A handful of changes were manually incorporated: (1) the cytochrome c-ubiquinol cluster was not detected by HDBSCAN, and was manually annotated (2) 4 clusters involving protein post-translational modifications (ubiquitination, sumoylation, acetylation, neddylation) were annotated with a single label of ‘‘post-translational modifications’’ (3) components of eIF3 split across two clusters that are next to each other in the embedding and are labeled as a single cluster (4) all clusters of unknown function were not labeled but are included in Table S3. The complex labels come from CORUM or STRING. A complex/cluster label was placed if and only if 75% of the members of a complex or cluster were close to each other in the 20-dimensional pyMDE embedding (‘‘close’’ meaning at or below the 5^th^ percentile of all pairwise distances). Redundant/duplicated clusters were manually deduplicated. The locations of the labels on the figure were then adjusted for readability. Label location is a decent proxy for, but not an entirely accurate representation of, cluster and complex locations.

#### Clustering of gene expression programs

We next turned to identifying conserved gene expression programs using a similar pipeline applied to the transpose of the expression matrix from the previous sections. Initial HDBSCAN clustering on the raw data did not yield very many clusters, which we attributed to the broad range in gene expression program size and dynamic range. To attempt to equalize for these factors, we performed the clustering on a high-dimensional embedding of the data. Each gene was represented by its expression across the 1973 perturbations in Figure 2 and we masked the targets of knockdown as described there to avoid target gene knockdown influencing clustering. We used pyMDE with the same normalization as above to encourage genes with correlated expression to be placed nearby to each other (20 dimensions, n_neighbors=7 and repulsive_fraction=5, final average distortion 0.145, final residual norm 5.1e-06). We then identified clusters using HDBSCAN applied to the embedding (metric=’euclidean’, min_cluster_size=10, min_samples=10, cluster_selection_method=’leaf’), producing 38 clusters. We performed similar analyses as in Figure 2 to annotate known CORUM complexes and STRING clusters. Cluster identities were then manually annotated using a combination of these automated annotations, manual gene searches, and gene set enrichment analyses conducted using Enrichr (Xie et al., 2021). These clusters are summarized in Table S3, which includes the fields:

**Table:** 

members	The genes assigned to the cluster by HDBSCAN.
**string_cluster_ids**	IDs of any STRING clusters contained within this cluster (at least 75% of members must be represented).
**string_clusters**	Descriptions of any STRING clusters contained within this cluster (at least 75% of members must be represented).
**corum_complexes**	Names of any CORUM complexes contained within this cluster (at least 75% of members must be represented).
**nearby_corum_complexes**	Names of any CORUM complexes that are close to this cluster in the high-dimensional embedding.
**nearby_string_clusters**	Names of any STRING clusters that are close to this cluster in the high-dimensional embedding.
**strong_positive_clusters**	Perturbation clusters (see Figure 2) for which average expression of this expression program is at least 2 standard deviations above normal expression level.
**strong_negative_clusters**	Perturbation clusters (see Figure 2) for which average expression of this expression program is at least 2 standard deviations below normal expression level.
**manual_annotation**	Manual annotation of cluster function.
**example_genes**	Manually curated genes indicative of cluster function.

To produce Figure 3B, we averaged expression within the 64 perturbation clusters from Figure 2 across the genes within the 38 gene expression clusters. Each element in the heat map therefore represents an average over both multiple related perturbations and multiple related genes. The labels were manually selected to highlight interesting features.

#### Screens of gene expression programs

To demonstrate the ability of Perturb-seq to conduct screens on aggregate phenotypes, we conducted two analyses to identify perturbations that strongly induced interesting expression programs. In the first comparison, we compared expression of genes associated with erythroid differentiation (gene expression cluster 15) to those associated with myeloid differentiation (gene expression cluster 21) in Figure 4D. We scored expression programs by taking the mean normalized gene expression of all genes in the associated clusters. We computed scores for all perturbations in the genome-scale dataset that were detected in at least 25 cells, and then *z*-normalized these scores to make scales comparable. The figure has labels on the 15 most outlying genes across the two programs. We then conducted an identical analysis comparing expression of an unfolded protein response cluster (cluster 2) to an integrated stress response cluster (cluster 12) in Figure 4C.

#### Analysis of composite phenotypes: total RNA, fraction mtRNA, fraction TE, RNA splicing

Composite phenotypes integrate data from across the transcriptome to describe global cellular features. While some derive from simple metrics, others rely on extracting information from the transcriptome beyond gene expression levels. As an example of a simple metric, the number of UMIs aligned to the transcriptome (GRCh38 version 2020-A) of single-cells was used to represent the total cellular RNA content. To produce Figure 4H, we averaged the total RNA content of cells for each perturbation, and z-scored the RNA content with respect to non-targeting controls. Similarly, to calculate the fraction of mitochondrial RNA per cell (fraction mtRNA), the sum of the expression levels of the 13 mitochondrial genome protein-coding genes (MT-ND6, MT-ND1, MT-ND2, MT-ATP8, MT-ND4L, MT-ND5, MT-ND3, MT-CO1, MT-CO2, MT-ND4, MT-ATP6, MT-CO3, MT-CYB) was divided by the total cellular RNA content for each individual cell.

The scTE (He et al., 2021) processing pipeline was used to quantify the expression of transposable elements in single cells. As transposable elements tend to be present in many degenerate copies throughout the genome, scTE allocates TE reads to TE metagenes rather than specific genomic positions. Reads were aligned to the genome using STARsolo (STAR version 2.7.9a) with the flags ‘ –outSAMattributes NH HI AS nM CR CY UR UY –soloFeatures Gene GeneFull SJ Velocyto –readFiles Command zcat –outFilterMultimapNmax 100 –winAnchorMultimapNmax 100 –outMultimapperOrder Random –runRNGseed 777 –outSAMmultNmax 1’ to allow multimapping. To avoid incompatibilities in cell calling between STARsolo and Cell Ranger, the output of Cell Ranger cell calling was used to define the STARsolo cell barcode whitelist using ‘–soloCBwhitelist’. Next, aligned reads were allocated to genes and TEs using scTE. The flag ‘-o nointron’ was used to prevent the quantification of TEs in gene introns. From single-cell transcriptomes, quantification of all TEs in the classes LINE, SINE, LTR, DNA, and Retroposon based on RepeatMasker were extracted. To calculate the fraction of repetitive and transposable element RNA per cell (fraction TE), the sum of the expression level of these TEs was divided by the total cellular RNA content for each individual cell. To produce Figure 4G, we averaged the fraction TE of cells for each perturbation.

The alignments from STARsolo described above were also be used to quantify RNA splicing. Due to (i) the sparsity of single-cell data (ii) the relationship between the fraction of spliced reads and gene expression levels, gene-wise RNA splicing was quantified at the pseudobulk level. From the STARsolo Velocyto output, the levels of spliced and unspliced reads for each gene were averaged across all cells bearing each perturbation, ignoring ambiguous reads. Then, for each gene, the fraction of unspliced reads was divided by the mean fraction of unspliced reads for that gene across all non-targeting control perturbations. The results in Figure 3F display a common set of genes across all perturbations.

#### Leverage scores for quantifying perturbation penetrance and variability

Our use of non-parametric tests in differential expression testing is in part to accommodate perturbations that may be incompletely penetrant or heterogeneous in effect. To attempt to quantify these features, we developed a scalar single-cell score to summarize how outlying each cell’s transcriptional state was relative to control cells. We used an approach based on leverage scores, which measure how outlying the rows or columns of a matrix are and which form the basis for many randomized algorithms (Ma et al., 2013). Specifically, we: (i) Construct an expression matrix consisting of all cells that pass the quality filters described in *Internal normalization of gene expression measurements*, and all genes with mean expression >0.25 UMI counts per cell. (ii) To dampen the effects of a handful of strongly induced outlier genes, we clipped any measure with a *z*-score greater than 10 to 10 (this only affects a handful of genes) (iii) To avoid the influence of gemgroup-level batch effects and variable sequencing depth, we then compute leverage scores separately within each gemgroup. Row leverage scores, corresponding to the cell axis of the expression matrix, are calculated as the squared norm of the top 20 left singular vectors within each gemgroup (computed via the truncated SVD routine scipy.sparse.svds with the arpack solver with k=20). We then normalize these scores so that the sum over all cells in the gemgroup is 1 (i.e. compute the leverage sampling probability distribution). (iv) Finally, to integrate leverage scores across gemgroups, we then take logs, and rescale by *z*-normalizing relative to the scores of control cells (subtracting their mean and dividing by their standard deviation). All the leverage scores presented in the figures are the leverage scores after this normalization procedure.

In Figure S6 we conducted various analyses to validate leverage scores as measures of phenotype and to use them to study penetrance of perturbations. We considered all perturbations that passed the following criteria: (i) >5 differentially expressed genes by Anderson-Darling test; (ii) detected in at least 25 cells that passed our quality filters; and (iii) the gene targeted by the perturbation was either undetectable in the expression data or knocked down by at least 30% if detected (i.e., we removed perturbations that appear non-functional). For these perturbations we compared the mean leverage scores to the number of differentially expressed genes found using the Anderson-Darling test. To assess reproducibility we then subset to perturbations that were present in both the genome-scale dataset and the K562 essentials dataset, including non-targeting controls.

In the analyses of perturbations targeting Mediator and the small subunit of the ribosome we only included perturbations that were (i) present in both the genome-scale dataset and the ‘‘K562 essentials’’ dataset and (ii) targeted the principal ‘‘P1’’ transcript identified by the FANTOM consortium. (A handful of genes also had perturbations targeting the P2 transcript that did not generally have effects.) Knockdown was computed as the ratio of mean (unnormalized) expression of the target gene within perturbed cells vs. that in cells with non-targeting sgRNAs. The plots show kernel density estimates of the distributions of the leverage scores of all cells with these perturbations constructed using seaborn’s violinplot (with cut set to 0 so that estimated distributions do not extend beyond the range of the data). The gray bars represent the 10%-90% quantiles of cells with non-targeting control sgRNAs for comparison.

Finally, in Figure 5B we used leverage scores to search for perturbations that had highly variable phenotypes. We considered all perturbations using the same criteria as in fig. S6. We used the standard deviation of the leverage scores as a metric for variability, as diagrammed in Figure 5A. The two examples in this figure are derived from actual data. The 20 labeled genes are the most outlying from the lowess local regression between the standard deviation of leverage scores and the log of the number of differentially expressed genes detected by the Anderson-Darling test (computed using statsmodels.nonparametric.smoothers_lowess.lowess).

#### Analysis of chromosomal instability and cell cycle

The framework described in inferCNV (Patel et al., 2014) and implemented as infercnvpy (https://github.com/icbi-lab/infercnvpy) was used to detect evidence of chromosomal copy number changes. The raw single-cell gene expression matrix was first filtered to remove lowly expressed genes (<0.05 UMIs per cell across the population), normalized for total UMI content (using scanpy.pp.normalize_total with target_sum=1e6, exclude_highly_expressed=False, and max_fraction=0.05), and log-scaled (using scanpy.pp.log1p). Then we used infercnvpy to compute rolling average gene expression changes for windows of 100 genes with a dynamic threshold of 1.5 standard deviations for noise filtering (using infercnvpy.tl.infercnv). For each single-cell, this generated a vector of CIN values across the genome. To label cells with likely karyotypic abnormalities, unstable karyotypic cells were heuristically defined as having ≥1 chromosome with evidence of changes in chromosomal copy number (nonzero CIN values) for >80% of the chromosomal length. For Figure 5G, the CIN score of genetic perturbations was calculated as the mean single-cell sum of squared CIN values, *z*-normalized relative to non-targeting control perturbations.

To show cell cycle effects in Figures 5D and 5E, we chose to use a dimension reduction approach. Previous approaches to cell cycle analyses in Perturb-seq have largely focused on supervised classification of cells into canonical cell cycle states. However, we found that these approaches did not allow for aberrant cell cycle states sometimes generated by genetic perturbations. As a summary of single-cell cell cycle states, we performed a Uniform Manifold Approximation and Projection (UMAP) dimension reduction based on the expression n=199 known cell cycle genes [obtained from Seurat (Satija et al., 2015) and (Adamson et al., 2016)]. From total UMI content normalized, log-scaled expression data, a neighborhood graph was computed (using scanpy.pp.neighbors with n_neighbors=30, method=’umap’, metric=’correlation’, and n_pcs=20) followed by UMAP embedding (using scanpy.tl.umap with default parameters). This UMAP revealed cells in canonical cell cycle stages when compared with other methods, but also naturally separated dying cells and putatively quiescent cells. Gates were drawn by manual inspection to approximately separate cells likely to be in S, G2/M, and G1/G0 cell cycle phases.

#### Analysis of transcriptional responses to mitochondrial stress

To compare the functional specificity of nuclear and mitochondrial genome responses to mitochondrial stress, we clustered mitochondrial perturbations based on their transcriptional phenotypes. The analysis presented in Figures 6A and 6C (K562 day 8 data) covers 268 mitochondrial perturbations that met three criteria: (i) at least 50 differentially expressed genes at a significance of *p* < 0.05 by Anderson-Darling test following Benjamini-Hochberg correction; (ii) at least 30 cells; and (iii) an on-target knockdown of at least 60%. As features, we used 1715 genes encoded in the nuclear genome expressed at >1 UMI per cell or the 13 protein-coding mitochondrial-encoded genes. Analogously, the analysis presented in Figures S12A and S12E (RPE1 day 7 data) covers 140 mitochondrial perturbations that met three criteria: (i) at least 20 differentially expressed genes at a significance of *p* < 0.05 by Anderson-Darling test following Benjamini-Hochberg correction; (ii) at least 30; and (iii) an on-target knockdown of at least 60%. As features, we used 2017 genes encoded in the nuclear genome expressed at >1 UMI per cell or the 13 protein-coding mitochondrial-encoded genes.

To cluster these data, we used correlation as a metric to compare z-normalized expression profiles, since it is scale-invariant. We clustered using HDBSCAN (metric=’correlation’, min_cluster_size=3, min_samples=1, cluster_selection_method=’eom’, alpha=1.0). As discussed above, this procedure is intrinsically conservative due to the choice of metric and clustering algorithm, so many perturbations are not assigned to any cluster. We used the hierarchical clustering output of HDBSCAN to manually identify and label groups of perturbations in the figures.

To compare the heterogeneity of mitochondrial genome expression, we used two approaches. First, we used a data-driven approach comparing the 38 gene expression programs described in Figure 4B across perturbations. For each program, we scored its expression within each genetic perturbation, and calculated a standard deviation of these scores across the different perturbations in the K562 day 8 experiment. The mitochondrial genome program discovered in this way consists of the 13 protein-coding mitochondrial genes, plus two MT-RNR2-like pseudogenes encoded in the nuclear genome which are likely to multimap with mitochondrial-encoded transcripts. In Figure S12C, we show a histogram of the variability of expression programs across perturbations. Second, we used a hypothesis-driven approach that compared the variation of mitochondrial genome responses by the protein localization of perturbations. We used data from the Human Protein Atlas to assign locations to different perturbations (https://www.proteinatlas.org/about/download table subcellular_location.tsv.zip; some locations were collapsed into supersets), excluding any dual-localized proteins. For all perturbations with the same localization and at least 50 differentially expressed genes at a significance of *p* < 0.05 by Anderson-Darling test following Benjamini-Hochberg correction, we calculated the variance of z-normalized gene expression profiles for each mitochondrially encoded gene. The data in Figure 7B (K562 day 8) and S12B (RPE1) represents the average variance across mitochondrially-encoded genes for each localization.

To quantitatively compare the specificity of the mitochondrial and nuclear transcriptional responses, we employed random forest classifiers. The analysis presented in Figure S12F (K562 day 8 data) covers perturbations that met three criteria: (i) cause at least 50 differentially expressed genes at a significance of *p* < 0.05 by Anderson-Darling test following Benjamini-Hochberg correction; (ii) at least 30 cells; and (iii) belong to either the small or large mitochondrial ribosomal subunit (identified by gene names beginning with MRP), ATP synthase (identified by gene names beginning with ATP5), proteostatic factors (including PAM16, DNAJC19, TFAM, HSPA9, TOMM40, TOMM20, PMPCB, LONP1, and HSPE1). As features, we used 2039 genes encoded in the nuclear genome expressed at >0.5 UMI per cell with variance in the upper 25% of the dataset, or the 13 protein-coding mitochondrial encoded genes. To train the random forest classifier to separate cells by perturbed complex (as described above), we used the scikit-learn implementation with extremely randomized trees with 1,000 trees in the forest and 100 features. The reported accuracy is the balanced accuracy defined as the defined as the average of recall obtained on each class. To visualize the data, we used a UMAP (metric=’correlation’, n_neighbors=10) generated on the nuclear or mitochondrial genome.

## Supplementary Material

1

2

3 Figure S1. Growth screens, filtering, and coverage, related to Figure 1
(A) Comparing the growth phenotypes of dual-sgRNA constructs between growth screen replicates in K562 cells. Growth phenotypes are reported as the log_2_ guide enrichment per cell doubling (gamma) between day 6 and day 16 post library transduction. Replicates are strongly correlated (n = 11,056 dual-sgRNA constructs; r = 0.94). For 50 outlier genes (where the residual from a regression comparing replicates was >0.2), the growth phenotype was set to missing.
(B) Benchmarking the growth phenotypes of dual-sgRNA constructs to single-sgRNA screens. Growth phenotypes (gammas) are compared between the dual-sgRNA library compared with the mean of the best three sgRNAs from Horlbeck et al. The screens are strongly correlated (n = 9,386 genes after excluding constructs mapping to secondary transcription start sites [TSSs]; r = 0.85) but with stronger growth phenotypes observed for the dual-sgRNA library.
(C) Comparing the growth phenotypes of dual-sgRNA constructs between growth screen replicates in RPE1 cells. Growth phenotypes are reported as the log_2_ guide enrichment between the plasmid library and day 7 post library transduction. Replicates are strongly correlated (n = 2,203 constructs targeting common essential genes; r = 0.87). For 19 outlier genes (where the residual from a regression comparing replicates was >1), the growth phenotype was set to missing.
(D) Comparing growth phenotypes between K562 and RPE1 cells. Growth phenotypes are correlated (n = 1,951 constructs; r = 0.53) despite substantial differences in screen time point (day 6 to day 16 for K562 cells versus day 0 to day 7 in RPE1 cells).
(E) Schematic overview of data alignment, cell calling, sgRNA assignment, and filtering.
(F) Histogram of the number of cells per genetic perturbation in the K562 day 8 genome-wide Perturb-seq experiment. The number of detected genetic perturbations (expected sgRNA pairs) was n = 11,258, with a mean coverage 183 cells per perturbation and a median coverage of 171 cells per perturbation after filtering.
(G) Histogram of the number of cells per genetic perturbation in the K562 day 6 essential-wide Perturb-seq experiment. The number of detected genetic perturbations (expected sgRNA pairs) was n = 2,285, with a mean coverage 148 cells per perturbation and a median coverage of 124 cells per perturbation after filtering.
(H) Histogram of the number of cells per genetic perturbation in the RPE1 cell day 7 essential-wide Perturb-seq experiment. The number of detected genetic perturbations (expected sgRNA pairs) was n = 2,679, with a mean coverage 101 cells per perturbation and a median coverage of 79 cells per perturbation after filtering.

4 Figure S2. Differential expression and enrichment analyses, related to Figure 1
(A) Relationship between the number of differentially expressed genes (DEGs) for a genetic perturbation in K562 cells at day 8 versus day 6 post-transduction. DEGs were determined using a two-sample Anderson-Darling test comparing against non-targeting guides (n = 2,276 common genetic perturbations, Spearman’s rho = 0.78).
(B) Relationship between the number of DEGs for a genetic perturbation in K562 cells (day 8 genome-wide dataset) versus RPE1 cells. DEGs were determined using a two-sample Anderson-Darling test comparing against non-targeting guides (n = 2,636 common genetic perturbations, Spearman’s rho = 0.53).
(C) Comparing the growth phenotype versus the number of DEGs for each multiplexed guide pairs in RPE1 cells. Growth phenotypes are reported as the log_2_ guide enrichment between day 0 and day 7 post-lentiviral transduction. DEGs were determined using a two-sample Anderson-Darling test comparing against non-targeting guides.
(D) Relationship between features of genetic perturbations in K562 cells genome-wide day 8 Perturb-seq. The features were calculated as detailed in STAR Methods. The heatmap displays Spearman correlations between features.
(E) The distribution of growth phenotypes in genetic perturbations with a transcriptional phenotypes in K562 cells genome-wide day 8 Perturb-seq. Histogram (kernel density estimate) comparing the growth phenotype in K562 cells (gamma) of genetic perturbations to the permuted energy distance test. 771 genetic perturbations had a gamma >—0.1 (considered a negligible effect on cellular growth) but a significant transcriptional phenotype.
(F) KEGG pathway enrichment for genetic perturbations causing strong and weak transcriptional phenotypes in the K562 day 8 dataset. Strong perturbations were defined as having (i) >50 differentially expressed genes at a significance of p < 0.05 by Anderson-Darling test following Benjamini-Hochberg correction (ii) >70% on-target knockdown when the target gene was detected. Weak perturbations were defined as having (i) <5 differentially expressed genes at a significance of p < 0.05 by Anderson-Darling test following Benjamini-Hochberg correction (ii) >70% on-target knockdown when the target gene was detected. Enrichr was used to perform enrichment in the KEGG 2021 pathways with the top 20 most significant pathways shown.
(G) Barplot of the number of essential versus nonessential genes for strong and weak perturbations in the K562 day 8 dataset.
(H) Histogram of the target gene expression level in K562 cells for strong and weak genetic perturbations.
(I) Histogram of the number of cells detected for strong and weak genetic perturbations.
(J) Histogram of the percent knockdown of the target gene for strong and weak genetic perturbations.
(K) Barplot of the number of genes with known protein localization for strong and weak perturbations.
(L) Barplot of the protein localization for strong and weak perturbations.

5 Figure S3. Assessing neighbor gene off-target knockdown in Perturb-seq data, related to Figure 1
(A and B) Relationship between neighbor gene off-target knockdown and position relative to the target gene in K562 cells (day 8) (A) and RPE1 cells (B). For each target genes, the two neighbor genes are defined as the gene immediately upstream and downstream (at an expression >0.1 UMI per cell). The position relative to the target is the distance of either the start or end of the neighbor gene (whichever is closer) to the start of the target gene. The fractional change in expression is defined as the expression in the targeted cells minus the expression in non-targeting cells, relative to the expression in the non-targeting cell population (—1 implies 100% knockdown).
(C and D) Comparison between target gene and neighbor gene knockdown in K562 cells (day 8) (C) and RPE1 cells (D). p values are assigned by comparing a bootstrap test.
(E) Comparison of neighbor gene knockdown in K562 cells (day 8) versus RPE1 cells.
(F) Comparison of neighbor gene knockdown based on transcriptional phenotype in K562 cells (day 8). Perturbations with ‘‘transcriptional phenotype with negligible growth phenotype’’ are those perturbations where gamma >—0.1 that had a significant transcriptional phenotype by the permuted energy distance test.

6 Figure S4. Defining gene function with Perturb-seq, related to Figure 2
(A) Comparison of perturbation relationships as derived from independent K562 day 8 and K562 day 6 datasets. We analyzed the union of 1,206 genetic perturbations that elicited transcriptional phenotypes (>10 differentially expressed genes) in all three datasets. Pearson correlations were used to summarize perturbation-perturbation relationships calculated on *z*-normalized gene expression profiles across well-expressed (>0.25 UMIs per cell) and highly variable genes. The cophenetic correlation between datasets is r = 0.82.
(B) Correlation of transcriptional phenotypes between datasets. We analyzed the intersection of genetic perturbations that elicited transcriptional phenotypes (>10 differentially genes) between each pair of datasets. For each genetic perturbation, we calculated the Pearson correlation of *z*-normalized gene expression profiles across well-expressed (>0.5 UMIs per cell) genes. The comparison of K562 day 8 versus K562 day 6 (red) and K562 day 8 versus RPE1 day 7 (blue) are shown.
(C) Comparison of perturbation relationships as derived from independent K562 day 8 and RPE1 datasets. We analyzed the union of 1,206 genetic perturbations that elicited transcriptional phenotypes (>10 differentially expressed genes) in all three datasets. Pearson correlations were used to summarize perturbation-perturbation relationships calculated on *z*-normalized gene expression profiles across well-expressed (>0.25 UMIs per cell) and highly variable genes. The cophenetic correlation between datasets is r = 0.37.
(D) KEGG pathway enrichment for genetic perturbations with well-correlated (r > 0.5) transcriptional phenotypes between the K562 day 8 and RPE1 day 7 datasets. Enrichr was used to perform enrichment in the KEGG 2021 pathways with the significant (adjusted p < 0.05) pathways shown.
(E) KEGG pathway enrichment for genetic perturbations with uncorrelated (*r* > 0) transcriptional phenotypes between the K562 day 8 and RPE1 day 7 datasets. Enrichr was used to perform enrichment in the KEGG 2021 pathways with the significant (adjusted p < 0.05) pathways shown.
(F) Relationship between members of ribosomal subunits, biogenesis factors, and poorly characterized genes. The heatmap displays the Pearson correlation between pseudobulk *z*-normalized gene expression profiles of select genes. Genetic perturbations are ordered by average linkage hierarchical clustering based on Euclidean distance in K562 cells (day 8). Poorly characterized genes are shown in bold.
(G) Investigation of *ATF5* phenotype in K562 cells (day 8). The upper heatmap shows the correlation of the expression profile of *ATF5* knockdown with similar genetic perturbations. The sgRNA pair targeting *ATF5* leads to a phenotype strongly correlated with knockdown of subunits of the nuclear pore complex (*NUP54*, *NUP62*) and nuclear export proteins (*XPO1*). The sgRNA pair targeting *ATF5* leads to strong downregulation of NUP62 (bottom heatmap, shown as the log_2_-fold change compared with control cells). *ATF5* is bidirectionally expressed with *NUP62* on chromosome 19, explaining this neighbor gene off-target knockdown and similar phenotypes.
(H) Investigation of other surprising phenotypes in K562 cells (day 8). We investigated three different surprising relationships between genetic perturbations: clustering of *TMEM215* with LAMTOR subunits, clustering of *CLOCK* with NMD machinery, and clustering of *PGAM5* and *RMDN3* with the mitochondrial large ribosomal subunit (upper heatmaps). In all three cases, the relationship could be explained by suspected off-target knockdown of a component of the complex, directly detected in Perturb-seq (bottom heatmaps).

7 Figure S5. Genotype-phenotype relationships, related to Figure 4
(A) Heatmap of the high-level genotype-phenotype map (identical to Figure 4B with full labels). The heatmap represents the mean *Z* scored expression for gene expression and perturbation clusters. For a subset of clusters, clustered are labeled with manual annotations (black labels) of cluster function.
(B) Growth effect of *PTPN1* or *KDM1A* knockdown in K562 cells. Cells were co-transduced with fluorescently labeled sgKDM1A, sgPTPN1, or a non-targeting control guide. Enrichment was determined by flow cytometry relative to uninfected cells in biological triplicate.
(C) Comparison of transposable element expression profiles between top regulators. Heatmap displays log_2_-fold changes in expression of highly expressed transposable element metagenes (columns) for genetic perturbations (rows) in K562 cells (day 8) Perturb-seq. Genetic perturbations and genes are ordered by average linkage hierarchical clustering with a Euclidean distance metric.
(D) Comparison of total RNA content with cell-cycle state. For single cells, cell-cycle positioning was inferred by UMAP dimension reduction on differential expression profiles of 199 selected cell-cycle regulated genes. The dimension reduction was performed independently for RPE1 cells (left) and K562 cells (right). Cell-cycle occupancy is shown as a scatterplot of UMAP positions of a random subset of 10,000 cells per cell type. Approximate gates between cell-cycle phases (G1 or G0; S; G2 or M) are shown as dotted lines. The total RNA content per cell was calculated from the total number of UMIs detected per cell which were *Z* scored with respect to gemgroup/lane control cells.

8 Figure S6. Assessing the penetrance and heterogeneity of response to genetic perturbations, related to Figure 5
(A) We scored how outlying each perturbed cell was relative to non-targeting control cells using leverage scores. The plot compares the mean leverage score for each genetic perturbation to the number of differentially expressed genes detected by the Anderson-Darling test (Spearman’s rho = 0.71).
(B) To assess reproducibility of leverage scores, plot compares mean leverage scores of perturbations (black dots) in K562 cells between the essentials dataset (taken at day 6 post-infection) and the dataset targeting all expressed genes (taken at day 8 post-infection). Non-targeting control sgRNAs are in orange (Spearman’s rho = 0.79).
(C) Relationship between knockdown of target gene (relative to expression in control cells bearing non-targeting sgRNAs) and number of differentially expressed genes detected by Anderson-Darling test for perturbations targeting subunits of the Mediator complex.
(D) Leverage scores distributions of perturbations targeting subunits of the Mediator complex. Plot shows kernel density estimates for each perturbation ordered from least knocked down (left) to most (right). Top panel is within essentials dataset and bottom panel is within the all expressed genes dataset. The gray bar shows the 10%–90% range of leverage scores within control cells bearing non-targeting sgRNAs.
(E and F) As in (C) and (D) but for perturbations targeting the small subunit of the ribosome.

9 Figure S7. Chromosomal instability, related to Figure 5
(A) Heatmap of chromosomal copy-number inference from Perturb-seq data. For all genes (expressed >0.05 UMI per cell), the log-fold change in expression is calculated with respect to the average of non-targeting control cells, and genes are ordered along the genome. A weighted moving average of 100 genes is used infer copy-number changes (columns) in single cells (rows) with noise and median filtering. 199 *TTK* knockdown K562 cells and 199 randomly sampled non-targeting control K562 cells are shown (data from K562 essential-wide day 6 dataset). Cells are ordered by average linkage hierarchical clustering based on correlation of chromosomal copy-number profiles.
(B) Comparison of cell-cycle effects by magnitude of karyotypic abnormality in RPE1 cells. RPE1 cells with at least one chromosomal loss (defined as evidence of chromosomal loss for >80% of the chromosomal length) were stratified into high, medium, and low degree of karyotypic abnormality based on their CNV score. 500 randomly sampled high and low CNV score cells were visualized in a heatmap of chromosomal copy-number inference. Below, the cell-cycle occupancy of high and low CNV cells is shown for 1,000 randomly sampled cells. For single cells, cell-cycle positioning was inferred by UMAP dimension reduction on differential expression profiles of 199 selected cell-cycle regulated genes. Cell-cycle occupancy is shown as a 2D kernel density estimate of a random subset of 1,000 cells per karyotypic status. Approximate gates between cell-cycle phases (G1 or G0; S; G2 or M) are shown as dotted lines, and the fraction of cells in each cell-cycle phase are indicated.
(C) Effect of chromosomal instability (CIN) on activation of the integrated stress response (ISR). Histogram (kernel density estimate) compares the ISR score versus CIN status in K562 cells (day 6). CIN status is defined as evidence of gain or loss of chromosomal copy number for >80% of the chromosomal length, with 290,432 stable cells, 11,100 cells bearing chromosomal loss, 5,852 cells bearing chromosomal gain, and 4,541 cells bearing gain and loss of chromosomes. ISR score is defined as the sum of z-normalized expression of ISR marker genes where increased values indicate stronger ISR activation.

10 Figure S8. Mitochondrial genome regulation, related to Figures 6 and 7
(A) Clustering mitochondrial perturbations by nuclear transcriptional response. CRISPRi enables knockdown of nuclear-encoded genes whose protein products are targeted to mitochondria (mitochondrial perturbations). Mitochondrial perturbations were annotated by MitoCarta3.0 and subset to those with a strong transcriptional phenotype (n = 140 mitochondrial perturbations). Gene expression profiles were restricted to nuclear-encoded genes (including 99% of mitochondrial proteins). The heatmap displays the Pearson correlation between pseudobulk *z*-normalized gene expression profiles of mitochondrial perturbations in RPE1 cells. Genetic perturbations are ordered by HDBSCAN with a correlation metric.
(B) Comparing variability in the mitochondrial transcriptome by perturbation localization. The mitochondrial genome encodes 13 protein-coding genes. Genetic perturbations were grouped based on localization of their protein products as determined by the Human Protein Atlas. For each of these 13 mitochondrially encoded genes, the variance in pseudobulk z-normalized expression profiles was calculated between all perturbations with the same localization. Barplots represent the average across genes with 95% confidence interval obtained by bootstrapping.
(C) Variability of gene expression programs from Figure 4B across perturbations. 38 clusters of co-regulated genes were defined via HDBSCAN clustering, and scored within each perturbation. The histogram shows the standard deviation of these scores across the different perturbations in the K562 day 8 experiment.
(D) Activity of mitochondrial genome program in different perturbations. The plot compares growth phenotypes of each perturbation (black dots) to the scores of the mitochondrial genome program in the K562 day 8 experiment. The mitochondrial genome program consists of the 13 protein-coding mitochondrial genes, plus two MT-RNR2-like pseudogenes encoded in the nuclear genome. Negative/positive scores indicate upregulation/downregulation of the program relative to cells with non-targeting sgRNAs (orange dots). Blue lines indicate local estimate of the ±2 standard deviations range of the dataset. Labels indicate the most outlying perturbations, with cyan labels indicating genes with known functions in mitochondria.
(E) Clustering mitochondrial perturbations by mitochondrial transcriptional response. Mitochondrial perturbations were annotated by MitoCarta3.0 and subset to those with a strong transcriptional phenotype as above (n = 140 mitochondrial perturbations). Gene expression profiles were restricted to the 13 mitochondrial-encoded genes. The heatmap displays the Pearson correlation between pseudobulk *z*-normalized gene expression profiles of mitochondrial perturbations in RPE1 cells. Genetic perturbations are ordered by HDBSCAN with a correlation metric.
(F) Comparison of predictive accuracy of nuclear versus mitochondrial genome response. To assess the specificity of the nuclear and mitochondrial genome regulation, random forest classifiers were trained on gene expression profiles to classify cells as having perturbation to one of four mitochondrial complexes (‘‘mtLSU’’ which corresponds to components of the mitochondrial large ribosomal subunit; ‘‘mtSSU’’ which corresponds to components of the mitochondrial small ribosomal subunit; ‘‘complex V’’ which corresponds to components of ATP synthase; ‘‘proteostasis’’ which corresponds to essential proteostatic machinery; ‘‘control’’ which corresponds to non-targeting control cells). In K562 cells (day 8), the top 100 gene features led to an accuracy of 25% for the nuclear-encoded genes, versus 64% for the 13 mitochondrial-encoded genes. As a visual guide, the plot displays the UMAP embedding of single cells colored by perturbed mitochondrial complex based on sgRNA assignment.
(G) Clustering of *TMEM242* genetic perturbation based on the mitochondrial transcriptome. Genetic perturbations to members of ATP synthase and complex I of the respiratory chain were compared with knockdown of *TMEM242*, a mitochondrial gene of unknown function. Gene expression profiles were restricted to the 13 mitochondrially encoded genes. The heatmap displays the Pearson correlation between pseudobulk *z*-normalized gene expression profiles of mitochondrial perturbations in RPE1 cells. Multiple independent sgRNA pairs targeting *TMEM242* were used. Genetic perturbations are ordered by HDBSCAN with a correlation metric.

11

12

13

14

## Figures and Tables

**Table:** KEY RESOURCES TABLE

REAGENT or RESOURCE	SOURCE	IDENTIFIER
Antibodies		
α-INTS10	Abcam	Cat# ab180934s
α-INTS13	Bethyl	Cat# A303-575A; RRID: AB_11125549
α-INTS14	Prestige	Cat# HPA040651
α-C7orf26	Prestige	Cat# HPA052175
α-His	CST	Cat# 2366
α-Mouse	Licor	Cat# 926-32210
α-Rabbit	Licor	Cat# 926-32213
α-CD11b AF647	Biolegend	Cat# 101220; RRID: AB_493546
Bacterial and virus strains		
MegaX Competent Cells	ThermoFisher	Cat# C640003
Stellar Competent Cells	Takara	Cat# 636766
Chemicals, peptides, and recombinant proteins		
TransIT-LT1 Transfection Reagent	Mirus Bio	Cat# MIR2300
Critical commercial assays		
Chromium Single-Cell 3ʹ v3 with Feature Barcoding	10x Genomics	PN-1000075, PN-1000153, PN-1000079
Seahorse XF Cell Mito Stress Test	Agilent	Cat# 103015-100
Bioanalyzer RNA nano	Agilent	Cat# 5067-1511
Deposited data		
Raw sequencing data from Perturb-seq screens	This paper	SRA BioProject PRJNA831566
Processed data from Perturb-seq screens	This paper	http://gwps.wi.mit.edu
Experimental models: Cell lines		
K562 dCas9-BFP-KRAB	Gilbert et al., 2014	N/A
RPE1 Zim3-dCas9	This study	N/A
Oligonucleotides		
Dual sgRNA gDNA primer: AATGATACGGCGACCACCGAGATCTACACCGCGGTCTGTATCCCTTGGAGAACCACCT	Nunéz et al., 2021	oJR324
Dual sgRNA gDNA index primer: CAAGCAGAAGACGGCATACGAGATnnnnnGCGGCCGGCTGTTTCCAGCTTAGCTCTTAAA	Nunéz et al., 2021	oJR325
Custom R1 sequencing primer: CGCGGTCTGTATCCCTTGGAGAACCACCTTGTTGG	Nunéz et al., 2021	oJR326
Custom R2 sequencing primer: GCGGCCGGCTGTTTCCAGCTTAGCTCTTAAAC	Nunéz et al., 2021	oJR328
Custom Index Read 1 sequencing primer: GTTTAAGAGCTAAGCTGGAAACAGCCGGCCGC	Nunéz et al., 2021	oJR327
Recombinant DNA		
See Table S9 for plasmids used in this study	This paper	N/A
Software and Algorithms		
CellRanger 4.0.0	10X Genomics, Inc.	http://software.10xgenomics.com
sgRNA assignment scripts	Replogle et al., 2020	https://github.com/josephreplogle/guide_calling
Perturb-seq analysis codebase	Norman et al., 2019	https://github.com/thomasmaxwellnorman/Perturbseq_GI

## Highlights

- Perturb-seq maps the transcriptional effects of genetic perturbations at genome scale
- Transcriptional signatures allow prediction of function for thousands of genes
- Single-cell RNA-seq enables the study of complex composite phenotypes like aneuploidy
- Analysis of mitochondrial genome expression reveals diverse, stress-specific regulation
