# Supplementary material for: Mapping information-rich genotype-phenotype landscapes with genome-scale Perturb-seq
Source: Cell. Author manuscript; Available in PMC 2022 Aug 16. (PMC9380471; doi:10.1016/j.cell.2022.05.013)

Data S1. Supplementary Data on normalization and sequencing, related to Figure 1.

Figure i: Schematic and performance of internal normalization of gene expression measurements, related to Figure 1.

A) Batch effects in raw data. The K562 day 8 genome-wide experiment was conducted across 273 separate lanes of 10x Genomics droplet single-cell RNA sequencing ("gemgroups"). The plot shows mean expression profiles of highly expressed genes (>2 UMI per cell) within all cells in each gemgroup. The data is normalized (i) for sequencing depth of each gemgroup and (ii) so that the mean expression of each gene is 1 across all gemgroups. Colors at the top indicate different levels of multiplexing that were present within the experiment: including groups of samples (generally in sets of 8) that went through scRNA-seq together, 96 well plates used for library preparation, and separate lanes used during Illumina sequencing. The range of the heatmap is set according to the 2%-98% quantiles of the data.
B) Expression following internal normalization. We rescale gene expression by *z*-normalizing relative to the control cells (containing non-targeting sgRNAs, ~4% of all cells) within the same gemgroup. The plot shows the average normalized expression of all cells within each gemgroup following this procedure, with the range of the heatmap set according to the 2%-98% quantiles of the data. By construction control cells have mean expression 0 and standard deviation 1 in this scale. Genes are presented in the same order as in panel (A).

Figure ii: Validation of Ultima Genomics ultra-high throughput sequencing, related to Figure 1.

A) Heatmap of perturbation relationships as derived from independent Ultima and Illumina sequencing data. We analyzed 2,017 genetic perturbations that elicited strong phenotypes. Pearson correlations were used to summarize perturbation-perturbation relationships calculated on *z*-normalized gene expression profiles across 2,059 highly variable genes. Genetic perturbations were ordered by HDBSCAN with a correlation metric calculated on the Illumina data (upper triangle) and the Ultima data (lower triangle) were ordered identically for comparison.
B) Comparison of perturbation relationships as derived from Ultima and Illumina sequencing data. Perturbation-perturbation relationships were calculated as described in fig. S13A. The cophenetic correlation between the Illumina and Ultima datasets is r=0.99.
C) Comparison of perturbation profiles derived from Ultima and Illumina sequencing. We computed the pairwise correlation of gene expression profiles of 2,017 genetic perturbations that elicited strong phenotypes. The mean correlation of profiles was r=0.73. Perturbations that elicited a higher number of differentially expressed genes (DEGs) (determined using a two-sample Anderson-Darling test compared against non-targeting guides) were better correlated across sequencing platforms (Spearman's rho=0.90).

Data S1 - Figure i

A

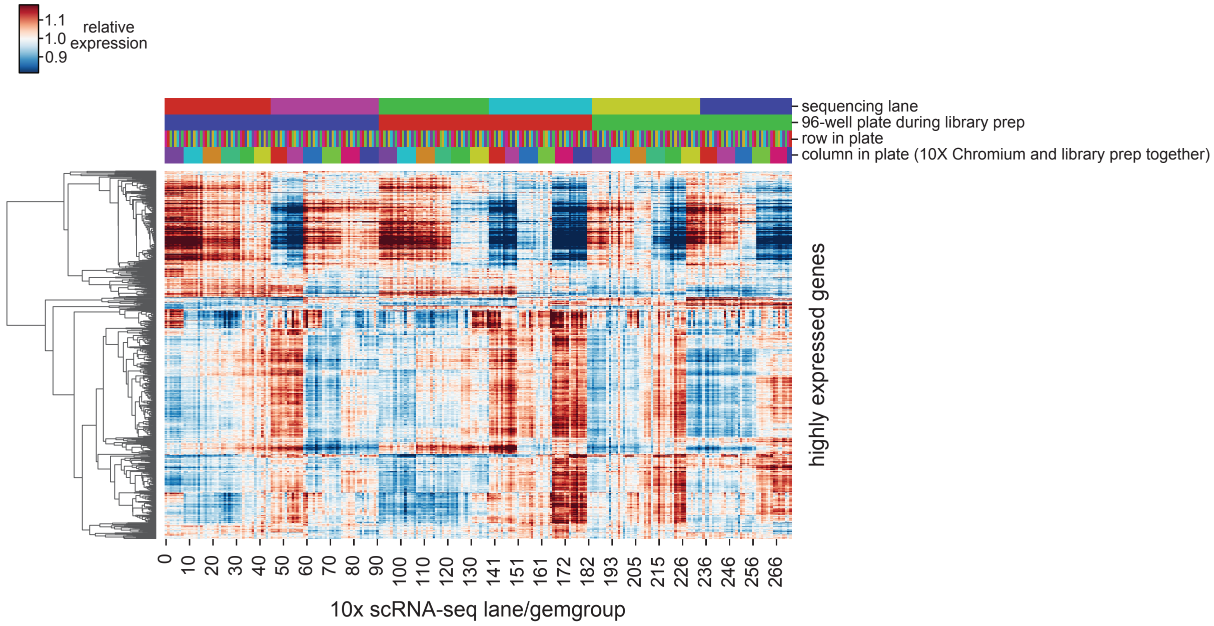

1) Identify core set of non-targeting controls (514 sgRNAs)
2) Filter cells based on UMI count and mtDNA content
3) Scale cellular UMI content so that average UMI content in control cells is constant across all gemgroups.
4) *z*-normalize expression within each gemgroup relative to control cells in that gemgroup

B

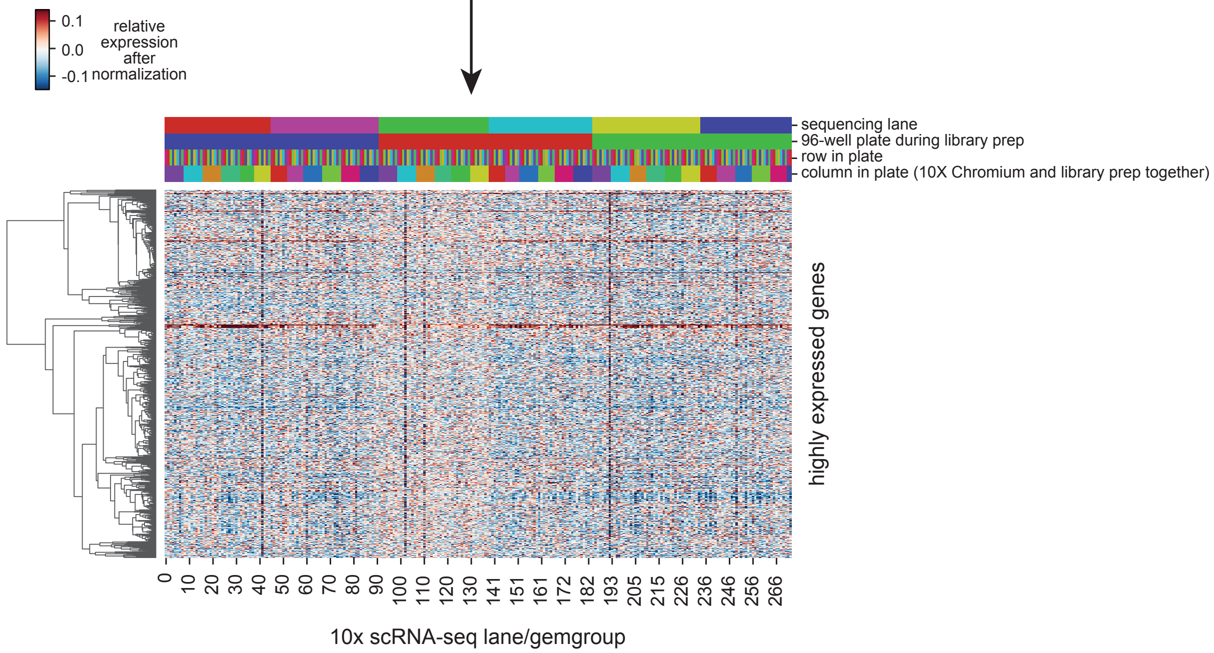

Data S1 - Figure ii

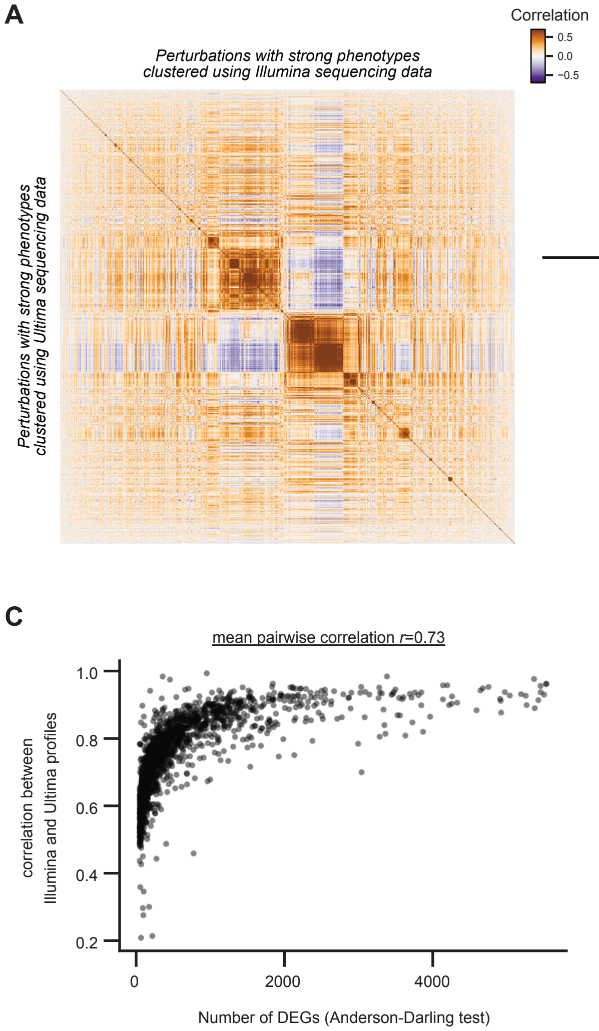

Supplement: 1 [file NIHMS1812939-supplement-1.pdf]
