# Supplementary material for: Mapping information-rich genotype-phenotype landscapes with genome-scale Perturb-seq
Source: Cell. Author manuscript; Available in PMC 2022 Aug 16. (PMC9380471; doi:10.1016/j.cell.2022.05.013)

Data S2. Supplementary Data from investigation of the Integrator complex, related to Figure 3.

Figure i: Supplementary data related to the functional modules of the Integrator complex, related to Figure 3.

A) Relationship between Integrator complex members and *C7orf26* in RPE1 cells. Multiple independent sgRNA pairs were used to target *INTS13*, *INTS14*, and *C7orf26*, with independent guides indicated by numbers next to gene names (e.g. C7orf26-1, C7orf26-2, etc.). The heatmap displays the Pearson correlation between pseudobulk *z*-normalized gene expression profiles of Integrator complex members. Genetic perturbations are ordered by average linkage hierarchical clustering based on correlation.

B) SEC trace and full Western blots for purification of a INTS10-INTS13-INTS14-C7orf26 complex. His-INTS10, INTS13, INTS14, and C7orf26 were overexpressed in Expi293 cells, affinity purified, and separated via SEC. The INTS10-INTS13-INTS14-C7orf26 proteins co-fractionated as a higher molecular weight species as visualized by Western blotting.

C) and D) Identification of differentially expressed genes between Integrator complex modules in K562 cells (C) and RPE1 cells (D). Random forest classifiers were trained on gene expression profiles to classify cells as having perturbation to one of three Integrator complex modules (“endonuclease”, “shoulder and backbone” or “10-13-14-C7orf26”). The top 30 gene features led to an accuracy of 97% in K562 cells and 89% in RPE1 cells. Heatmap displays the z-scored gene expression of the top 30 features in each cell type.

E) Comparison of PRO-seq active RNA polymerase II gene expression profiles across genetic perturbations. PRO-seq reads were aligned to the transcriptome and depth normalized. Heatmap shows the Spearman correlation of expression profiles with biological replicates indicated (e.g. INTS5_rep1 and INTS5_rep2).

Figure ii: Integrator biochemistry, related to Figure 3.

A) Full blots visualizing the effects of CRISPRi-based depletion of Integrator subunits with different probes.

B) Full blots visualizing INTS10 pulldown with different probes.

C) Full blots visualizing Integrator SEC purification with different probes.

Figure iii: Integrator biochemistry in Drosophila, related to Figure 3.

A) Heatmap of results from co-immunoprecipitation of Integrator complex components in Drosophila. Heatmaps were generated using average spectral counts resulting from triplicate pulldowns that were each normalized to total spectra.

B) Volcano plot of enrichments from co-immunoprecipitation using nuclear extract derived from S2 cells stably expressing FLAG-tagged CG5274, which is the Drosophila orthologue of *C7orf26*. Enrichment was calculated using spectral counts and significance was determined from three independent purifications.

C) Co-immunoprecipitation of Integrator complex components in Drosophila. Cell lysates were affinity purified and select Integrator proteins were probed by western blot.

Data S2 - Figure i

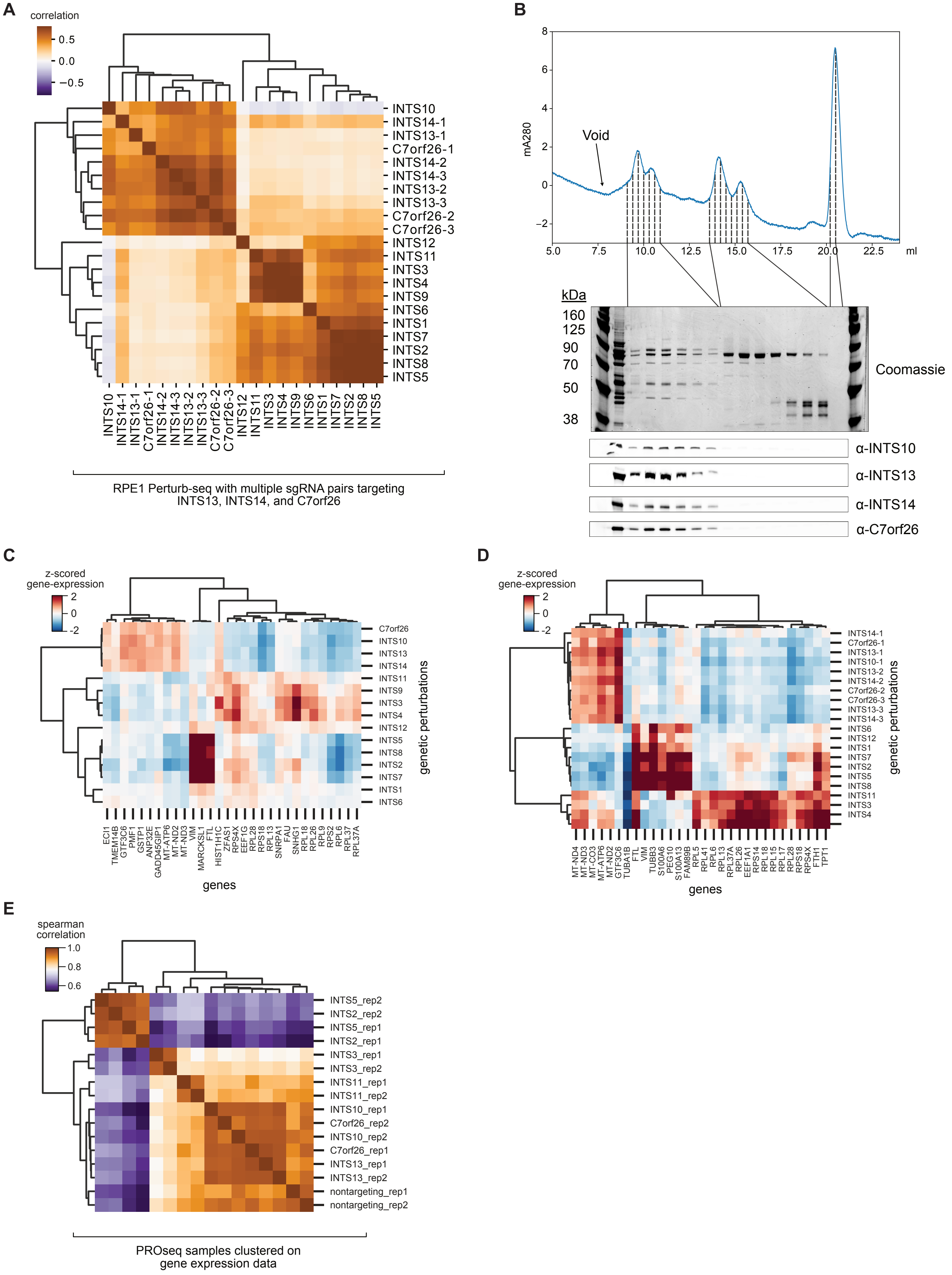

Data S2 - Figure ii

A

Integrator Depletion

α-rabbit secondary
Revert® Total Protein
Chameleon® Duo Ladder

Samples in order shown
in main text figure

Solid Blue: antibody crops
Dotted blue: total protein crop

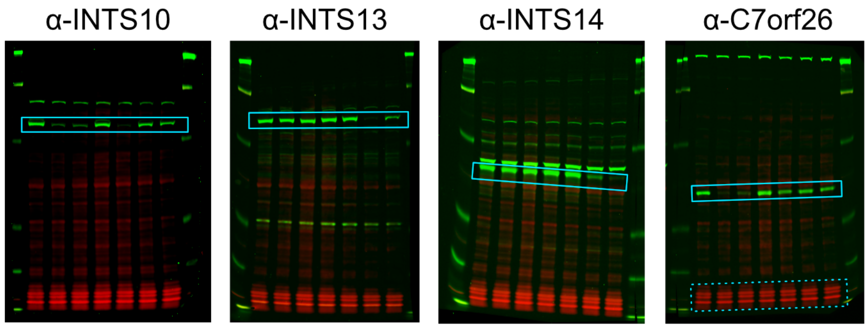

B

Integrator Pulldown

α-rabbit secondary
Revert® Total Protein
Chameleon® Duo Ladder

Samples in order shown
in main text figure

Solid Blue: antibody crops
Dotted blue: total protein crop

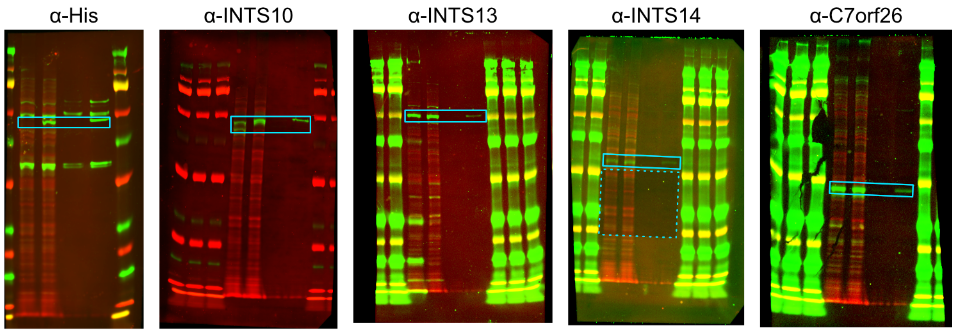

C

Integrator Purification

Readyblue™

α-rabbit secondary
Chameleon® Duo Ladder

Samples in order shown
in supplementary SEC figure

Solid Blue: main text crops
Dotted blue: supplementary crops

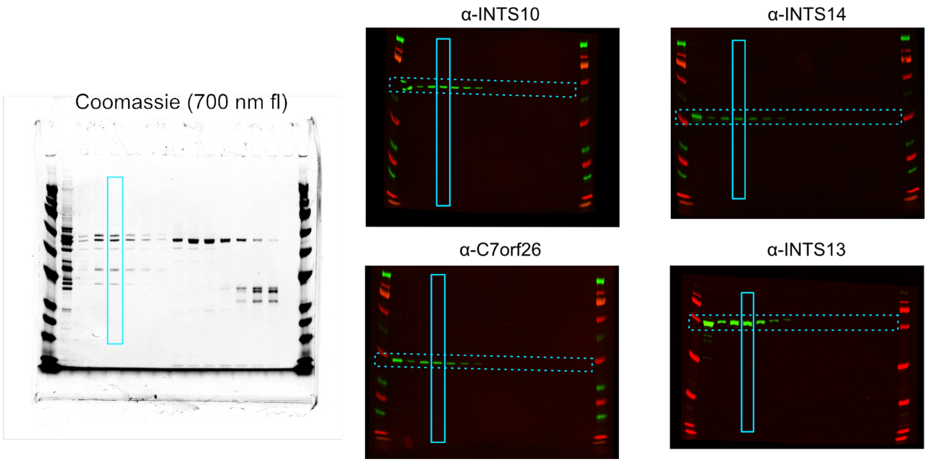

Data S2 - Figure iii

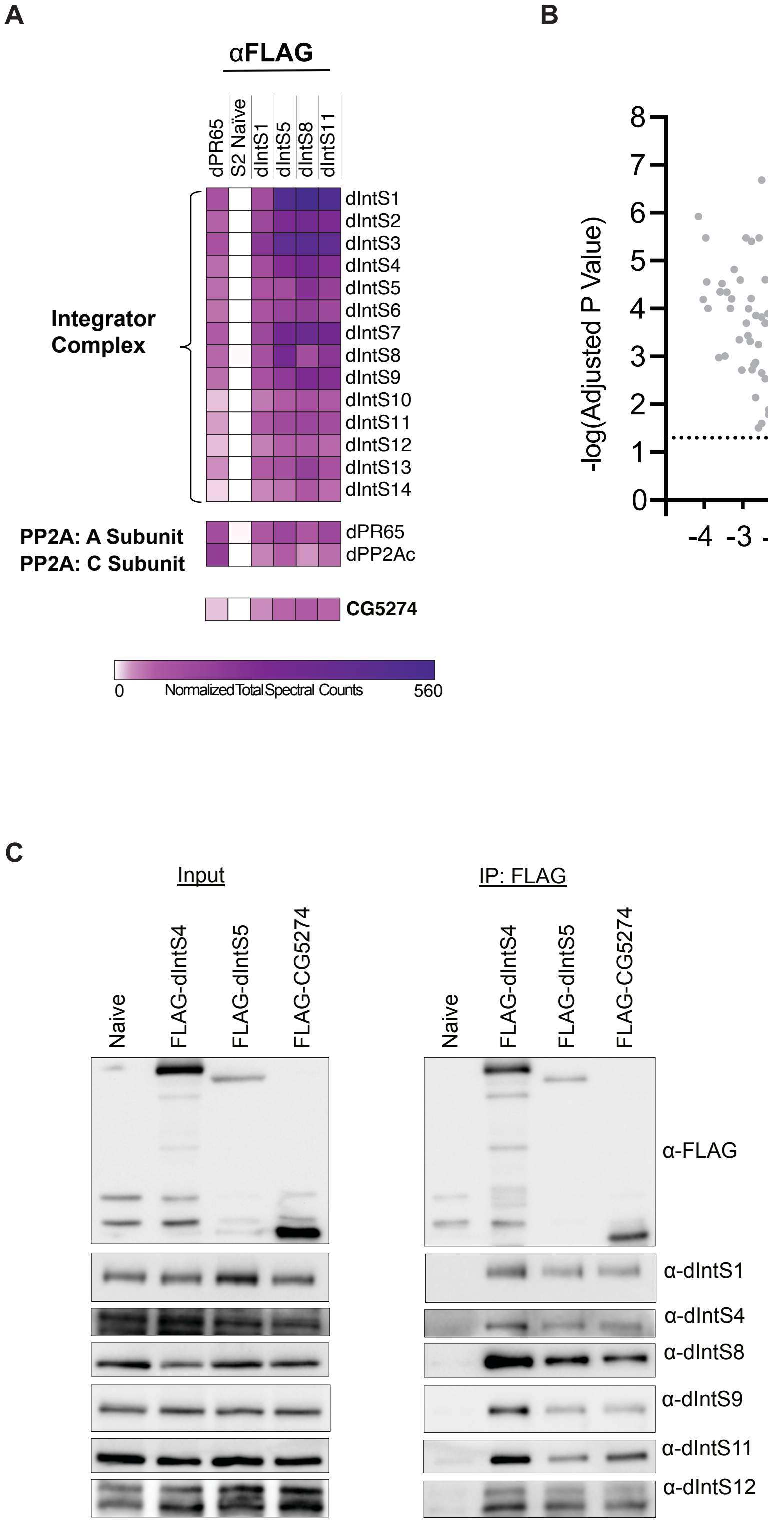

Supplement: 2 [file NIHMS1812939-supplement-2.pdf]
