# Supplementary figures and images for: Mapping information-rich genotype-phenotype landscapes with genome-scale Perturb-seq
Source: Cell. Author manuscript; Available in PMC 2022 Aug 16. (PMC9380471; doi:10.1016/j.cell.2022.05.013)

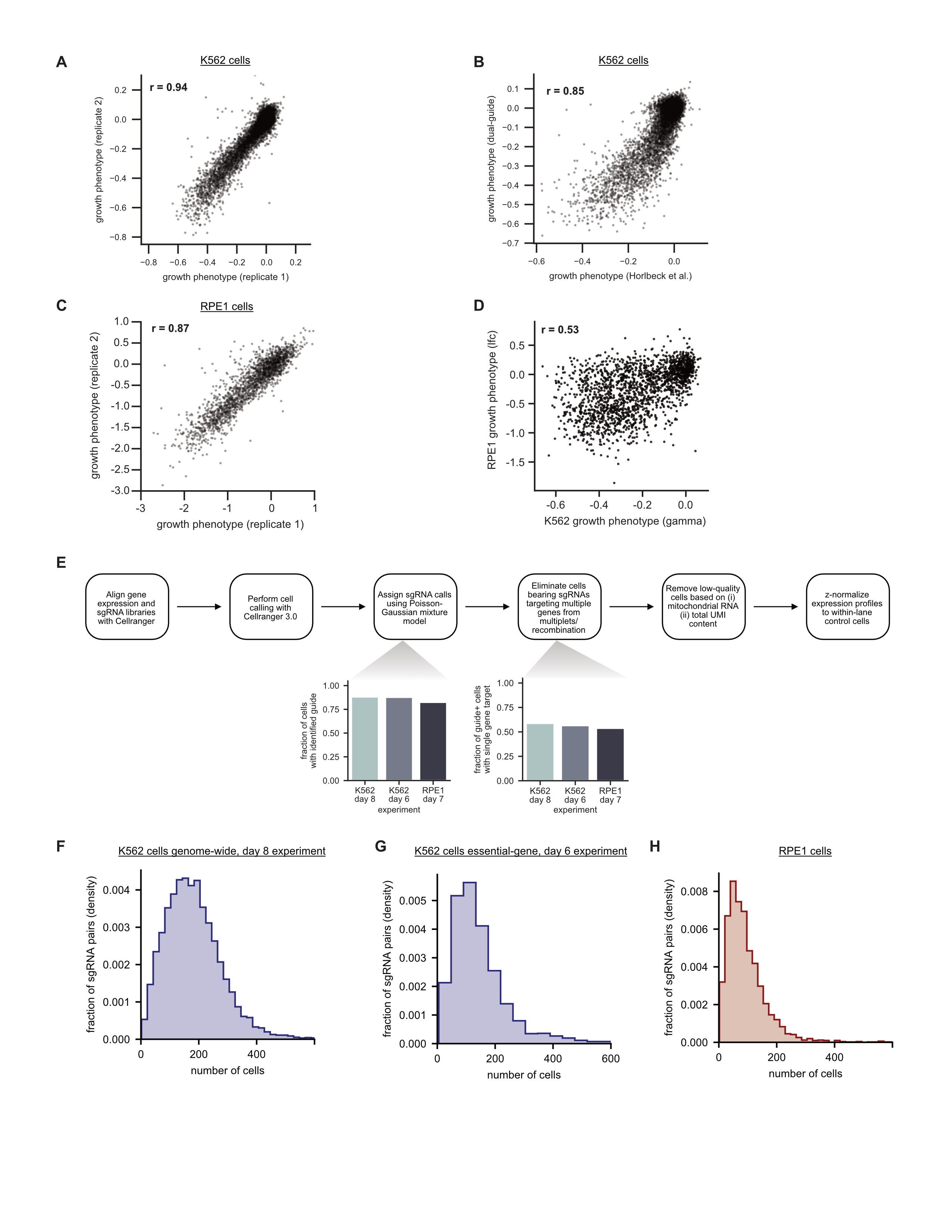

Supplement: 3 — Figure S1. Growth screens, filtering, and coverage, related to Figure 1 (A) Comparing the growth phenotypes of dual-sgRNA constructs between growth screen replicates in K562 cells. Growth phenotypes are reported as the log2 guide enrichment per cell doubling (gamma) between day 6 and day 16 post library transduction. Replicates are strongly correlated (n = 11,056 dual-sgRNA constructs; r = 0.94). For 50 outlier genes (where the residual from a regression comparing replicates was >0.2), the growth phenotype was set to missing. (B) Benchmarking the growth phenotypes of dual-sgRNA constructs to single-sgRNA screens. Growth phenotypes (gammas) are compared between the dual-sgRNA library compared with the mean of the best three sgRNAs from Horlbeck et al. The screens are strongly correlated (n = 9,386 genes after excluding constructs mapping to secondary transcription start sites [TSSs]; r = 0.85) but with stronger growth phenotypes observed for the dual-sgRNA library. (C) Comparing the growth phenotypes of dual-sgRNA constructs between growth screen replicates in RPE1 cells. Growth phenotypes are reported as the log2 guide enrichment between the plasmid library and day 7 post library transduction. Replicates are strongly correlated (n = 2,203 constructs targeting common essential genes; r = 0.87). For 19 outlier genes (where the residual from a regression comparing replicates was >1), the growth phenotype was set to missing. (D) Comparing growth phenotypes between K562 and RPE1 cells. Growth phenotypes are correlated (n = 1,951 constructs; r = 0.53) despite substantial differences in screen time point (day 6 to day 16 for K562 cells versus day 0 to day 7 in RPE1 cells). (E) Schematic overview of data alignment, cell calling, sgRNA assignment, and filtering. (F) Histogram of the number of cells per genetic perturbation in the K562 day 8 genome-wide Perturb-seq experiment. The number of detected genetic perturbations (expected sgRNA pairs) was n = 11,258, with a mean cove [file NIHMS1812939-supplement-3.tiff]

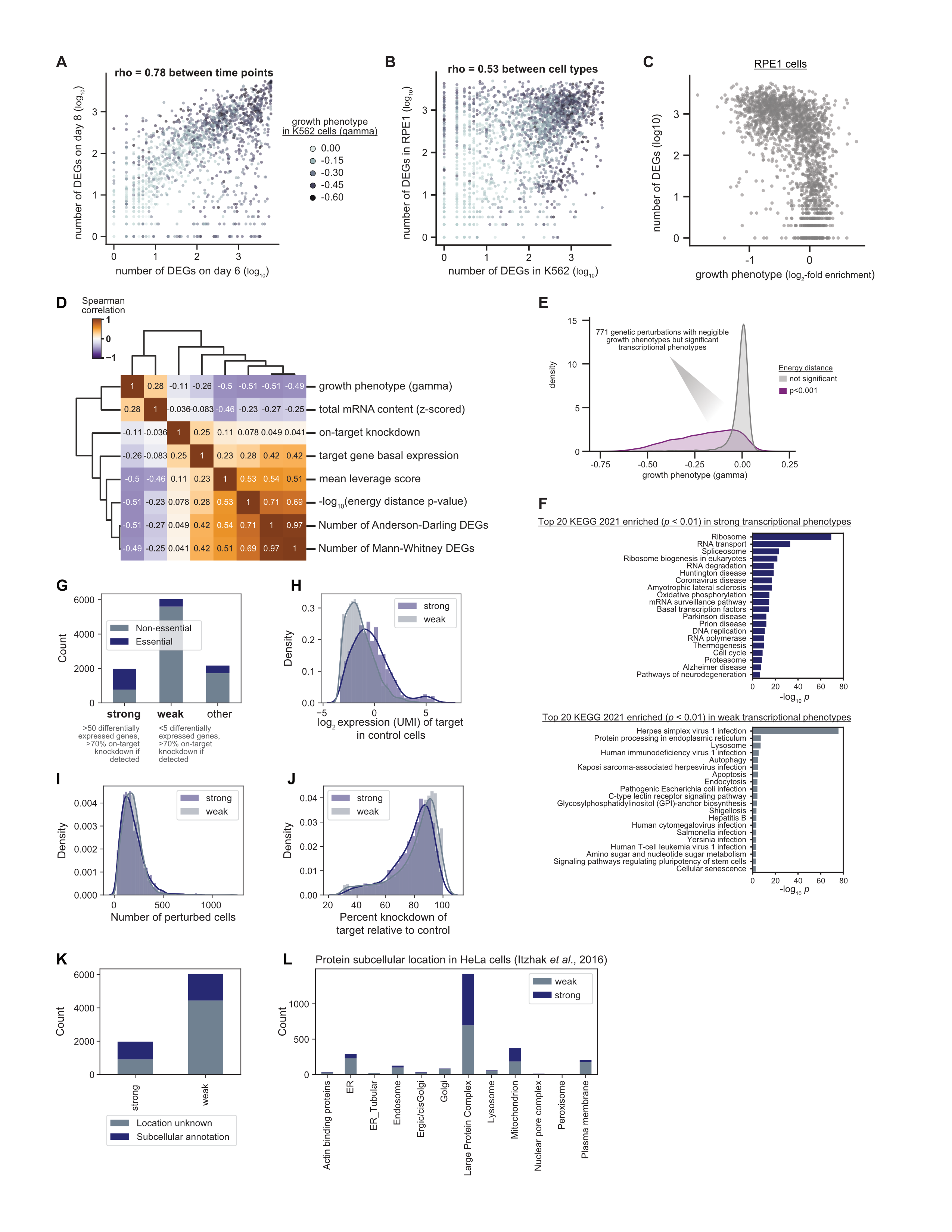

Supplement: 4 — Figure S2. Differential expression and enrichment analyses, related to Figure 1 (A) Relationship between the number of differentially expressed genes (DEGs) for a genetic perturbation in K562 cells at day 8 versus day 6 post-transduction. DEGs were determined using a two-sample Anderson-Darling test comparing against non-targeting guides (n = 2,276 common genetic perturbations, Spearman’s rho = 0.78). (B) Relationship between the number of DEGs for a genetic perturbation in K562 cells (day 8 genome-wide dataset) versus RPE1 cells. DEGs were determined using a two-sample Anderson-Darling test comparing against non-targeting guides (n = 2,636 common genetic perturbations, Spearman’s rho = 0.53). (C) Comparing the growth phenotype versus the number of DEGs for each multiplexed guide pairs in RPE1 cells. Growth phenotypes are reported as the log2 guide enrichment between day 0 and day 7 post-lentiviral transduction. DEGs were determined using a two-sample Anderson-Darling test comparing against non-targeting guides. (D) Relationship between features of genetic perturbations in K562 cells genome-wide day 8 Perturb-seq. The features were calculated as detailed in STAR Methods. The heatmap displays Spearman correlations between features. (E) The distribution of growth phenotypes in genetic perturbations with a transcriptional phenotypes in K562 cells genome-wide day 8 Perturb-seq. Histogram (kernel density estimate) comparing the growth phenotype in K562 cells (gamma) of genetic perturbations to the permuted energy distance test. 771 genetic perturbations had a gamma >—0.1 (considered a negligible effect on cellular growth) but a significant transcriptional phenotype. (F) KEGG pathway enrichment for genetic perturbations causing strong and weak transcriptional phenotypes in the K562 day 8 dataset. Strong perturbations were defined as having (i) >50 differentially expressed genes at a significance of p < 0.05 by Anderson-Darling test following Benjamini-Hochberg correction [file NIHMS1812939-supplement-4.tiff]

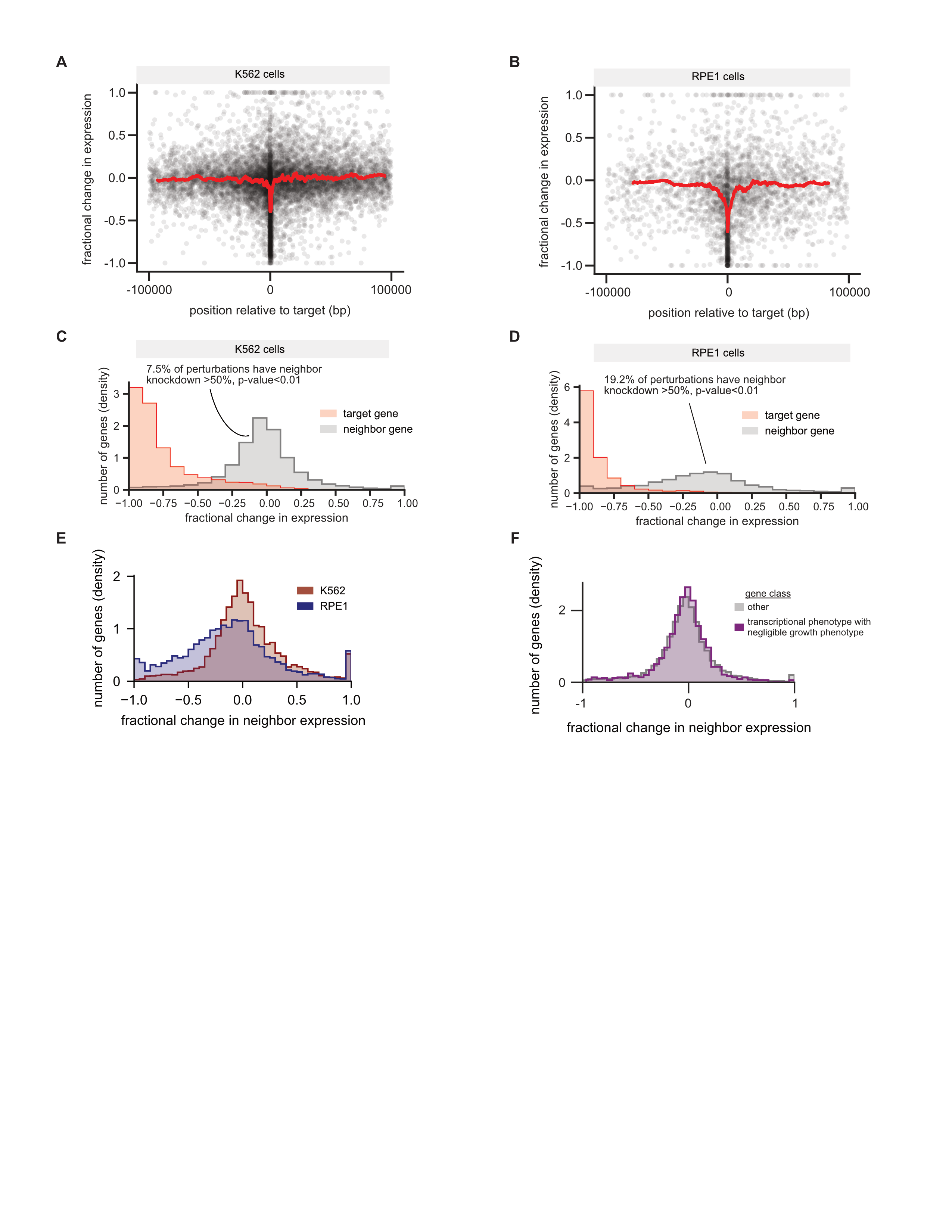

Supplement: 5 — Figure S3. Assessing neighbor gene off-target knockdown in Perturb-seq data, related to Figure 1 (A and B) Relationship between neighbor gene off-target knockdown and position relative to the target gene in K562 cells (day 8) (A) and RPE1 cells (B). For each target genes, the two neighbor genes are defined as the gene immediately upstream and downstream (at an expression >0.1 UMI per cell). The position relative to the target is the distance of either the start or end of the neighbor gene (whichever is closer) to the start of the target gene. The fractional change in expression is defined as the expression in the targeted cells minus the expression in non-targeting cells, relative to the expression in the non-targeting cell population (—1 implies 100% knockdown). (C and D) Comparison between target gene and neighbor gene knockdown in K562 cells (day 8) (C) and RPE1 cells (D). p values are assigned by comparing a bootstrap test. (E) Comparison of neighbor gene knockdown in K562 cells (day 8) versus RPE1 cells. (F) Comparison of neighbor gene knockdown based on transcriptional phenotype in K562 cells (day 8). Perturbations with ‘‘transcriptional phenotype with negligible growth phenotype’’ are those perturbations where gamma >—0.1 that had a significant transcriptional phenotype by the permuted energy distance test. [file NIHMS1812939-supplement-5.tiff]

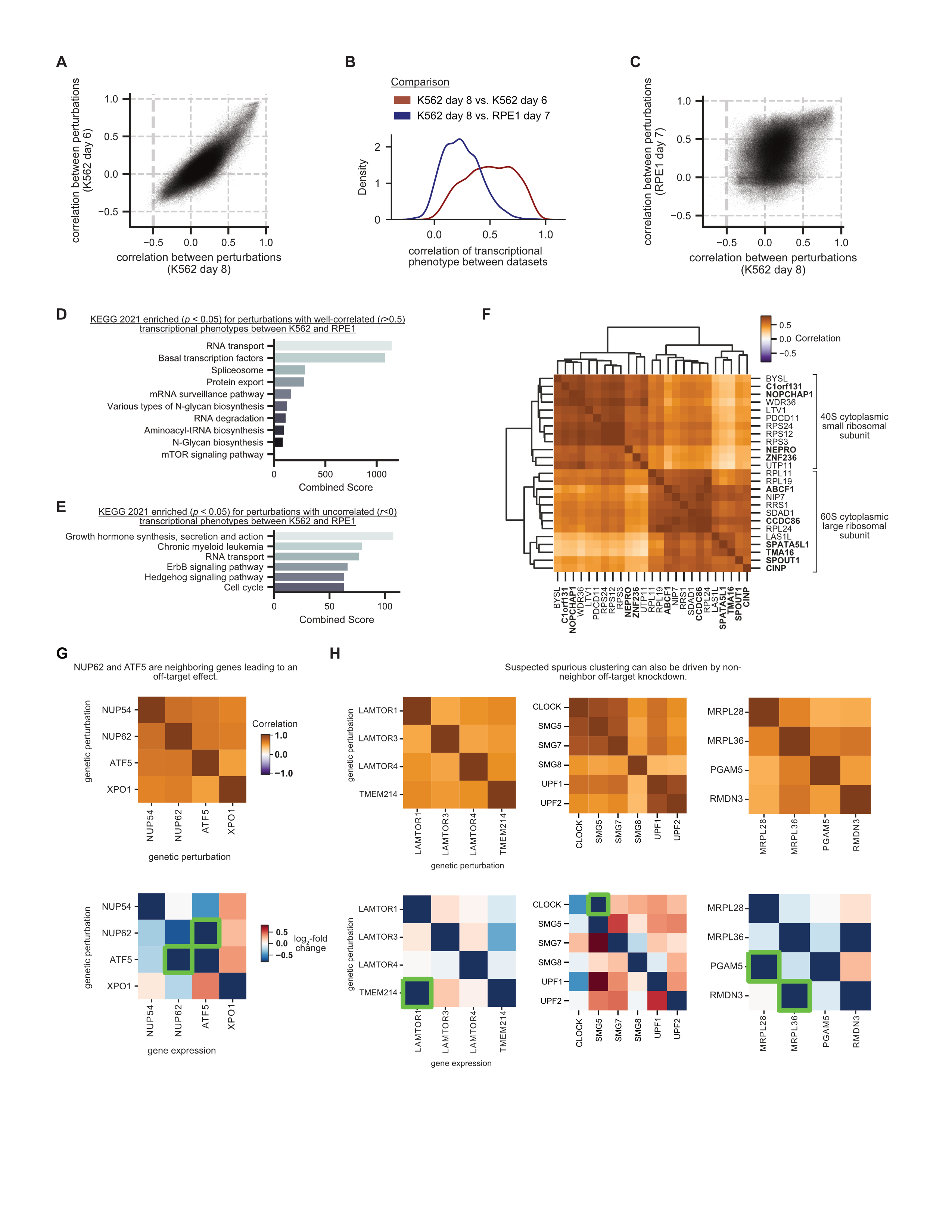

Supplement: 6 — Figure S4. Defining gene function with Perturb-seq, related to Figure 2 (A) Comparison of perturbation relationships as derived from independent K562 day 8 and K562 day 6 datasets. We analyzed the union of 1,206 genetic perturbations that elicited transcriptional phenotypes (>10 differentially expressed genes) in all three datasets. Pearson correlations were used to summarize perturbation-perturbation relationships calculated on z-normalized gene expression profiles across well-expressed (>0.25 UMIs per cell) and highly variable genes. The cophenetic correlation between datasets is r = 0.82. (B) Correlation of transcriptional phenotypes between datasets. We analyzed the intersection of genetic perturbations that elicited transcriptional phenotypes (>10 differentially genes) between each pair of datasets. For each genetic perturbation, we calculated the Pearson correlation of z-normalized gene expression profiles across well-expressed (>0.5 UMIs per cell) genes. The comparison of K562 day 8 versus K562 day 6 (red) and K562 day 8 versus RPE1 day 7 (blue) are shown. (C) Comparison of perturbation relationships as derived from independent K562 day 8 and RPE1 datasets. We analyzed the union of 1,206 genetic perturbations that elicited transcriptional phenotypes (>10 differentially expressed genes) in all three datasets. Pearson correlations were used to summarize perturbation-perturbation relationships calculated on z-normalized gene expression profiles across well-expressed (>0.25 UMIs per cell) and highly variable genes. The cophenetic correlation between datasets is r = 0.37. (D) KEGG pathway enrichment for genetic perturbations with well-correlated (r > 0.5) transcriptional phenotypes between the K562 day 8 and RPE1 day 7 datasets. Enrichr was used to perform enrichment in the KEGG 2021 pathways with the significant (adjusted p < 0.05) pathways shown. (E) KEGG pathway enrichment for genetic perturbations with uncorrelated (r > 0) transcriptional phenotypes between th [file NIHMS1812939-supplement-6.tiff]

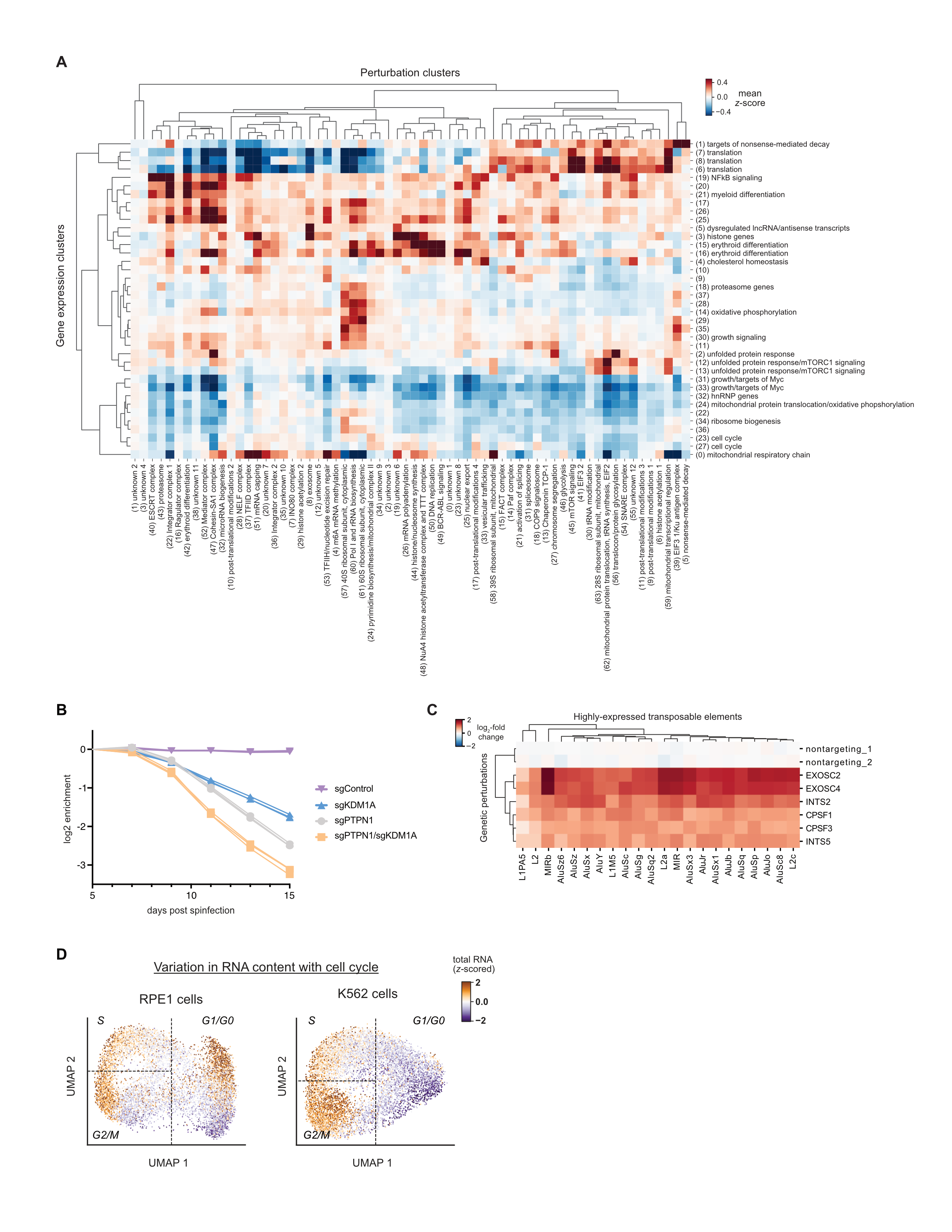

Supplement: 7 — Figure S5. Genotype-phenotype relationships, related to Figure 4 (A) Heatmap of the high-level genotype-phenotype map (identical to Figure 4B with full labels). The heatmap represents the mean Z scored expression for gene expression and perturbation clusters. For a subset of clusters, clustered are labeled with manual annotations (black labels) of cluster function. (B) Growth effect of PTPN1 or KDM1A knockdown in K562 cells. Cells were co-transduced with fluorescently labeled sgKDM1A, sgPTPN1, or a non-targeting control guide. Enrichment was determined by flow cytometry relative to uninfected cells in biological triplicate. (C) Comparison of transposable element expression profiles between top regulators. Heatmap displays log2-fold changes in expression of highly expressed transposable element metagenes (columns) for genetic perturbations (rows) in K562 cells (day 8) Perturb-seq. Genetic perturbations and genes are ordered by average linkage hierarchical clustering with a Euclidean distance metric. (D) Comparison of total RNA content with cell-cycle state. For single cells, cell-cycle positioning was inferred by UMAP dimension reduction on differential expression profiles of 199 selected cell-cycle regulated genes. The dimension reduction was performed independently for RPE1 cells (left) and K562 cells (right). Cell-cycle occupancy is shown as a scatterplot of UMAP positions of a random subset of 10,000 cells per cell type. Approximate gates between cell-cycle phases (G1 or G0; S; G2 or M) are shown as dotted lines. The total RNA content per cell was calculated from the total number of UMIs detected per cell which were Z scored with respect to gemgroup/lane control cells. [file NIHMS1812939-supplement-7.tiff]

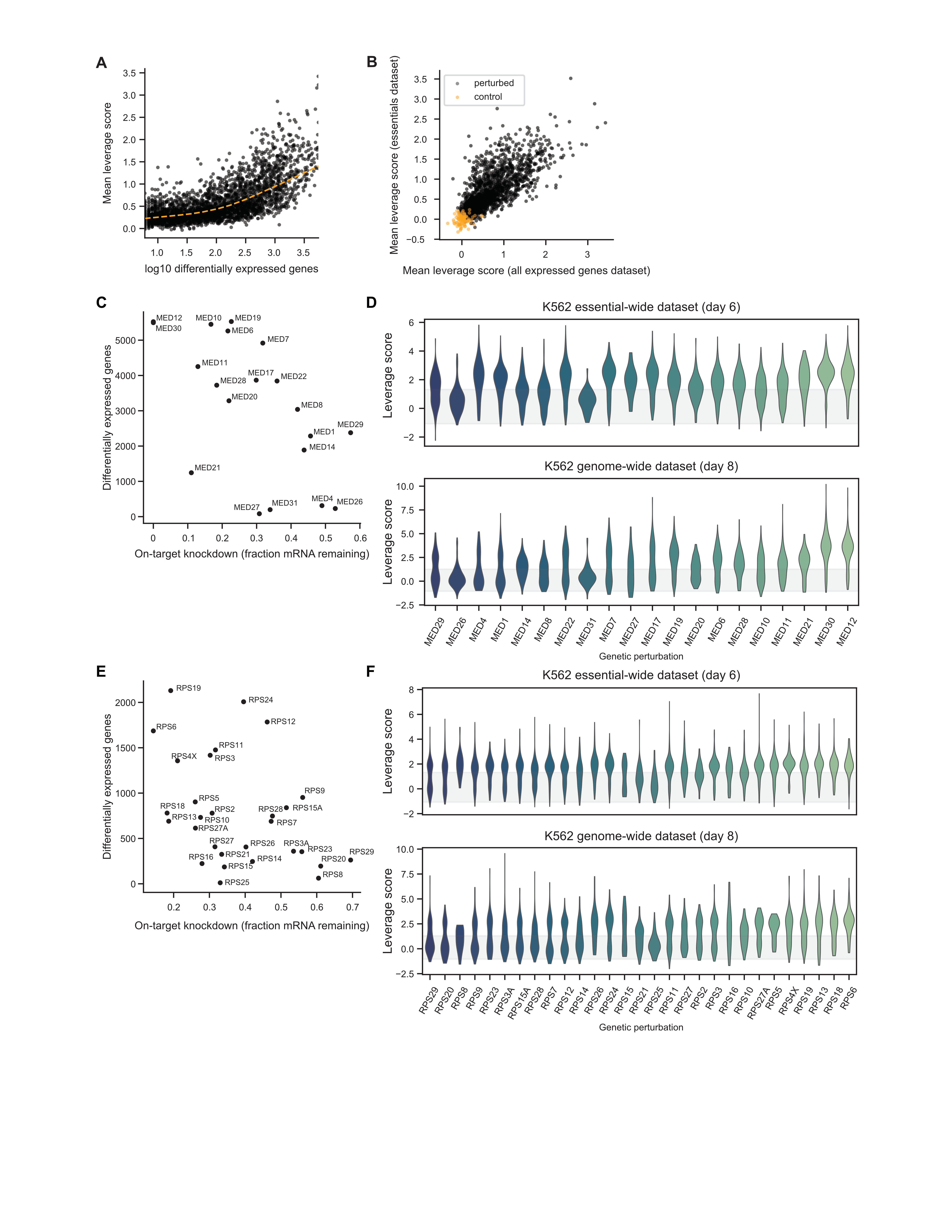

Supplement: 8 — Figure S6. Assessing the penetrance and heterogeneity of response to genetic perturbations, related to Figure 5 (A) We scored how outlying each perturbed cell was relative to non-targeting control cells using leverage scores. The plot compares the mean leverage score for each genetic perturbation to the number of differentially expressed genes detected by the Anderson-Darling test (Spearman’s rho = 0.71). (B) To assess reproducibility of leverage scores, plot compares mean leverage scores of perturbations (black dots) in K562 cells between the essentials dataset (taken at day 6 post-infection) and the dataset targeting all expressed genes (taken at day 8 post-infection). Non-targeting control sgRNAs are in orange (Spearman’s rho = 0.79). (C) Relationship between knockdown of target gene (relative to expression in control cells bearing non-targeting sgRNAs) and number of differentially expressed genes detected by Anderson-Darling test for perturbations targeting subunits of the Mediator complex. (D) Leverage scores distributions of perturbations targeting subunits of the Mediator complex. Plot shows kernel density estimates for each perturbation ordered from least knocked down (left) to most (right). Top panel is within essentials dataset and bottom panel is within the all expressed genes dataset. The gray bar shows the 10%–90% range of leverage scores within control cells bearing non-targeting sgRNAs. (E and F) As in (C) and (D) but for perturbations targeting the small subunit of the ribosome. [file NIHMS1812939-supplement-8.tiff]

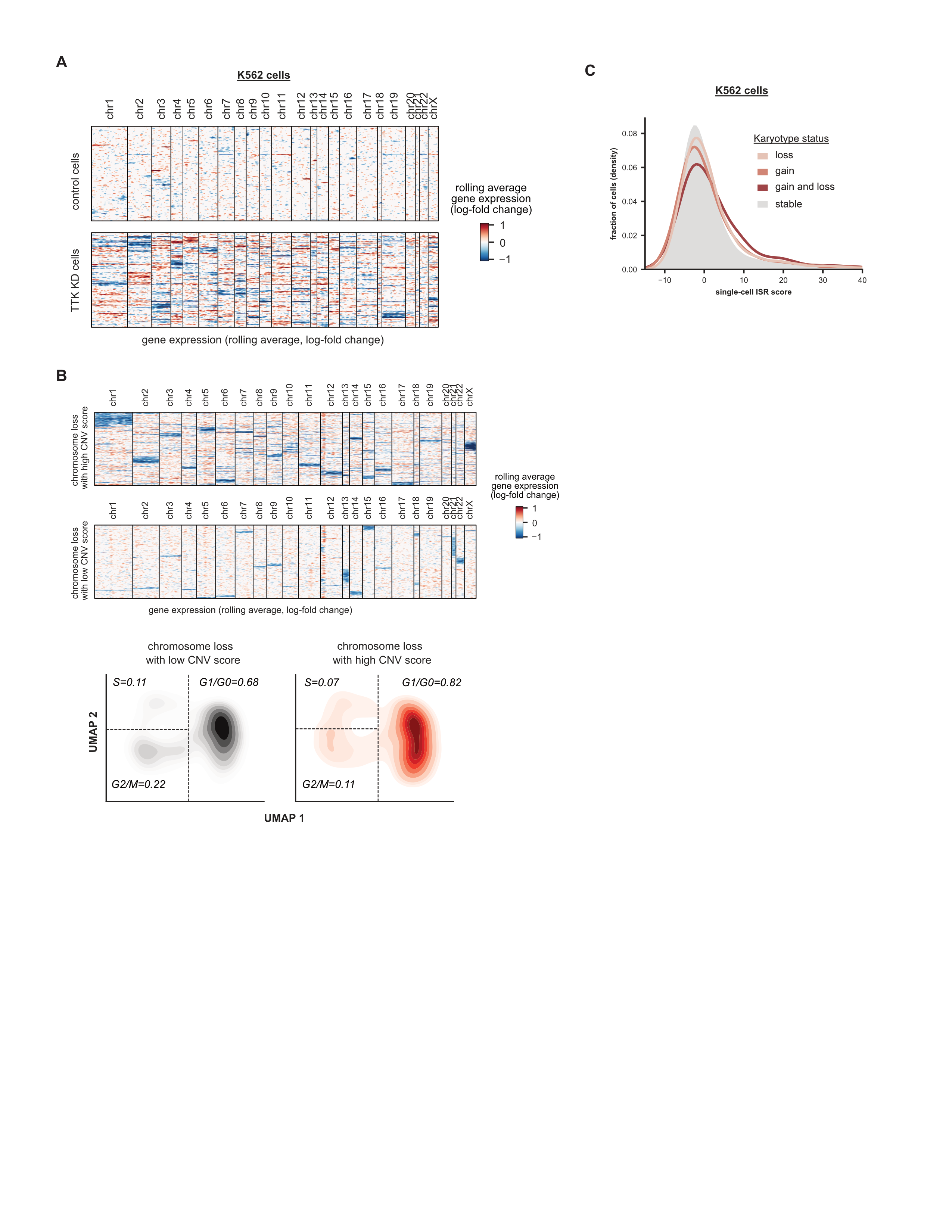

Supplement: 9 — Figure S7. Chromosomal instability, related to Figure 5 (A) Heatmap of chromosomal copy-number inference from Perturb-seq data. For all genes (expressed >0.05 UMI per cell), the log-fold change in expression is calculated with respect to the average of non-targeting control cells, and genes are ordered along the genome. A weighted moving average of 100 genes is used infer copy-number changes (columns) in single cells (rows) with noise and median filtering. 199 TTK knockdown K562 cells and 199 randomly sampled non-targeting control K562 cells are shown (data from K562 essential-wide day 6 dataset). Cells are ordered by average linkage hierarchical clustering based on correlation of chromosomal copy-number profiles. (B) Comparison of cell-cycle effects by magnitude of karyotypic abnormality in RPE1 cells. RPE1 cells with at least one chromosomal loss (defined as evidence of chromosomal loss for >80% of the chromosomal length) were stratified into high, medium, and low degree of karyotypic abnormality based on their CNV score. 500 randomly sampled high and low CNV score cells were visualized in a heatmap of chromosomal copy-number inference. Below, the cell-cycle occupancy of high and low CNV cells is shown for 1,000 randomly sampled cells. For single cells, cell-cycle positioning was inferred by UMAP dimension reduction on differential expression profiles of 199 selected cell-cycle regulated genes. Cell-cycle occupancy is shown as a 2D kernel density estimate of a random subset of 1,000 cells per karyotypic status. Approximate gates between cell-cycle phases (G1 or G0; S; G2 or M) are shown as dotted lines, and the fraction of cells in each cell-cycle phase are indicated. (C) Effect of chromosomal instability (CIN) on activation of the integrated stress response (ISR). Histogram (kernel density estimate) compares the ISR score versus CIN status in K562 cells (day 6). CIN status is defined as evidence of gain or loss of chromosomal copy number for >80% of the chromosom [file NIHMS1812939-supplement-9.tiff]

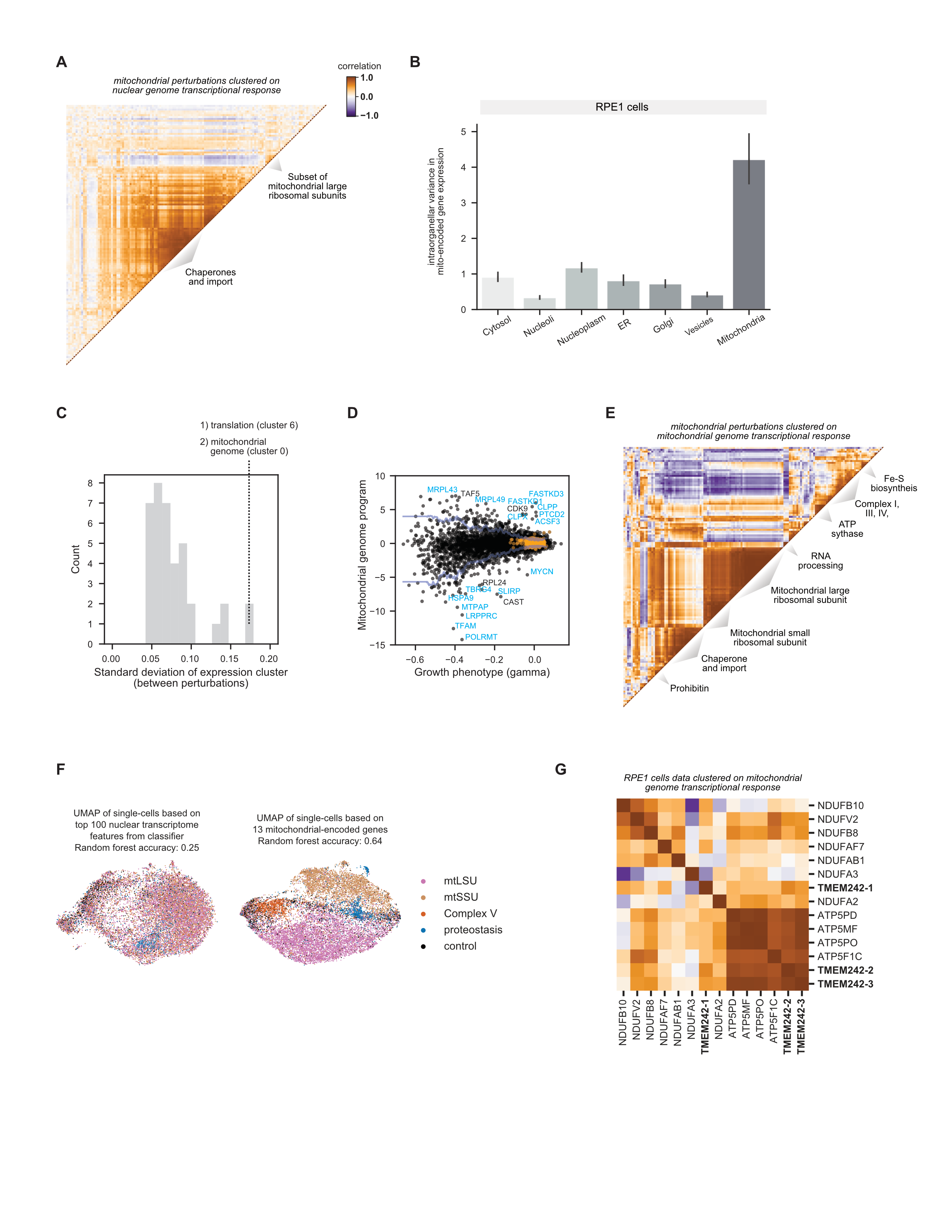

Supplement: 10 — Figure S8. Mitochondrial genome regulation, related to Figures 6 and 7 (A) Clustering mitochondrial perturbations by nuclear transcriptional response. CRISPRi enables knockdown of nuclear-encoded genes whose protein products are targeted to mitochondria (mitochondrial perturbations). Mitochondrial perturbations were annotated by MitoCarta3.0 and subset to those with a strong transcriptional phenotype (n = 140 mitochondrial perturbations). Gene expression profiles were restricted to nuclear-encoded genes (including 99% of mitochondrial proteins). The heatmap displays the Pearson correlation between pseudobulk z-normalized gene expression profiles of mitochondrial perturbations in RPE1 cells. Genetic perturbations are ordered by HDBSCAN with a correlation metric. (B) Comparing variability in the mitochondrial transcriptome by perturbation localization. The mitochondrial genome encodes 13 protein-coding genes. Genetic perturbations were grouped based on localization of their protein products as determined by the Human Protein Atlas. For each of these 13 mitochondrially encoded genes, the variance in pseudobulk z-normalized expression profiles was calculated between all perturbations with the same localization. Barplots represent the average across genes with 95% confidence interval obtained by bootstrapping. (C) Variability of gene expression programs from Figure 4B across perturbations. 38 clusters of co-regulated genes were defined via HDBSCAN clustering, and scored within each perturbation. The histogram shows the standard deviation of these scores across the different perturbations in the K562 day 8 experiment. (D) Activity of mitochondrial genome program in different perturbations. The plot compares growth phenotypes of each perturbation (black dots) to the scores of the mitochondrial genome program in the K562 day 8 experiment. The mitochondrial genome program consists of the 13 protein-coding mitochondrial genes, plus two MT-RNR2-like pseudogenes encoded in the [file NIHMS1812939-supplement-10.tiff]
